# Supplementary material for: A deep dive into the coelacanth phylogeny
Source: PLoS One. 2025 Jun 6;20(6):e0320214. doi: 10.1371/journal.pone.0320214 (PMC12143573; doi:10.1371/journal.pone.0320214)
Supplement: S1 Data — (DOCX) [file pone.0320214.s001.docx]

#NEXUS

BEGIN TAXA;

 DIMENSIONS NTAX=50;

 TAXLABELS

 'Onychodontiformes (Onychodus) '

 'Allenypterus'

 'Atacamaia'

 'Axelia'

 'Axelrodichthys'

 'Caridosuctor'

 'Chinlea'

 'Coccoderma'

 'Coelacanthus'

 'Diplocercides'

 'Diplurus'

 'Dobrogeria'

 'Euporosteus'

 'Foreyia'

 'Garnbergia'

 'Gavinia'

 'Guizhoucoelacanthus'

 'Hadronector'

 'Heptanema'

 'Holophagus'

 'Holopterygius'

 'Indocoelacanthus'

 'Latimeria'

 'Laugia'

 'Libys'

 'Lochmocercus'

 'Lualabaea'

 'Luopingcoelacanthus'

 'Macropoma'

 'Mawsonia'

 'Megalocoelacanthus'

 'Miguashaia'

 'Ngamugawi'

 'Parnaibaia'

 'Piveteauia'

 'Polyosteorhynchus'

 'Rebellatrix'

 'Reidus'

 'Rhabdoderma'

 'Rieppelia'

 'Sassenia'

 'Serenichthys'

 'Spermatodus'

 'Swenzia'

 'Ticinepomis'

 'Trachymetopon'

 'Undina'

 'Whiteia'

 'Wimania'

 'Yunnancoelacanthus'


 ;

ENDBLOCK;


BEGIN CHARACTERS;

 DIMENSIONS NCHAR=112;

 FORMAT DATATYPE=STANDARD MISSING=? GAP=- SYMBOLS="012345";

 CHARSTATELABELS


 1 Parietonasal_and_postparietal_shields_ /

 free_from_one_to_another_

 sutured_to_each_other_,


 2 Parietonasal_versus_postparietal_shield_ /

 smaller_or_almost_of_the_same_length_

 longer_,


 3 Snout_bones /

 lying_free_from_one_another_

 consolidated_,


 4 Premaxillary_teeth_ /

 equal_or_more_than_5_

 equal_or_less_than_4_,


 5 Premaxilla_ /

 with_dorsal_lamina_

 without_dorsal_lamina_,


 6 Anterior_opening_of_the_rostral_organ_ /

 contained_within_premaxilla_

 within_separated_rostral_ossicle_,


 7 Internasal_ /

 several_

 one_or_none_,


 8 parietal_ /

 one_pair_

 two_pairs_,


 9 Anterior_and_posterior_pairs_of_parietals_ /

 of_similar_size_

 of_dissimilar_size_,


 10 Parietals_and_postparietals_ /

 without_raised_areas_

 with_raised_areas_,


 11 Parietal_descending_process_ /

 absent_or_highly_reduced_to_a_ridge_

 present_,


 12 'Number of supraorbitals/tectals' /

 equal_or_less_than 9_

 equal_or_more_than_10_,


 13 Preorbital_ /

 absent_

 present_,


 14 Intertemporal_ /

 absent_

 present_,


 15 Postparietal_descending_process_ /

 absent_or_highly_reduced_to_a_ridge_

 present_,


 16 Supratemporal_descending_process_ /

 absent_or_highly_reduced_to_a_ridge_

 present_,


 17 Posterior_margin_of_the_skull_roof_ /

 straight_

 embayed_,


 18 Extrascapulars_ /

 sutured_with_postparietals_

 free_,


 19 Extrascapulars_ /

 behind_level_of_neurocranium_

 forming_part_of_the_skull_roof_,


 20 'Pair(s)_of_lateral_extrascapulars_(without_the_triple_junction_for_sensory_canals)' /

 none_

 one_

 two_or_more_,


 21 Median_extrascapular_ /

 present_

 absent_,


 22 Supraorbital_sensory_canal_ /

 running_through_centre_of_ossification_

 following_sutural_course_,


 23 Supraorbital_sensory_canals_opening_as_ /

 few_pores_at_the_sutural_contact_of_bones_

 bifurcating_pores_

 many_pores_within_bones_

 continuous_groove_crossed_by_pillars_

 continuous_groove_without_pillars_,


 24 Medial_branch_of_otic_canal /

 absent_

 present_,


 25 Anterior_branches_of_supratemporal_commissure_ /

 absent_

 present_,


 26 Pit_lines_ /

 marking_postparietals_

 not_marking_postparietals__,


 27 Middle_and_posterior_pit_lines_ /

 within_posterior_half_or_in_the_middle_of_postparietals_

 within_anterior_third_,


 28 Dermal_bones_of_the_skull_ornamented_with_ /

 'coarse and/or irregularly shaped tubercles and/or elongated continuous/discontinous vermiform/linear ridges tuberculation '

 round_tubercles_

 coarse_rugosities_and_fine_to_pronounced_striae_

 mostly_or_entirely_unornamented_,


 29 Cheek_bones_ /

 sutured_to_one_another_

 separated_from_one_another,


 30 'Spiracular (postspiracular)' /

 absent_

 present_,


 31 Postorbital_ /

 'simple, without anterodorsal excavation '

 anterodorsal_excavation_in_the_postorbital_,


 32 Postorbital_ /

 without_anterior_process_

 with_anterior_process_,


 33 Postorbital_ /

 large_

 reduced_to_a_narrow_tube_surrounding_the_sensory_canal_only_,


 34 Postorbital_ /

 entirely_behind_the_level_of_the_intracranial_joint_

 spanning_the_intracranial_joint_,


 35 Jugal_ /

 present_

 absent_,


 36 Squamosal_ /

 large_

 reduced_to_a_narrow_tube_surrounding_the_jugal_sensory_canal_only,


 37 Squamosal_ /

 'limited to the mid-level of cheek'

 extending_behind_the_postorbital_to_reach_the_skull_roof_,


 38 Preopercle_ /

 large_

 reduced_to_a_narrow tube_surrounding_the_preopercular_canal_only_,


 39 Preopercle_ /

 undifferentiated_

 'developed as a posterior tube-like canal-bearing portion and an anterior blade-like portion ',


 40 Position_of_the_preopercle_within_the_cheek_ /

 'posterior to the squamosal and/or the postorbital'

 below_or_anterior_to_the_squamosal_and_the_postorbital_,


 41 Subopercle_ /

 absent_

 present_,


 42 Lachrymojugal_ /

 simple_

 'angled and/or expanded',


 43 Lachrymojugal_ /

 with_parallel_margins_along_its_entire_lenght_

 with_a_more_or_less_thick_triangular_portion_,


 44 'contact between the lachrymojugal and the preorbital or tectal-supraorbital series ' /

 present_

 absent_,


 45 Posterior_nostril_on_the_lachrymojugal_ /

 not_marked_

 marked_,


 46 Posterior_opening_of_the_rostral_organ_marks_ /

 the preorbital_

 the lachrymojugal_

 'the tectal and/or no bones',


 47 'Posterior opening(s) of the rostral organ mark(s) bone as' /

 'foramen(s)'

 'notch(es) or groove(s)'

 not_marking_bone_,


 48 'Anterior and/or posterior branches of the infraorbital canal within the postorbital' /

 'absent (canal simple)'

 present_,


 49 Infraorbital_sensory_canal /

 running_through_centre_of_postorbital_

 running_at_the_anterior_margin_of_the_postorbital_,


 50 Prominent_branches_of_the_jugal_sensory_canal_within_the_squamosal_ /

 'absent (canal simple)'

 present_,


 51 Jugal_sensory_canal_ /

 running_through_centre_of_the_squamosal_

 running_along_the_ventral_margin_of_the_squamosal_,


 52 'Infraorbital, jugal and preopercular sensory canals ' /

 opening_through_many_tiny_pores_

 opening_through_a_few_large_pores_

 'opening as a large, continuous groove crossed by pillars',


 53 Pit_lines_ /

 marking_cheek_bones_

 failing_to_mark_cheek_bones_,


 54 Dermal_bones_of_the_cheek_ornamented_with_ /

 'coarse and/or irregularly shaped tubercles and/or elongated continuous/discontinous vermiform/linear ridges tuberculation '

 round_tubercles_

 coarse_rugosities_and_fine_to_pronounced_striae_

 mostly_or_entirely_unornamented_,


 55 Orbital_space_ /

 small_and_occupied_entirely_by_the_eye_

 large_and_not_entirely_occupied_by_the_eye_,


 56 Sclerotic_ossicles_ /

 absent_

 present_,


 57 Retroarticular_and_articular_ /

 'co-ossified'

 separated_,


 58 Dentary_ /

 simple_

 'dentary hook-shaped ',


 59 Dentary_ /

 without_prominent_lateral_swelling_

 with_swelling_,


 60 Dentary_teeth_ /

 with_fused_to_the_dentary_

 with_separated_from_dentary_or_edentulous_,


 61 Principal_coronoid_ /

 lying_free_

 sutured_to_angular_,


 62 Number_of_anterior_coronoids /

 four_or_more_

 three_or_less_,


 63 Coronoid_ /

 opposite_to_the_posterior_end_of_dentary_not_modified_

 modified_,


 64 Coronoid_fangs_ /

 absent_

 present_,


 65 'Prearticular and/or coronoid teeth' /

 pointed_and_smooth_

 rounded_and_marked_with_fine_striations_radiating_from_the_crown_,


 66 Subopercular_branch_of_the_mandibular_sensory_canal_ /

 absent_

 present_,


 67 Dentary_sensory_pore_ /

 absent_

 present_,


 68 Mandibular_sensory_canal_on_the_splenial_ /

 opening_through_laterally_directed_pores_

 opening_through_ventrally_directed_pores_,


 69 Oral_pit_line_ /

 marking_the_angular_

 not_marking_the_angular_,


 70 Oral_pit_line_ /

 confined_to_angular_

 'oral pit line reaching forward to the dentary and/or the splenial ',


 71 Orbitosphenoid_and_basisphenoid_regions_ /

 'co-ossified'

 separate_,


 72 Processus_connectens_ /

 failing_to_meet_parasphenoid_

 meeting_parasphenoid_,


 73 Basipterygoid_process_ /

 absent_

 present_,


 74 Temporal_excavation_ /

 not_lined_with_bone_

 lined_with_bone_,


 75 'Otico-occipital ' /

 solid_

 'separated to prootic/opisthotic',


 76 Supraoccipital_ /

 absent_

 present_,

 77 Toothed_area_of_the_parasphenoid_ /

 covers_most_of_the_ventral_surface_

 restricted_to_the_anterior_half_,


 78 Buccohypophysial_canal_ /

 closed_

 opening_through_parasphenoid_,


 79 Parasphenoid_ /

 without_ascending_laminae_anteriorly_

 with_ascending_laminae_,


 80 Suprapterygoid_process_ /

 absent_

 present_,


 81 Vomers_ /

 not_meeting_in_the_midline_

 meeting_medially_,


 82 Prootic_ /

 without_complex_suture_with_the_basioccipital_

 with_a_complex_suture_,


 83 Superficial_ophthalmic_branch_of_anterodorsal_lateral_line_nerve_ /

 not_piercing_antotic_process_

 piercing_antotic_process_,


 84 Process_on_braincase_for_articulation_of_infrabranchial_1_ /

 absent_

 present_,


 85 Separate_lateral_ethmoids_ /

 absent_

 present_,


 86 Separate_basioccipital_ /

 absent_

 present_,


 87 Dorsum_sellae_ /

 small_

 large_and_constricting_entrance_to_cranial_cavity_anterior_to_the_intracranial_joint_,


 88 Ventral_swelling_of_the_palatoquadrate_ /

 absent_

 present_,


 89 Basibranchial_tooth_plates_ /

 three_median_pairs_or_more_

 two_median_pairs_or_less_,


 90 Anterior_basibranchial_tooth_plates_ /

 paired_

 fused_,


 91 Extracleithrum_ /

 absent_

 present_,


 92 Anocleithrum_ /

 simple_

 forked_,


 93 Number_of_neural_arches_ /

 equal_or_more_than_50_

 equal_or_less_than_49_,


 94 Posterior_neural_and_haemal_spines_ /

 abutting_one_another_

 not_abutting_,


 95 Occipital_neural_arches_ /

 not_expanded_

 expanded_,


 96 Ossified_ribs_ /

 absent_

 present_,


 97 Ossified_lung_ /

 absent_

 present_,


 98 Basal_plate_of_anterior_dorsal_fin_ /

 with_smooth_ventral_margin_

 emarginated_and_accommodating_the_tips_of_adjacent_neural_spines_,


 99 Fin_rays_in_the_anterior_dorsal_fin_ /

 more_or_egual_to_11_

 less_than_or_egual_as_10_,


 100 _Anterior_dorsal_fin_ /

 without_denticles_

 with_denticles_,


 101 _Basal_support_of_the_second_dorsal_fin_ /

 simple_

 forked_anteriorly_,


 102 Pelvics_ /

 abdominal_

 thoracic_,


 103 Pelvic_bones_of_each_side_ /

 remain_separate_

 fused_in_midline,


 104 Diphycercal_tail_ /

 absent_

 present_,


 105 Caudal_lobes_ /

 symmetrical_

 asymmetrical_,


 106 Fin_rays_ /

 more_numerous_than_radials_

 equal_in_number_,


 107 Fin_ray_ /

 branched_

 unbranched_,


 108 Paired_fin_rays_ /

 slender_

 expanded_,


 109 Median_fin_rays_ /

 slender_

 expanded_,


 110 Lateral_line_openings_in_scales_ /

 single_

 multiple_,


 111 Ventral_keel_scales_ /

 absent_

 present_,


 112 Scale_ornament_ /

 not_differentiated_

 differentiated_,


;


 MATRIX

 'Onychodontiformes (Onychodus) ' 0 0 0 0 0 - 0 0 - 0 0 0 1 1 0 0 0 0 0 0 0 0 2 0 0 0 0 0 0 1 0 0 0 0 0 0 0 0 0 0 1 - - - - - - 0 1 0 0 0 0 0 0 1 - 0 0 0 - 0 0 1 0 0 0 - - - 0 - 1 0 ? ? 0 1 0 1 - 0 - ? 1 0 0 ? ? ? 0 0 ? ? ? ? ? 1 0 0 ? ? ? 1 ? 0 0 ? ? 1 ? 0

 'Allenypterus' 0 1 ? 0 ? ? 1 1 0 0 ? 0 1 0 ? ? 0 0 0 0 0 1 0 0 0 0 0 0 1 ? 0 0 1 0 1 0 0 0 0 1 1 0 0 0 0 0 0 0 ? ? ? 1 1 3 0 1 0 0 0 1 0 ? ? ? ? 0 0 0 0 1 ? ? ? ? ? ? ? ? ? ? ? ? ? ? ? ? ? 0 ? ? 1 0 0 1 0 0 1 0 0 0 0 0 0 1 1 0 1 0 0 0 1 0

 'Atacamaia' 0 ? ? ? ? ? ? 0 - 1 ? ? 1 0 ? ? 1 1 0 ? ? ? 2 1 ? 0 1 0 ? ? 0 0 1 1 ? ? ? ? ? ? 0 1 0 0 ? ? ? ? 0 ? ? ? ? 0 0 1 ? ? ? ? ? ? ? ? ? ? ? ? ? ? ? ? ? ? ? ? ? ? ? ? ? ? ? ? ? ? ? 0 ? ? ? ? ? ? ? ? ? ? ? ? ? ? ? ? ? ? ? ? ? ? 0 ?

 'Axelia' 0 0 ? ? ? ? 1 0 ? 0 1 0 1 0 ? 1 1 1 0 2 1 1 0 ? 0 ? ? ? ? ? ? ? ? ? ? ? ? ? ? ? ? ? ? ? ? 0 ? ? ? ? ? ? ? 0 ? 1 ? 0 ? ? 0 ? ? 0 1 ? 1 ? ? ? ? ? ? ? ? ? 0 ? ? ? ? ? ? ? ? ? ? 0 ? ? ? ? ? ? ? ? ? ? ? 1 ? ? ? ? ? ? ? 0 ? ? ? 0

 'Axelrodichthys' 0 1 ? 1 1 0 1 1 1 0 1 0 0 0 1 0 1 0 1 1 0 1 2 0 0 1 ? 2 1 0 0 1 0 1 1 0 0 0 0 1 0 1 0 0 ? 1 1 ? 0 0 0 0 1 2 1 0 1 1 1 1 1 0 0 0 1 0 1 1 1 ? 1 0 0 1 1 1 0 0 0 0 1 1 0 ? 1 1 1 0 1 0 1 0 ? 1 1 1 1 0 {01} 1 1 0 0 1 0 1 1 0 0 ? 0 1

 'Caridosuctor' 0 0 0 {01} 0 0 1 1 1 0 ? 1 1 0 ? ? 0 0 0 1 0 1 0 1 0 0 1 0 0 1 0 0 0 0 1 0 ? 0 0 0 1 0 0 0 ? 0 0 ? ? 0 0 0 0 0 0 ? 0 0 0 1 0 0 1 1 ? 0 1 0 0 0 ? ? ? ? ? ? ? ? ? ? ? ? ? ? ? ? ? ? ? ? 1 0 0 1 ? 0 1 1 0 0 1 0 0 1 1 1 1 0 0 ? 0 0

 'Chinlea' 0 1 ? 0 1 ? 1 1 {01} 0 1 {01} 0 0 ? ? 1 0 1 2 {01} ? ? 1 0 ? ? 2 1 ? 0 0 0 1 1 0 ? 0 0 1 0 1 0 0 0 1 1 ? 0 ? ? 0 ? 2 1 0 ? 1 0 ? 0 ? 1 1 ? ? 1 ? 1 ? ? ? ? ? ? ? ? ? ? ? ? ? ? ? ? ? 1 ? ? ? 1 ? ? 1 ? 1 ? ? 1 0 1 0 0 1 ? 1 1 0 0 ? 0 1

 'Coccoderma' 0 0 ? ? 1 1 ? 1 1 0 1 0 1 0 0 1 0 0 0 1 0 1 2 1 0 0 1 3 1 0 0 0 0 0 1 1 ? 1 0 0 0 0 0 0 0 0 ? 1 0 0 1 0 1 3 0 1 ? 0 0 1 0 0 1 0 ? 0 1 0 0 0 ? ? ? ? ? ? ? ? ? ? ? ? ? ? ? ? ? 0 ? ? 1 1 0 1 1 0 1 0 {01} 0 1 1 1 1 0 1 1 1 0 1 0 0

 'Coelacanthus' 0 0 0 ? 1 ? ? 1 1 0 1 0 0 0 0 1 1 0 0 1 0 ? ? 1 0 1 ? 3 1 ? 0 0 1 0 1 1 0 ? ? ? ? ? 0 ? ? ? ? ? 0 ? 0 0 ? 0 ? 1 0 0 0 1 0 0 1 0 0 0 1 0 1 ? ? ? ? ? ? ? ? ? ? ? ? ? ? ? ? ? ? 0 ? ? 1 ? 0 1 0 0 1 0 0 0 1 0 0 1 0 1 1 0 0 ? 0 0

 'Diplocercides' 0 0 0 ? ? ? 0 1 1 0 0 1 1 0 0 0 0 0 0 0 0 1 2 0 0 0 0 0 0 1 0 0 0 0 1 0 1 0 0 1 1 0 {01} 0 ? 0 0 0 0 0 0 0 0 0 0 1 0 0 0 0 0 1 0 0 0 0 0 0 0 1 0 1 1 1 0 0 0 1 0 1 ? 0 1 0 0 0 0 0 ? ? ? ? ? 0 0 0 ? ? 0 0 ? 0 ? 1 0 0 1 0 0 0 ? 0

 'Diplurus' 0 1 0 0 1 1 1 1 1 0 1 0 0 0 1 0 1 1 0 2 ? 1 0 1 0 1 ? 3 1 1 0 0 1 0 1 1 0 1 0 1 0 1 0 0 0 {12} 1 0 0 ? ? 1 1 3 0 0 1 ? 1 ? 0 ? 0 0 0 0 1 1 ? ? 1 0 0 ? 1 1 0 0 0 ? ? ? 0 ? 1 1 1 0 1 0 1 0 {01} 1 1 1 0 0 {01} 1 1 0 0 1 0 1 1 0 0 ? 0 1

 'Dobrogeria' 0 ? ? ? ? ? ? ? ? 0 1 ? ? 0 1 1 1 1 0 ? ? 1 ? 1 ? 1 ? 0 1 ? ? ? ? ? ? ? ? 0 0 ? 1 1 0 ? ? ? ? ? ? ? ? ? ? 0 ? ? 1 ? ? ? ? ? ? ? ? ? 1 0 0 0 1 1 0 0 1 ? ? ? ? ? ? ? 0 ? ? 1 1 0 ? ? 1 ? ? ? ? ? ? ? ? ? ? ? ? ? ? ? ? ? ? ? ? ?

 'Euporosteus' 0 0 ? ? ? ? 0 1 1 ? 0 ? 1 ? ? ? 0 ? ? ? 0 ? 0 ? ? ? ? ? ? ? ? ? ? ? ? ? ? ? ? ? ? ? ? ? ? ? ? ? ? ? ? ? ? ? ? ? ? ? ? ? ? ? ? ? ? ? ? ? ? ? 0 1 ? ? ? ? 0 1 0 1 0 ? 1 ? 0 ? 0 ? ? ? ? ? ? ? ? ? ? ? ? ? ? ? ? ? ? ? ? ? ? ? ? ?

 'Foreyia' 1 0 0 ? 1 1 1 1 0 0 ? 0 1 0 ? ? ? ? ? ? ? 1 4 ? ? ? ? 1 1 0 1 0 1 0 1 ? 0 ? 0 1 1 1 1 0 0 0 1 0 0 ? 0 0 1 1 0 0 ? 1 0 1 0 0 0 1 0 ? 1 0 ? ? ? 0 ? ? ? ? ? ? 1 ? ? ? ? ? 1 ? ? 1 ? ? 1 0 1 1 ? 0 0 ? 0 1 ? 0 ? 1 0 1 1 0 0 ? ? 1

 'Garnbergia' 0 1 ? ? ? ? ? 1 ? ? ? 0 0 0 ? ? 1 ? ? ? ? ? ? ? ? ? ? 3 1 ? 0 ? 0 0 1 0 0 ? ? ? 0 ? 0 0 ? ? ? ? ? ? ? ? ? 3 0 0 ? ? ? ? ? ? ? ? ? ? ? ? ? ? ? ? ? ? ? ? ? ? ? ? ? ? ? ? ? ? ? ? ? ? ? ? ? ? ? ? ? 0 ? 0 1 ? ? ? ? ? ? ? 0 ? 0 0

 'Gavinia' ? ? 0 0 0 0 ? ? ? ? ? ? ? 1 ? ? ? ? ? ? ? 0 ? ? ? ? ? 0 0 ? 0 0 0 ? ? 0 1 ? ? 0 ? ? ? ? ? ? ? 0 0 0 0 0 0 0 0 ? 0 0 0 0 ? ? ? ? ? ? 0 ? ? ? ? ? ? ? ? ? ? ? ? ? ? ? ? ? ? ? ? ? ? ? ? ? ? ? ? ? ? ? ? ? ? ? ? 0 1 1 0 ? 0 ? ? 0

 'Guizhoucoelacanthus' 0 1 0 ? ? ? 1 1 0 0 ? 0 1 0 ? ? 1 0 0 1 0 1 2 1 0 0 0 3 1 ? 0 0 0 0 1 0 0 ? 0 1 ? 1 0 0 ? 0 1 ? 0 ? 0 0 ? 3 0 0 ? 0 0 ? 0 ? ? 0 ? ? 1 ? ? 0 ? ? ? ? ? ? ? ? ? ? ? ? ? ? ? ? ? ? ? ? 1 ? 1 1 0 0 ? 0 1 0 1 0 ? 1 0 1 1 0 0 0 0 0

 'Hadronector' 0 1 0 0 0 0 0 1 1 0 ? ? 1 0 ? ? 0 0 0 0 0 1 1 0 0 0 1 0 0 1 0 0 0 0 1 0 0 0 0 1 1 0 0 0 ? 0 0 ? 1 ? 0 0 ? 0 0 1 0 0 0 ? 0 ? ? ? ? 0 0 0 0 0 ? ? ? ? ? ? ? ? ? ? ? ? ? ? ? ? ? 1 ? ? 1 0 1 1 1 0 1 0 1 0 1 0 0 1 0 1 1 0 0 ? 0 ?

 'Heptanema' 0 1 0 ? ? ? ? 1 0 0 ? ? ? ? ? ? ? ? ? ? ? ? ? ? ? ? ? 3 1 ? ? ? ? ? 1 ? 0 ? 0 ? ? 1 0 ? ? ? ? ? ? ? ? ? ? 3 0 0 ? 1 ? ? ? ? ? ? ? ? ? ? ? ? ? ? ? ? ? ? ? ? ? ? ? ? ? ? ? ? ? ? ? ? 1 0 ? 1 ? 1 1 ? 1 1 1 0 ? 1 0 1 1 0 0 ? ? 1

 'Holophagus' 0 1 0 ? 1 ? 1 1 1 0 1 1 0 0 1 1 1 1 0 ? ? 1 2 1 ? 1 ? 3 1 0 0 0 0 0 1 0 0 0 0 1 1 1 0 0 ? ? {12} ? 1 ? ? 0 1 1 0 0 1 1 0 ? 0 ? 1 1 ? 1 1 0 0 0 ? ? ? ? 1 1 ? ? ? ? ? 1 ? ? 1 1 ? ? ? ? 1 ? 0 1 1 0 1 0 {01} 1 1 0 0 1 ? 1 1 1 1 ? 0 0

 'Holopterygius' ? ? 0 ? ? ? ? ? ? ? ? ? ? ? ? ? ? ? ? ? ? ? 0 ? ? ? ? ? ? ? ? ? ? ? ? ? ? ? ? ? ? ? ? ? ? ? ? ? ? ? ? ? ? ? ? ? 0 0 ? 0 ? ? ? 0 0 ? ? ? ? ? ? ? ? ? ? ? ? 0 0 ? ? ? ? ? ? ? ? ? ? ? 1 0 0 ? 0 0 0 ? ? ? 0 ? ? 1 1 0 1 ? 0 ? 1 ?

 'Indocoelacanthus' 0 ? ? ? ? ? ? 1 ? 0 ? ? ? 0 ? ? ? ? ? ? ? 1 2 ? ? ? ? 2 1 1 0 ? 0 ? 1 0 0 0 0 1 ? ? 0 ? ? ? ? ? ? ? ? 0 ? 2 ? ? ? 1 1 1 0 ? ? ? ? ? ? ? ? ? ? ? ? ? ? ? ? ? ? ? ? ? ? ? ? ? ? 0 ? ? ? ? ? ? ? 1 ? 0 ? ? ? ? 0 ? ? ? 1 ? ? ? ? 0

 'Latimeria' 0 1 0 1 1 1 1 1 1 1 1 1 0 0 1 1 1 1 0 2 0 1 0 1 1 1 1 3 1 1 1 0 0 0 1 0 0 0 1 1 1 1 0 1 1 2 2 1 1 1 1 1 1 1 0 0 1 1 0 1 0 0 1 1 0 1 1 0 0 0 1 0 0 0 1 1 1 0 1 0 1 1 0 0 1 1 1 1 1 0 1 0 0 1 1 0 0 0 1 1 1 0 0 1 0 1 1 0 0 1 0 0

 'Laugia' 0 0 1 0 ? ? ? 0 ? 0 1 0 1 0 0 1 0 0 0 1 0 1 2 1 0 1 ? 0 1 0 0 0 0 0 1 0 0 ? ? ? 0 0 0 0 0 0 1 1 0 0 1 0 1 0 0 1 0 0 0 1 0 0 0 0 0 0 1 0 0 0 1 1 0 1 1 0 0 1 0 ? ? 0 0 0 1 1 1 0 0 0 1 0 0 1 1 0 1 0 1 0 1 1 1 1 1 1 1 1 0 ? 0 0

 'Libys' 0 1 1 ? {01} ? 1 1 ? 0 1 ? 0 0 1 1 1 1 0 ? ? 1 3 1 ? 1 ? 3 1 0 0 0 0 0 1 0 0 0 1 1 0 1 0 1 ? ? ? ? 1 ? 1 2 ? 3 0 1 ? 1 0 1 0 0 1 0 1 1 1 ? 0 0 1 ? ? ? ? ? 1 ? 1 ? ? ? ? ? ? ? ? 1 ? ? 1 0 {01} 1 1 0 1 0 1 1 1 0 0 1 0 1 1 {01} {01} 1 0 {01}

 'Lochmocercus' 0 ? ? ? ? ? ? ? ? 0 ? ? 1 ? ? ? ? ? ? ? ? 1 0 0 0 ? ? ? 0 1 0 0 0 ? ? 0 ? 0 0 ? 1 ? 0 0 ? 0 0 0 1 0 0 0 0 ? 0 1 ? 0 0 0 0 ? 0 0 ? 0 0 ? ? ? ? ? ? ? ? ? ? ? ? ? ? ? ? ? ? ? ? 0 ? ? 1 0 ? 1 ? ? ? 1 0 0 0 0 0 1 0 0 1 0 0 ? 0 ?

 'Lualabaea' 0 1 ? ? ? ? ? ? ? 0 ? ? ? 0 ? ? ? 0 1 ? 0 ? ? ? ? ? ? 2 ? ? ? ? ? ? ? ? ? ? ? ? ? 1 0 ? ? 1 1 ? ? ? ? ? ? 2 0 ? ? 1 1 1 1 ? ? ? ? ? ? ? ? ? ? ? ? ? ? ? ? ? ? ? ? ? ? ? ? ? ? 0 ? ? ? ? ? ? ? ? ? ? 0 1 ? ? ? ? ? ? ? ? ? ? ? 1

 'Luopingcoelacanthus' 0 ? ? 1 1 ? ? 1 1 0 ? 0 ? 0 1 ? 1 1 0 ? ? ? ? ? ? 1 ? ? 1 ? 0 0 0 ? ? 0 0 0 0 ? 0 ? 1 ? ? ? ? ? ? 0 0 0 1 ? 0 1 1 1 0 1 0 ? ? 0 ? ? 1 ? ? ? 1 0 ? ? ? ? 0 ? 1 ? ? ? ? ? ? 1 ? ? ? ? 1 0 ? 1 0 0 1 0 0 1 1 0 0 1 0 1 1 0 0 ? 0 1

 'Macropoma' 0 1 1 1 ? 1 1 1 1 0 1 1 0 0 1 1 1 1 0 2 0 1 {02} 1 1 1 ? 1 1 0 1 0 0 0 1 0 0 0 1 1 ? 1 0 1 1 1 1 1 1 0 1 0 1 1 0 0 1 1 0 1 0 0 ? 0 0 1 1 0 0 0 1 0 0 0 1 1 1 0 1 ? 1 1 0 ? 1 1 1 1 ? 1 1 1 0 1 1 0 1 0 1 1 1 0 0 1 0 1 1 0 0 1 0 1

 'Mawsonia' 0 1 ? ? ? ? 1 1 1 0 1 0 0 0 1 0 1 0 1 1 1 1 2 1 0 1 ? 2 1 0 0 1 0 1 1 0 0 0 0 1 0 1 0 1 0 1 1 ? 0 0 0 0 1 2 1 0 1 1 1 1 {01} ? ? 0 {01} ? 1 1 1 ? ? 1 0 ? 1 1 0 ? ? ? ? ? 0 ? ? ? 1 0 ? ? 1 ? 0 1 ? 1 1 {01} 1 {01} 1 0 0 1 0 1 1 0 0 ? ? 1

 'Megalocoelacanthus' 0 1 1 ? 0 1 1 1 1 0 1 ? ? 0 1 1 1 1 0 ? ? 1 3 1 ? ? ? 3 1 ? ? ? ? ? ? ? ? ? ? ? ? ? ? ? ? ? ? ? ? ? ? 2 ? 3 ? ? 1 1 0 1 0 ? 0 0 1 1 1 0 1 ? 1 0 0 1 1 ? 1 0 1 ? ? ? 0 ? 1 ? 1 1 1 1 1 ? ? ? 1 ? ? ? ? ? ? ? ? ? ? ? ? ? ? ? ? ?

 'Miguashaia' 0 0 0 0 0 0 ? 0 ? 0 0 0 ? 1 ? ? 0 0 0 0 0 0 2 0 0 0 0 0 0 ? 0 0 0 0 0 0 1 0 0 0 1 ? 0 ? ? ? ? 0 0 0 0 0 ? 0 0 1 ? 0 0 0 0 ? ? 0 0 ? ? 0 {01} 0 ? ? ? ? ? ? ? ? ? ? ? ? ? ? ? ? ? ? ? ? 1 ? ? 0 ? ? ? ? 0 0 ? 0 ? 0 1 0 0 0 0 0 0 0

 'Ngamugawi' 0 0 0 0 0 0 0 1 1 0 0 ? ? 0 0 0 0 0 0 ? 0 1 0 1 ? 0 0 0 0 0 0 0 0 0 1 0 1 0 0 1 1 0 1 ? ? 0 0 0 0 0 0 0 0 0 0 ? 0 0 0 1 0 1 0 0 1 0 0 0 0 1 0 0 1 ? 0 0 0 1 0 1 ? ? 0 0 ? 0 0 0 ? ? 1 0 ? ? 0 ? ? ? ? ? ? ? ? ? ? ? ? ? ? 0 ? 0

 'Parnaibaia' 0 1 0 0 ? ? 1 1 0 0 ? 0 0 0 ? ? ? 0 ? 2 1 ? 2 1 ? 0 0 2 1 0 0 0 0 ? 1 0 0 ? ? ? ? 1 0 0 0 1 1 ? ? ? ? ? ? 2 1 0 1 1 1 ? ? ? 0 0 0 ? 1 1 ? ? ? ? ? ? ? ? ? 1 ? ? ? ? ? ? ? 1 ? ? ? 0 1 ? ? 1 ? 1 1 0 1 1 ? 0 0 1 0 1 1 0 0 ? ? 1

 'Piveteauia' 0 0 ? ? ? ? ? ? ? 0 1 ? ? 0 1 1 1 ? 0 ? ? ? ? 1 ? ? ? 0 ? 1 ? ? ? ? ? 0 0 ? ? 0 ? 0 ? ? 0 ? ? ? 0 1 0 ? ? 0 0 1 1 0 0 1 0 ? ? ? 0 ? 1 ? ? ? ? ? ? ? ? ? 0 1 1 ? ? ? ? ? ? ? ? ? ? ? 1 ? 0 ? 1 0 1 0 1 0 1 1 ? 1 1 1 1 0 0 ? 0 0

 'Polyosteorhynchus' 0 1 0 1 0 0 ? 1 1 0 ? 0 1 ? ? 1 0 0 0 0 0 1 1 0 0 ? ? 0 0 1 0 0 0 0 1 0 0 0 0 1 1 0 0 0 ? 0 0 ? 1 ? 0 0 0 0 0 1 0 0 0 1 0 ? 0 0 ? 0 1 0 0 0 ? ? ? ? ? ? ? ? ? ? ? ? ? ? ? ? ? 0 ? ? 1 ? 0 1 1 0 1 1 1 0 0 0 0 1 0 1 1 0 0 ? 0 0

 'Rebellatrix' ? ? ? ? ? ? ? ? ? ? ? ? ? ? ? ? ? ? ? ? ? ? ? ? ? ? ? ? ? ? ? ? ? ? ? ? ? ? ? ? ? ? ? ? ? ? ? ? ? ? ? ? ? ? ? ? 1 ? ? ? ? ? ? ? ? ? ? ? 0 ? ? ? ? ? ? ? ? ? ? ? ? ? ? ? ? ? ? ? ? ? 1 0 0 1 1 0 ? 0 0 0 1 0 0 1 0 1 1 0 0 0 ? 0

 'Reidus' ? ? ? ? ? ? ? ? ? ? ? ? ? ? ? ? ? ? ? ? ? ? ? ? ? ? ? ? 1 ? ? ? ? ? ? 0 0 0 0 ? ? ? ? ? ? ? ? ? ? ? ? ? 1 3 ? ? 1 1 1 1 0 0 0 0 0 ? 1 1 0 0 ? ? ? ? ? ? ? ? ? ? ? ? ? ? ? ? ? ? ? ? ? ? ? ? ? ? ? ? ? ? ? ? ? ? ? ? ? ? ? ? ? ?

 'Rhabdoderma' 0 0 0 1 0 0 1 1 0 0 1 0 1 0 0 1 0 0 0 1 0 1 0 1 0 0 1 0 0 1 0 0 0 0 1 0 0 0 0 0 1 0 0 0 0 0 0 0 1 1 0 0 0 0 0 1 0 0 0 1 0 0 1 1 0 0 1 0 0 0 1 1 0 1 0 ? 0 0 0 0 ? 0 0 0 1 0 1 0 ? ? 1 0 {01} 1 0 0 1 1 {01} 0 1 0 0 1 0 1 1 0 0 0 0 0

 'Rieppelia' 1 0 0 ? 0 1 1 1 0 0 1 0 1 0 1 1 0 0 ? 0 0 1 0 0 0 1 ? 1 0 0 0 0 ? 0 1 ? ? 0 0 1 0 ? 1 0 1 2 2 ? ? ? ? 1 1 1 0 0 ? ? ? 1 0 0 0 1 0 ? ? ? ? ? 1 ? 0 ? 1 ? ? 0 1 ? ? ? ? ? ? ? ? ? ? ? 1 0 1 1 ? 0 0 ? 0 1 ? 0 0 1 1 0 1 0 0 ? 0 0

 'Sassenia' 0 0 0 ? ? ? ? 1 ? 0 1 0 1 0 0 1 0 0 0 ? ? 1 2 ? 0 0 1 1 1 1 0 0 0 0 1 0 1 0 0 0 1 0 0 0 0 0 0 0 1 0 0 0 0 1 0 1 ? 0 0 1 0 ? 1 ? ? 0 1 0 1 ? 0 1 0 1 0 0 ? ? 0 1 ? 0 0 1 1 0 1 0 ? ? 1 ? ? ? ? ? ? ? ? ? ? ? ? ? ? ? ? ? ? ? 0 0

 'Serenichthys' 0 0 0 ? ? 0 ? 1 0 0 ? ? 1 0 ? ? 0 0 0 1 0 ? ? ? ? ? ? 0 0 1 0 0 0 0 1 0 0 0 0 1 1 0 1 0 0 ? ? 0 1 ? 0 0 ? 0 0 1 ? 0 ? 0 0 ? ? ? ? ? ? ? ? ? ? ? ? ? ? ? ? ? ? ? ? ? ? ? ? ? ? ? ? ? 1 0 ? 1 ? 0 ? ? 1 0 ? 0 ? 1 0 0 1 0 0 ? 0 0

 'Spermatodus' 0 0 0 0 0 0 1 1 1 0 1 0 1 0 0 1 0 1 0 ? 0 1 2 1 0 1 ? 0 0 ? 0 0 0 ? 1 0 1 0 0 0 1 0 0 0 ? 0 0 ? ? ? 0 0 0 0 0 1 0 ? ? 1 0 0 1 1 1 0 ? ? 0 0 1 1 0 ? ? 0 0 1 0 ? ? 0 0 ? 1 0 1 ? ? ? ? ? ? ? ? ? ? ? ? ? ? ? ? ? ? ? ? ? ? ? ? 0

 'Swenzia' 0 1 1 ? ? ? ? 1 ? 1 ? ? ? 0 1 1 1 ? ? ? ? ? ? ? 1 0 1 3 1 1 1 0 0 0 1 0 0 0 1 1 ? 1 0 ? 1 1 1 1 1 0 1 0 1 1 0 0 ? 1 0 1 ? ? ? ? 0 ? 1 0 0 0 ? ? ? ? ? ? ? ? 1 ? ? ? ? ? ? ? ? ? ? ? ? ? 0 1 ? 0 1 0 ? ? 1 0 ? 1 0 1 ? ? ? ? ? 0

 'Ticinepomis' 0 1 ? 1 ? ? ? 1 0 0 1 ? 1 0 1 1 0 1 ? ? ? ? 2 ? ? ? ? 0 1 ? ? 0 1 ? ? 1 ? 0 0 ? ? 1 1 ? 1 ? ? ? ? ? ? 1 ? 0 0 0 1 {01} 0 1 0 ? ? 1 0 1 1 0 ? ? 1 0 0 0 1 ? 0 0 1 ? ? 1 ? ? 1 1 ? ? ? 0 1 0 1 1 ? 0 0 0 1 1 1 0 0 1 0 1 1 0 0 ? 0 {01}

 'Trachymetopon' 0 1 ? ? ? ? ? ? ? 0 1 ? ? 0 1 0 1 0 1 1 0 ? ? ? 0 1 ? 2 1 ? ? ? 0 ? ? ? ? ? ? ? ? ? 0 ? ? ? ? ? ? ? ? ? ? 2 ? 0 1 1 0 1 1 ? ? 0 ? 0 1 0 ? 0 1 1 0 ? 1 ? ? ? ? 0 ? 0 0 ? 1 ? 1 0 ? ? 1 ? 0 1 1 1 1 0 0 1 1 0 0 1 0 1 1 0 0 1 0 1

 'Undina' 0 1 0 ? 0 ? ? 1 1 1 1 1 0 0 1 1 1 1 0 2 ? 1 2 ? ? ? ? 3 1 0 0 0 0 0 ? 0 0 0 0 ? 1 1 ? 0 ? ? ? ? 1 0 1 0 1 0 0 0 1 1 0 ? 0 ? 1 1 0 ? 1 0 0 0 1 0 0 0 1 1 1 0 1 ? ? 1 ? ? 1 1 1 1 ? 0 1 0 0 1 ? 0 1 0 1 1 1 0 0 1 0 1 1 {01} {01} 1 0 0

 'Whiteia' 0 1 0 {01} 0 0 1 1 0 0 1 0 1 0 0 1 1 1 0 {12} 0 1 2 1 0 0 1 3 1 1 0 0 0 0 1 0 0 0 0 1 1 1 0 0 0 0 0 1 1 1 0 0 0 0 0 1 1 1 0 1 0 0 1 0 0 ? 1 0 0 0 1 0 0 1 1 ? 0 0 0 ? 1 1 0 1 1 1 1 0 0 ? 1 ? 1 1 0 0 {01} 0 1 {01} 1 0 0 1 0 1 1 0 0 1 0 0

 'Wimania' ? ? ? ? ? ? ? 0 ? ? 1 ? ? 0 0 1 ? ? ? ? ? ? ? ? ? ? ? ? 1 ? ? 0 0 ? 1 0 ? 0 0 1 ? 1 0 0 ? ? ? ? ? ? 0 ? ? ? 1 1 ? 0 ? ? ? ? 1 ? 0 ? ? ? ? ? ? ? ? 1 ? ? ? ? ? ? ? ? ? ? ? ? ? 0 ? ? ? ? ? ? ? 0 ? ? ? ? ? ? ? ? ? ? ? ? ? ? ? 0

 'Yunnancoelacanthus' 0 0 ? ? ? ? 0 1 0 0 ? 0 1 0 1 1 1 0 1 1 0 ? ? ? ? 1 0 0 1 ? 0 0 0 ? 1 0 0 0 0 ? 0 ? ? 0 ? ? ? ? ? ? ? ? 1 0 0 ? ? 0 ? 0 0 ? ? 0 0 0 ? ? ? ? ? ? ? ? ? ? ? ? ? ? ? ? ? ? 1 ? ? ? ? ? ? ? ? 1 0 0 ? ? 1 1 1 0 0 1 ? ? 1 0 0 ? 0 1

 ;

ENDBLOCK;


BEGIN ASSUMPTIONS;

 OPTIONS DEFTYPE=UNORD POLYTCOUNT=MINSTEPS;

ENDBLOCK;


BEGIN NOTES;

 [Taxon comments]


 [Character comments]


 [Character state comments]


 [Attribute comments]


 [Taxon pictures]


 [Character pictures]


 [Character state pictures]


 [Attribute pictures]

ENDBLOCK;
