# Supplementary material for: A deep dive into the coelacanth phylogeny
Source: PLoS One. 2025 Jun 6;20(6):e0320214. doi: 10.1371/journal.pone.0320214 (PMC12143573; doi:10.1371/journal.pone.0320214)
Supplement: S2 Data — (PDF) [file pone.0320214.s002.pdf]

Supporting information 2 for:

A deep dive within the coelacanth phylogeny

Christophe Ferrante[1, 2], Lionel Cavin[1, 2*]

[1]Natural history Museum of Geneva, Geneva, Switzerland.

[2]Department of Earth Sciences, University of Geneva, Geneva, Switzerland.

*Corresponding authors

E-mail: lionel.cavin@geneve.ch (LC)

E-mail: paleo-ferrante.ch@bluewin.ch (CF)

Table of contents

SI 2.1 LIST OF CHARACTERS 2

DERMAL BONES OF THE SKULL ROOF 2

CHEEK BONES AND SENSORY CANALS 5

LOWER JAW 9

NEUROCRANIUM, PARASPHENOID AND VOMER 11

PALATE, HYOID AND GILL ARCHES 13

POSTCRANIAL SKELETON 13

SI 2.2 CHARACTERS NOT INCLUDED IN THE PRESENT ANALYSIS 16

REFERENCES 22

S2.3 DATAMATRIX 23

S2.4 LIST OF APOMORPHIES 26

S2.5 LIST OF CHARACTER CHANGES 30

S2.6 CORRESPONDENCE BETWEEN THE OLD AND THE NEW CHARACTERS NUMBERING .. 34

SI 2.1 List of characters

Dermal bones of the skull roof

<u>Character 1</u>

Parietonasal and postparietal shields [Ferrante & Cavin 2023]

0. free from one to another
1. sutured to each other

<u>Character 2</u>

Parietonasal versus postparietal shields [Ferrante & Cavin 2023]

0. parietonasal shield shorter than the postparietal shield, both equal in length, or parietonasal shield only slightly longer the postparietal shield (<1.25)
1. parietonasal shield significantly longer than the postparietal shield (>1.25)

<u>Character 3</u>

Snout bones [character 2 of Forey 1998]

0. lying free from one another
1. consolidated

<u>Character 4</u>

Premaxillary teeth [Ferrante & Cavin 2023]

0. equal or more than 5
1. equal or less than 4

<u>Character 5</u>

Premaxilla [character 5 of Forey 1998]

0. with dorsal lamina
1. without dorsal lamina

<u>Character 6</u>

Anterior opening of the rostral organ [character 6 of Forey 1998]

0. contained within premaxilla
1. within separated rostral ossicles

<u>Character 7</u>

Internasal [Modified definition of character 3 of Forey 1998]

0. several
1. one or none

<u>Character 8</u>

Parietal [[7]: character 7]

0. one pair
1. two pairs

Character 9

Anterior and posterior pairs of parietals [character 8 of Forey 1998]

0. of similar size
1. of dissimilar size

Character 10

Parietals and postparietals [character 28 of Forey 1998]

0. without raised areas
1. with raised areas

Character 11

Posterior parietal descending process [character 11 of Forey 1998]

0. absent
1. present

Character 12

Number of supraorbitals-tectals [Modified definition of character 9 of Forey 1998]

0. equal or less than 9
1. more than 9

Character 13

Preorbital [character 10 of Forey 1998]

0. absent
1. present

Character 14

Intertemporal [character 12 of Forey 1998]

0. absent
1. present

Character 15

Postparietal descending process [Modified definition of character 13 of Forey 1998]

0. absent or highly reduced to a ridge
1. present

Character 16

Supratemporal descending process [Modified definition of character 14 of Forey 1998]

0. absent or highly reduced to a ridge
1. present

Character 17

Posterior margin of the skull roof [character 18 of Forey 1998]

0. straight
1. embayed

Character 18

Extrascapulars [character 15 of Forey 1998]

0. sutured with postparietals
1. free

Character 19

Extrascapulars [character 16 of Forey 1998]

0. behind level of neurocranium
1. forming part of the skull roof

Character 20

Number of paired extrascapulars without the triple junction for sensory canals [Modified definition of character 17 of Forey 1998]

0. none
1. one
2. two or more

Character 21

Median extrascapular [Ferrante & Cavin 2023]

0. present
1. absent

Character 22

Supraorbital sensory canal [character 19 of Forey 1998]

0. running through centre of ossifications
1. following sutural course

Character 23

Supraorbital sensory canals opening as [Modified definition of character 23 of Forey 1998]

0. few pores at the sutural contact of bones
1. bifurcating pores
2. many pores within bones
3. continuous groove crossed by pillars
4. continuous groove without pillars

Character 24

Medial branch of otic canal [character 20 of Forey 1998]

0. absent
1. present

Character 25

Anterior branches of supratemporal commissure [character 22 of Forey 1998]

0. absent
1. present

Character 26

Pit lines [character 26 of Forey 1998]

0. marking postparietals
1. not marking postparietals

Character 27

Middle and posterior pit lines [Modified definition of character 25 of Forey 1998]

0. within posterior half or in the middle of postparietals
1. within anterior third

Character 28

Dermal bones of the skull roof ornamented with [Modified definition of character 27 of Forey 1998]

0. coarse and/or irregularly shaped tubercles and/or elongated continuous/discontinuous vermiform/linear ridged tuberculation
1. round tubercles
2. coarse rugosities and fine to pronounced striae
3. mostly or entirely unornamented

Cheek bones and sensory canals

Character 29

Cheek bones [character 29 of Forey 1998]

0. sutured to one another
1. separated from one another

Character 30

Spiracular (postspiracular) [character 30 of Forey 1998]

0. absent
1. present

Character 31

Postorbital [character 40 of Forey 1998]

0. simple, without anterodorsal excavation
1. anterodorsal excavation in the postorbital

Character 32

Postorbital [character 41 of Forey 1998]

0. without anterior process
1. with anterior process

Character 33

Postorbital [character 42 of Forey 1998]

0. large
1. reduced to a narrow tube surrounding the sensory canal only

Character 34

Postorbital [character 43 of Forey 1998]

0. entirely behind the level of the intracranial joint
1. spanning the intracranial joint

Character 35

Jugal [Ferrante & Cavin 2023]

0. present
1. absent

Character 36

Squamosal [character 37 of Forey 1998]

0. large
1. reduced to a narrow tube surrounding the jugal sensory canal only

Character 37

Squamosal [character 34 of Forey 1998]

0. limited to the mid-level of cheek
1. extending behind the postorbital to reach the skull roof

Character 38

Preopercle [character 38 of Forey 1998]

0. large
1. reduced to a narrow tube surrounding the preopercular canal only

Character 39

Preopercle [character 39 of Forey 1998]

0. undifferentiated
1. developed as a posterior tube-like canal-bearing portion and an anterior blade-like portion

Character 40

Position of the preopercle in the cheek [Ferrante & Cavin 2023]

0. posterior to the squamosal and/or the postorbital
1. below or anterior to the squamosal and the postorbital

Character 41

Subopercle [character 32 of Forey 1998]

0. absent
1. present

Character 42

Anterior end of the lachrymojugal [Ferrante & Cavin 2023; based on characters 35 and 36 of Forey 1998]

0. simple
1. angled and/or expanded

Character 43

Lachrymojugal [Ferrante & Cavin 2023]

0. with parallel margins along its entire length
1. with a thick triangular portion expanded posteroventrally

Character 44

Contact between the lachrymojugal and the preorbital or tectal-supraorbital series [character 51 of Forey 1998]

0. present
1. absent

Character 45

Posterior nostril on the lachrymojugal [Ferrante & Cavin 2023]

0. not marked
1. marked

Character 46

Posterior opening of the rostral organ marks [Ferrante & Cavin 2023]

0. the preorbital
1. the lachrymojugal
2. the tectal and/or no bones

Character 47

Posterior opening(s) of the rostral organ mark(s) bone as [Ferrante & Cavin 2023]

0. foramen(s)
1. notch(es) or groove(s)
2. not marking bone

Character 48

Anterior and/or posterior branches of the infraorbital canal within the postorbital [Modified definition of character 44 of Forey 1998]

0. absent (canal simple)
1. present

Character 49

Infraorbital sensory canal [[7]: character 45]

0. running through centre of postorbital
1. running at the anterior margin of the postorbital

Character 50

Prominent branches of the jugal sensory canal within the squamosal [Modified definition of character 46 of Forey 1998]

0. absent (canal simple)
1. present

Character 51

Jugal sensory canal [character 47 of Forey 1998]

0. running through centre of bone
1. running along the ventral margin of the squamosal

Character 52

Infraorbital, jugal and preopercular sensory canals [character 50 of Dutel et al. 2012]

0. opening through many tiny pores
1. opening through a few large pores
2. opening as a large, continuous groove crossed by pillars

Character 53

Pit lines [character 48 of Forey 1998]

0. marking cheek bones
1. failing to mark cheek bones

Character 54

Dermal bones of the cheek ornamented with [Modified definition of character 49 of Forey 1998]

0. coarse and/or irregularly shaped tubercles and/or elongated continuous/discontinuous vermiform/linear ridged tuberculation
1. round tubercles
2. coarse rugosities and fine to pronounced striae
3. mostly or entirely unornamented

Character 55

Orbital space [Ferrante & Cavin 2023]

0. small and occupied entirely by the eye
1. large and not entirely occupied by the eye

Character 56

Sclerotic ossicles [character 52 of Forey 1998]

0. absent
1. present

Lower jaw

Character 57

Retroarticular and articular [character 53 of Forey 1998]

0. co-ossified
1. separated

Character 58

Dentary [character 57 of Forey 1998]

0. simple
1. dentary hook-shaped

Character 59

Dentary [character 65 of Forey 1998]

0. without prominent lateral swelling
1. with swelling

Character 60

Dentary [Modified definition of character 54 of Forey 1998]

0. with fused dentary teeth
1. with separated dentary teeth or edentulous

Character 61

Principal coronoid [character 66 of Forey 1998]

0. lying free
1. sutured to angular

Character 62

Number of anterior coronoids [Modified definition of character 55 of Forey 1998]

0. four or more
1. three or less

Character 63

Coronoid [character 56 of Forey 1998]

0. opposite to the posterior end of dentary not modified
1. modified

Character 64

Coronoid fangs [character 67 of Forey 1998]

0. absent
1. present

Character 65

Prearticular and/or coronoid teeth [character 68 of Forey 1998]

0. pointed and smooth
1. rounded and marked with fine striations radiating from the crown

Character 66

Subopercular branch of the mandibular sensory canal [character 60 of Forey 1998]

0. absent
1. present

Character 67

Dentary sensory pore [character 61 of Forey 1998]

0. absent
1. present

Character 68

Mandibular sensory canal on the splenial [Ferrante & Cavin 2023]

0. opening through laterally directed pores
1. opening through ventrally directed pores

Character 69

Oral pit line [Ferrante & Cavin 2023]

0. marking the angular
1. not marking the angular

Character 70

Oral pit line [character 58 of Forey 1998]

0. confined to angular
1. oral pit line reaching forward to the dentary and/or the splenial

Neurocranium, parasphenoid and vomer

Character 71

Orbitosphenoid and basisphenoid regions [character 69 of Forey 1998]

0. co-ossified
1. separate

Character 72

Processus connectens [character 71 of Forey 1998]

0. failing to meet parasphenoid
1. meeting parasphenoid

Character 73

Basipterygoid process [character 72 of Forey 1998]

0. absent
1. present

Character 74

Temporal excavation [character 74 of Forey 1998]

0. not lined
1. lined with bone

Character 75

Otico-occipital [character 75 of Forey 1998]

0. solid
1. separated to prootic/opisthotic

Character 76

Supraoccipital [character 76 of Forey 1998]

0. absent
1. present

Character 77

Toothed area of the parasphenoid [Ferrante & Cavin 2023]

0. covers most of the ventral surface (>50%)
1. restricted to the anterior half (=< 50%)

Character 78

Buccohypophysial canal [character 78 of Forey 1998]

0. closed
1. opening through parasphenoid

Character 79

Parasphenoid [character 79 of Forey 1998]

0. without ascending laminae anteriorly
1. with ascending laminae

Character 80

Suprapterygoid process [character 80 of Forey 1998]

0. absent
1. present

Character 81

Vomers [character 81 of Forey 1998]

0. not meeting in the midline
1. meeting medially

Character 82

Prootic [character 82 of Forey 1998]

0. without complex suture with the basioccipital
1. with a complex suture

Character 83

Superficial ophthalmic branch of anterodorsal lateral line nerve [character 83 of Forey 1998]

0. not piercing antotic process
1. piercing antotic process

Character 84

Process on braincase for articulation of infrabranchial 1 [character 84 of Forey 1998]

0. absent
1. present

Character 85

Separate lateral ethmoids [character 85 of Forey 1998]

0. absent
1. present

Character 86

Separate basioccipital [character 86 of Forey 1998]

0. absent
1. present

Character 87

Dorsum sellae [character 87 of Forey 1998]

0. small
1. large and constricting entrance to cranial cavity anterior to the intracranial joint

Palate, Hyoid and gill arches

Character 88

Ventral swelling of the palatoquadrate [character 110 of Dutel et al. 2012]

0. absent
1. present

Character 89

Basibranchial tooth plates [Ferrante & Cavin 2023]

0. three medial pairs or more
1. two medial pairs or less

Character 90

Anterior basibranchial tooth plates [Ferrante & Cavin 2023]

0. paired
1. fused

Postcranial skeleton

Character 91

Extracleithrum [character 88 of Forey 1998]

0. absent
1. present

Character 92

Anocleithrum [character 89 of Forey 1998]

0. simple
1. forked

Character 93

Number of neural arches [Ferrante & Cavin 2023]

0. equal or more than 50
1. equal or less than 49

Character 94

Posterior neural and haemal spines [character 90 of Forey 1998]

0. abutting one another
1. not abutting

Character 95

Occipital neural arches [character 91 of Forey 1998]

0. not expanded
1. expanded

Character 96

Ossified ribs [character 92 of Forey 1998]

0. absent
1. present

Character 97

Ossified lung [character 107 of Forey 1998]

0. absent
1. present

Character 98

Basal plate of the anterior dorsal fin [character 101 of Forey 1998]

0. with smooth ventral margin
1. emarginated and accommodating the tips of adjacent neural spines

Character 99

Fin rays in the anterior dorsal fin (D1) [Modified definition of character 96 of Forey 1998]

0. more or equal to 11
1. less than or equal as 10

Character 100

Anterior dorsal fin [character 98 of Forey 1998]

0. without denticles
1. with denticles

Character 101

Basal support of the second dorsal fin [character 102 of Forey 1998]

0. simple
1. forked anteriorly

Character 102

Pelvic fins [character 100 of Forey 1998]

0. abdominal
1. thoracic

Character 103

Pelvic bones of each side [character 108 of Forey 1998]

0. remain separate
1. fused in midline

Character 104

Diphycercal tail [character 93 of Forey 1998]

0. absent
1. present

Character 105

Caudal lobes [character 97 of Forey 1998]

0. symmetrical
1. asymmetrical

Character 106

Fin rays [character 94 of Forey 1998]

0. more numerous than radials
1. equal in number

Character 107

Fin ray [character 95 of Forey 1998]

0. branched
1. unbranched

Character 108

Paired fin rays [Modified definition of character of character 99 of Forey 1998]

0. slender
1. expanded

Character 109

Median fin rays [Modified definition of character of character 103 of Forey 1998]

0. slender
1. expanded

Character 110

Lateral line openings in scales [character 105 of Forey 1998]

0. single
1. multiple

Character 111

Ventral keel scales [character 109 of Gess & Coates 2015]

0. absent
1. present

Character 112

Scale ornament [character 104 of Forey 1998]

0. not differentiated
1. differentiated

SI 2.2 Characters not included in the present analysis

Former character 1

Intracranial joint margin [Forey 1998; character 1]

0. straight
1. strongly interdigitate

Arratia & Schultze (2015) added a third state '2. undulated' in order to include *Atacamaia solitaria*. Toriño et al. (2021) included this this character state in their phylogenetic analysis.

Intracranial joint margin [Arratia & Schultze 2015, character 1; Toriño et al. 2021, character 1]

0. straight
1. strongly interdigitate
2. undulated

Observing the matrices, this state is present only in *A. solitaria* (Arratia & Schultze, 2015; Toriño et al., 2021). Arratia & Schultze (2015) did not precisely defined this character and did not review it for all actinistians. Therefore, the state added by Arratia & Schultze (2015) is deleted and the definition of Forey (1998) is restored.

Former character 4

Premaxillae [Forey 1998; character 4]

0. paired
1. fragmented

This character was created by Cloutier (1991) and used later by Forey (1998) and successive works (e.g. Toriño et al., 2021). A 'Fragmented' premaxillae condition is regarded as the derived condition (Cloutier 1991; Forey, 1998). Only *Coelacanthus granulatus* (Schaumberg, 1978), *Latimeria* (Forey, 1998) and *Parnaibaia* (Yabumoto 2008) show this condition. Cloutier (1991a) pointed out that there is no evidence that the bones observed as 'fragmented' premaxillae are homologous to a single premaxilla despite their topographic position and the presence of teeth on all of them. In *Latimeria*, a 'fragmented' condition of the premaxillae is present at the embryo stage (Forey, 1998) and persists into the adult (Smith, 1939 cited by Forey, 1998). However, Forey (1998) cites that Schultze & Cloutier (1991) may had suggest that the premaxillae are paired into some specimens. Unfortunately, nothing is written about this hypothesis in the article aforementioned. Recently, Mahé et al. (2021) shown that the lifespan of *Latimeria* is probably around 100 years and that this animal reaches its sexual maturity between 49 and 69 giving birth after a gestation time of 5 years, which is the longest known. Considering this peculiar life characteristic, it could be hypothesised that the 'fragmented' premaxillae condition is linked to the skeletal development. In this case the "fragmented" premaxillae fuse together to form a paired premaxilla when the somatic

maturity is reached. This would mean that the specimen observed by Smith (1939 cited by Forey, 1998) is a ‘young’ specimen and that the other specimens from Schultze & Cloutier (1991) are older one. This hypothesis is also congruent with *Parnaibaia* (Yabumoto, 2008) which may surely be a juvenile specimen as shown by its very elongated caudal lobe. Unfortunately, the case of the *C. granulatus* described by Schaumberg (1978) cannot be assessed.

If the hypothesis of a progressive fusion of the ‘fragmented’ premaxillae into a paired premaxilla is verified, this character is therefore not phylogenetically significant and should not be used into phylogenetic analyses.

As new observations, especially on *Latimeria* of different ontogenic stages, are required to decide, this character is maintained.

Former character 21

Otic canal [Forey 1998; character 21]

0. joining supratemporal canal within lateral extrascapular
1. in supratemporal

According to Forey (1998), actinistians may have the triple junction of otic canal, lateral line and supratemporal commissure occurring either in the lateral most extrascapular either into the supratemporal.

The embayed condition implies that the extrascapular series is comprised between the supratemporals while in the straight condition the extrascapular series lies posterior to the supratemporal.

Posterior margin of the skull roof [character 17; character 18 of Forey 1998]

0. straight
1. embayed

Forey (1998) mentioned that this character may be associated with the location of the triple junction of otic canal, lateral line and supratemporal commissure (character 21). Regarding the available data, it is obvious that those two characters are always linked. Indeed, an embayed state automatically implies a triple junction occurring in the supratemporal and, reversely, a triple junction located in the extrascapular implies a straight condition. According to the scoring of Forey (1998), the only exception is *Polyosteorhynchus* with a straight posterior margin and with a triple junction located in the supratemporal. Lund & Lund (1985) reported that no pores are present on the supratemporal of *Polyosteorhynchus*. As Forey (1998) did not describe or discuss this weird condition, this single exception is considered as doubtful and rather an error of scoring.

Forey (1991 and 1998) also mentioned that both characters 18 and 21 may be associated with the hypothesised phylogenetic fusion between the lateral most extrascapular and the supratemporal. As suggested by Stensiö (1921), and agreed by Forey (1998), this fused bone may be named a

supratemporo-extrascapular. However, Forey (1998) did not retain this nomenclature because of the uncertainty regarding the process of this fusion.
Therefore, both characters 18 and 21 are strongly linked together, and one must be deleted to avoid redundancy. The positioning of a triple junction of a canal is a strong character that can be recognised without ambiguity. Unfortunately, the location of this triple junction is not always obvious and observable directly. Furthermore, it is most likely that in some instances the positioning of the triple junction was deduced from the condition of the embayment of the skull, but not from a direct observation. Therefore, the character concerning the triple junction (character 21) is deleted.
Regarding ambiguous patterns, as for instance *Coccoderma* having a margin between the postparietals-supratemporal and the lateral extrascapular series curved (Forey, 1998, fig. 3.10B) it is proposed to reformulated the character of Forey (1998). This reformulation of this character doesn’t affect any scoring.

Former character 24

Anterior pit line [Forey 1998; character 24]

0. absent
1. present

The anterior pit line is a characteristic found in actinopterygians and porolepiforms. According to Forey (1998), all actinistians, with the possible exception of *Diplocercides*, lack an anterior pit line. Stensiö (1937, figs 1-3) and Forey (1998, fig. 3.4) have labelled an anterior pit line on the postparietals of *Diplocercides*. Forey (1998) claims that this identification may be incorrect arguing that *Latimeria* has also an anterior line not innervated by the nerve.
According Hensel & Balon (2001), the pit lines of *Latimeria* are pit-lines canals covered by a thin epidermis and not lines of superficial neuromasts. As a tentative explanation, the structure labelled as an anterior pit line by Forey (1998, fig. 3.4) could potentially be the middle pit line.
The character labelled by Stensiö (1937) may be rather regarded as the opening for the median branch of the otic canal, although Forey (1998) claimed that there is there is no evidence of such character. Therefore, the use of this character, doubtfully present in only one taxon and present in the outgroups, is not informative and bring uncertainties.
Based on this short discussion, the character 24 of Forey (1998) is deleted.

Former character 31

Preoperculum [Forey 1998; character 31]

0. absent
1. present

According Forey (1998), some actinistians may lack of a preopercle. Regarding his datamatrix, an absence of the preopercle is scored only for *Euporosteus*, *Wimania* and *Laugia*. As the cheek of *Euporosteus* is unknown, the score of Forey (1998) is clearly an error. Stensiö (1921) reported the

presence of a preopercle in the cheek of *Wimania*. The presence of this bone is recalled by Forey (1998) himself, meaning that his scoring is a mistake. *Laugia* is known from several specimens and the cheek is composed of a lachrymojugal, postorbital and squamosal that do not contact each other (Forey, 1998). The cheek is said to be similar to the cheek of *Coccoderma* (Forey, 1998) except for the presence of a preopercle reduced to a narrow tube. It is reasonable to think that the absence of the preopercle of *Laugia* is more due to a taphonomic process rather than a true biological absence. Indeed, a small bone not sutured to another bone can easily be lost or not preserved. The preopercle of *Gavinia* is missing (Long, 1999). Zhu et al. (2012) scored this state as unknown in *Gavinia*, contrary to Gess & Coates (2015) and Toriño et al. (2021) who scored it as absent. Due to the crushed aspect of the specimen described by Long (1999), the absence of the preopercle of *Gavinia* is probably rather due to a taphonomic process rather than a true biological absence.

Therefore, this character dealing with the absence/presence of the preopercle is deleted as it is not clearly absent in actinistians, with the possible exception of *Laugia* from which the case seems to doubtful.

Former character 33

Quadratojugal [Forey 1998; character 33]

0. absent
1. present

Since Forey (1998) created this character, a separate quadratojugal has never been formerly identified in any actinistians. A separate quadratojugal has been reported in *Diplocercides heiligenstockensis* (Cloutier, 1991), in *Hadronector* (Lund & Lund, 1985; Cloutier, 1991), *Allenypterus*, *Caridosuctor*, *Polyosteorhynchus* and *Lochmocercus* (Lund & Lund, 1985). In those taxa, the presence of this bone is discussed by Forey (1998) who concluded that only few and perhaps no actinistian has a separate quadratojugal. In *Allenypterus*, *Caridosuctor*, *Polyosteorhynchus* and *Lochmocercus*, Forey (1998) was unable to confirm the presence of a separate quadratojugal as reported by Lund & Lund (1985) founding difficult to accept the restoration provided by the previous authors. According Forey (1998), there is no separate quadratojugal in *Diplocercides* species. In the case of *Hadronector*, the bone in question is named quadratojugal by Coutier (1991) after the pit line, while Forey (1998) named it after the contained sensory canal. Among other sarcopterygians, it is worth noting that a separate quadratojugal is absent from *Onychodus jandemarrai*.

Based on this short discussion, the character 33 of Forey (1998) is deleted as it brings no relevant information being then phylogenetically uninformative.

Former character 62

Ornaments [Forey 1998; character 62]

0. ridged
1. granular

With this character, Forey (1998) defined several types of ornamentation for the lower jaw. It is worth noting that Forey (1998) didn’t explain on which bone the ornamentation is scored and referred only to the lower jaw. As explained in character 28 (section ‘3.2. Cheek bones and sensory canals’ in the main article), a new definition dealing with the ornamentation on dermal bones is formulated and this character of Forey (1998) is deleted from this analysis.

Former character 63

Dentary [Forey 1998; character 63]

0. with ornament
1. without ornament

A detailed information concerning the deletion of this character can be found in the definition of the character 28 at the section ‘3.2. Cheek bones and sensory canals’ in the main article.

Former character 64

Splenial [Forey 1998; character 64]

0. with ornament
1. without ornament

A detailed information concerning the deletion of this character can be found in the definition of the character 28 at the section ‘3.2. Cheek bones and sensory canals’ in the main article.

Former character 70

Optic foramen [Forey 1998; character 70]

0. enclosed by basisphenoid extending forward
1. lying within separate interorbital ossification or cartilage

The formulation of the character of Forey (1998) does not allow to properly score in most of ingroup taxa. We thus do not retain this character.

Former character 73

Antotic process [Forey 1998; character 73]

0. not covered by parietal descending process
1.covered

Regarding the definition of this character, it is is then linked to the condition of the parietal descending process (character 11). Regarding the distribution of those two characters, only two known cases appear. When the parietal descending process is absent, the antotic process is not covered (*Diplocercides* and *Euporosteus*), which is, by definition, obvious. Indeed, if the parietal descending process is absent it would not never cover the antotic process. When the parietal descending process is present, the antotic process is covered (e.g. *Latimeria*). In some taxa possessing a parietal descending process, it is not known if the antotic process is covered or not (e.g. *Mawsonia*). Although, no case of the presence of parietal descending process that not cover the antotic process, which would be the other valid combination, have been reported. Based on this observation, the character 73 of Forey (1998) is deleted.

Former character 77

Vestibular fontanelle [Forey 1998; character 77]

0. absent
1. present

Forey (1998) identified a structure as the vestibular fontanelle in *Diplocercides*, *Laugia*, *Sassenia* and *Spermatodus*. Friedman (2007) claims that the structure identified as a vestibular fontanelle by Forey (1998) is not homologous to the same structure observed in actinopterygians and other sarcopterygians. Based on this assumption, the character 77 of Forey (1998) is deleted.

Former character 106

Scales [Forey 1998; character 106]

0. ornament of ridges or tubercles
1. rugose

As mentioned by Toriño et al. (2021), this character is inconsistent for several taxa and needs to be reviewed. Therefore, this character is abandoned since it is reviewed.

References

Arratia G, Schultze H-P (2015) A new fossil actinistian from the Early Jurassic of Chile and its bearing on the phylogeny of Actinistia. Journal of Vertebrate Paleontology 35: e983524.

Cloutier R (1991) Interrelationships of Palaeozoic actinistians: patterns and trends. Early Vertebrates and Related Problems of Evolutionary Biology: Science Press, Beijing: 379-428.

Dutel H, Maisey JG, Schwimmer DR, Janvier P, Herbin M, et al. (2012) The giant Cretaceous Coelacanth (Actinistia, Sarcopterygii) Megalocoelacanthus dobiei Schwimmer, Stewart & Williams, 1994, and its bearing on Latimerioidei interrelationships. PLoS ONE 7: e49911.

Ferrante C, Cavin L (2023) Early Mesozoic burst of morphological disparity in the slow-evolving coelacanth fish lineage. Scientific Reports 13: 11356.

Forey PL (1998) History of the Coelacanth Fishes. London: Chapman and Hall.

Friedman M (2007) *Styloichthys* as the oldest coelacanth: implications for early osteichthyan interrelationships. Journal of Systematic Palaeontology 5: 289.

Gess RW, Coates MI (2015) Fossil juvenile coelacanths from the Devonian of South Africa shed light on the order of character acquisition in actinistians. Zoological Journal of the Linnean Society 175: 360-383.

Hensel K, Balon EK (2001) The sensory canal systems of the living coelacanth, *Latimeria chalumnae*: A new instalment. Environmental Biology of Fishes, 61, 117-124.

Long JA (1999) A new genus of fossil coelacanth (Osteichthyes: Coelacanthiformes) from the Middle Devonian of southeastern Australia. Records of the Western Australian Museum, Supplement 57: 37-53.

Lund WL, Lund R, Klein G (1985) Coelacanth feeding mechanisms and ecology of the Bear Gulch coelacanths. Compte Rendus du Neuvième Congrès International sur la Stratigraphie et la Géologie du Carbonifère 5: 492-500.

Mahé K, Ernande B, Herbin M (2021) New scale analyses reveal centenarian African coelacanths. Current Biology.

Schaumberg G (1978) Neubeschreibung yon Coelacanthus granulatus Agassiz (Actinistia, Pisces) aus dem Kupferschiefer yon Richelsdorf (Perm, W.-Deutschland). Palaeontologische Zeitschrift 52: 169-197.

Schultze HP, Cloutier R (1991) Computed tomography and magnetic resonance imaging studies of *Latimeria chalumnae*. The biology of *Latimeria chalumnae* and evolution of coelacanths, 159-181.

Stensiö EA (1921) Triassic fishes from Spitzbergen: Almqvist & Wiksells.

Stensiö E (1937) On the Devonian Coelacanthids of Germany with Special Reference to the Dermal Skeleton, &c.

Toriño P, Soto M, Perea D (2021) A comprehensive phylogenetic analysis of coelacanth fishes (Sarcopterygii, Actinistia) with comments on the composition of the Mawsoniidae and Latimeriidae: Evaluating old and new methodological challenges and constraints. Historical Biology: 1-21.

Yabumoto Y (2008) A New Mesozoic coelacanth from Brazil (Sarcopterygii, Actinistia). Paleontological Research 12: 329-343.

Zhu M, Yu X, Lu J, Qiao T, Zhao W, et al. (2012) Earliest known coelacanth skull extends the range of anatomically modern coelacanths to the Early Devonian. Nature Communications 3: 772.

S2.3 Datamatrix

Taxon / Character	1	2	3	4	5	6	7	8	9	10	11	12	13	14	15	16	17	18	19	20	21	22	23	24	25	26	27	28	29	30	31	32	33	34	35	36	37	38
Onychodus (outgroup)	0	0	0	0	0	-	0	0	-	0	0	0	1	1	0	0	0	0	0	0	0	0	2	0	0	0	0	0	0	1	0	0	0	0	0	0	0	0
Allenypterus	0	1	?	0	?	?	1	1	0	0	?	0	1	0	?	?	0	0	0	0	0	1	0	0	0	0	0	0	1	?	0	0	1	0	1	0	0	0
Atacamaia	0	?	?	?	?	?	?	0	-	1	?	?	1	0	?	?	1	1	0	?	?	?	2	1	?	0	1	0	?	?	0	0	1	1	?	?	?	?
Axelia	0	0	?	?	?	?	1	0	?	0	1	0	1	0	?	1	1	1	0	2	1	1	0	?	0	?	?	?	?	?	?	?	?	?	?	?	?	?
Axelrodichthys	0	1	?	1	1	0	1	1	1	0	1	0	0	0	1	0	1	0	1	1	0	1	2	0	0	1	?	2	1	0	0	1	0	1	1	0	0	0
Caridosuctor	0	0	0	{01}	0	0	1	1	1	0	?	1	1	0	?	?	0	0	0	1	0	1	0	1	0	0	1	0	0	1	0	0	0	0	1	0	?	0
Chinlea	0	1	?	0	1	?	1	1	{01}	0	1	{01}	0	0	?	?	1	0	1	2	{01}	?	?	1	0	?	?	2	1	?	0	0	0	1	1	0	?	0
Coccoderma	0	0	?	?	1	1	?	1	1	0	1	0	1	0	0	1	0	0	0	1	0	1	2	1	0	0	1	3	1	0	0	0	0	0	1	1	?	1
Coelacanthus	0	0	0	?	1	?	?	1	1	0	1	0	0	0	0	1	1	0	0	1	0	?	?	1	0	1	?	3	1	?	0	0	1	0	1	1	0	?
Diplocercides	0	0	0	?	?	?	0	1	1	0	0	1	1	0	0	0	0	0	0	0	0	1	2	0	0	0	0	0	0	1	0	0	0	0	1	0	1	0
Diplurus	0	1	0	0	1	1	1	1	1	0	1	0	0	0	1	0	1	1	0	2	?	1	0	1	0	1	?	3	1	1	0	0	1	0	1	1	0	1
Dobrogeria	0	?	?	?	?	?	?	?	?	0	1	?	?	0	1	1	1	1	0	?	?	1	?	1	?	1	?	0	1	?	?	?	?	?	?	?	?	0
Euporosteus	0	0	?	?	?	?	0	1	1	?	0	?	1	?	?	?	0	?	?	?	0	?	0	?	?	?	?	?	?	?	?	?	?	?	?	?	?	?
Foreyia	1	0	0	?	1	1	1	1	0	0	?	0	1	0	?	?	?	?	?	?	?	1	4	?	?	?	?	1	1	0	1	0	1	0	1	?	0	?
Garnbergia	0	1	?	?	?	?	?	1	?	?	?	0	0	0	?	?	1	?	?	?	?	?	?	?	?	?	?	3	1	?	0	?	0	0	1	0	0	?
Gavinia	?	?	0	0	0	0	?	?	?	?	?	?	?	1	?	?	?	?	?	?	?	0	?	?	?	?	?	0	0	?	0	0	0	?	?	0	1	?
Guizhoucoelacanthus	0	1	0	?	?	?	1	1	0	0	?	0	1	0	?	?	1	0	0	1	0	1	2	1	0	0	0	3	1	?	0	0	0	0	1	0	0	?
Hadronector	0	1	0	0	0	0	0	1	1	0	?	?	1	0	?	?	0	0	0	0	0	1	1	0	0	0	1	0	0	1	0	0	0	0	1	0	0	0
Heptanema	0	1	0	?	?	?	?	1	0	0	?	?	?	?	?	?	?	?	?	?	?	?	?	?	?	?	?	3	1	?	?	?	?	?	1	?	0	?
Holophagus	0	1	0	?	1	?	1	1	1	0	1	1	0	0	1	1	1	1	0	?	?	1	2	1	?	1	?	3	1	0	0	0	0	0	1	0	0	0
Holopterygius	?	?	0	?	?	?	?	?	?	?	?	?	?	?	?	?	?	?	?	?	?	?	0	?	?	?	?	?	?	?	?	?	?	?	?	?	?	?
Indocoelacanthus	0	?	?	?	?	?	?	1	?	0	?	?	?	0	?	?	?	?	?	?	?	1	2	?	?	?	?	2	1	1	0	?	0	?	1	0	0	0
Latimeria	0	1	0	1	1	1	1	1	1	1	1	1	0	0	1	1	1	1	0	2	0	1	0	1	1	1	1	3	1	1	1	0	0	0	1	0	0	0
Laugia	0	0	1	0	?	?	?	0	?	0	1	0	1	0	0	1	0	0	0	1	0	1	2	1	0	1	?	0	1	0	0	0	0	0	1	0	0	?
Libys	0	1	1	?	{01}	?	1	1	?	0	1	?	0	0	1	1	1	1	0	?	?	1	3	1	?	1	?	3	1	0	0	0	0	0	1	0	0	0
Lochmocercus	0	?	?	?	?	?	?	?	?	0	?	?	1	?	?	?	?	?	?	?	?	1	0	0	0	?	?	?	0	1	0	0	0	?	?	0	?	0
Lualabaea	0	1	?	?	?	?	?	?	?	0	?	?	?	0	?	?	?	0	1	?	0	?	?	?	?	?	?	2	?	?	?	?	?	?	?	?	?	?
Luopingcoelacanthus	0	?	?	1	1	?	?	1	1	0	?	0	?	0	1	?	1	1	0	?	?	?	?	?	?	1	?	?	1	?	0	0	0	?	?	0	0	0
Macropoma	0	1	1	1	?	1	1	1	1	0	1	1	0	0	1	1	1	1	0	2	0	1	{02}	1	1	1	?	1	1	0	1	0	0	0	1	0	0	0
Mawsonia	0	1	?	?	?	?	1	1	1	0	1	0	0	0	1	0	1	0	1	1	1	1	2	1	0	1	?	2	1	0	0	1	0	1	1	0	0	0
Megalocoelacanthus	0	1	1	?	0	1	1	1	1	0	1	?	?	0	1	1	1	1	0	?	?	1	3	1	?	?	?	3	1	?	?	?	?	?	?	?	?	?
Miguashaia	0	0	0	0	0	0	?	0	?	0	0	0	?	1	?	?	0	0	0	0	0	0	2	0	0	0	0	0	0	?	0	0	0	0	0	0	1	0
Ngamugawi	0	0	0	0	0	0	0	1	1	0	0	?	?	0	0	0	0	0	0	?	0	1	0	1	?	0	0	0	0	0	0	0	0	0	1	0	1	0
Parnaibaia	0	1	0	0	?	?	1	1	0	0	?	0	0	0	?	?	?	0	?	2	1	?	2	1	?	0	0	2	1	0	0	0	0	?	1	0	0	?
Piveteauia	0	0	?	?	?	?	?	?	?	0	1	?	?	0	1	1	1	?	0	?	?	?	?	1	?	?	?	0	?	1	?	?	?	?	?	0	0	?
Polyosteorhynchus	0	1	0	1	0	0	?	1	1	0	?	0	1	?	?	1	0	0	0	0	0	1	1	0	0	?	?	0	0	1	0	0	0	0	1	0	0	0
Rebellatrix	?	?	?	?	?	?	?	?	?	?	?	?	?	?	?	?	?	?	?	?	?	?	?	?	?	?	?	?	?	?	?	?	?	?	?	?	?	?
Reidus	?	?	?	?	?	?	?	?	?	?	?	?	?	?	?	?	?	?	?	?	?	?	?	?	?	?	?	?	1	?	?	?	?	?	?	0	0	0
Rhabdoderma	0	0	0	1	0	0	1	1	0	0	1	0	1	0	0	1	0	0	0	1	0	1	0	1	0	0	1	0	0	1	0	0	0	0	1	0	0	0
Rieppelia	1	0	0	?	0	1	1	1	0	0	1	0	1	0	1	1	0	0	?	0	0	1	0	0	0	1	?	1	0	0	0	0	?	0	1	?	?	0
Sassenia	0	0	0	?	?	?	?	1	?	0	1	0	1	0	0	1	0	0	0	?	?	1	2	?	0	0	1	1	1	1	0	0	0	0	1	0	1	0
Serenichthys	0	0	0	?	?	0	?	1	0	0	?	?	1	0	?	?	0	0	0	1	0	?	?	?	?	?	?	0	0	1	0	0	0	0	1	0	0	0
Spermatodus	0	0	0	0	0	0	1	1	1	0	1	0	1	0	0	1	0	1	0	?	0	1	2	1	0	1	?	0	0	?	0	0	0	?	1	0	1	0
Swenzia	0	1	1	?	?	?	?	1	?	1	?	?	?	0	1	1	1	?	?	?	?	?	?	?	1	0	1	3	1	1	1	0	0	0	1	0	0	0
Ticinepomis	0	1	?	1	?	?	?	1	0	0	1	?	1	0	1	1	0	1	?	?	?	?	2	?	?	?	?	0	1	?	?	0	1	?	?	1	?	0
Trachymetopon	0	1	?	?	?	?	?	?	?	0	1	?	?	0	1	0	1	0	1	1	0	?	?	?	0	1	?	2	1	?	?	?	0	?	?	?	?	?
Undina	0	1	0	?	0	?	?	1	1	1	1	1	0	0	1	1	1	1	0	2	?	1	2	?	?	?	?	3	1	0	0	0	0	0	?	0	0	0
Whiteia	0	1	0	{01}	0	0	1	1	0	0	1	0	1	0	0	1	1	1	0	{12}	0	1	2	1	0	0	1	3	1	1	0	0	0	0	1	0	0	0
Wimania	?	?	?	?	?	?	?	0	?	?	1	?	?	0	0	1	?	?	?	?	?	?	?	?	?	?	?	?	1	?	?	0	0	?	1	0	?	0
Yunnancoelacanthus	0	0	?	?	?	?	0	1	0	0	?	0	1	0	1	1	1	0	1	1	0	?	?	?	?	1	0	0	1	?	0	0	0	?	1	0	0	0

Taxon / Character	39	40	41	42	43	44	45	46	47	48	49	50	51	52	53	54	55	56	57	58	59	60	61	62	63	64	65	66	67	68	69	70	71	72	73	74	75	76	77
Onychodus (outgroup)	0	0	1	-	-	-	-	-	-	0	1	0	0	0	0	0	0	1	-	0	0	0	-	0	0	1	0	0	0	-	-	-	0	-	1	0	?	?	0
Allenypterus	0	1	1	0	0	0	0	0	0	0	?	?	?	1	1	3	0	1	0	0	0	1	0	?	?	?	?	0	0	0	0	1	?	?	?	?	?	?	?
Atacamaia	?	?	0	1	0	0	?	?	?	?	0	?	?	?	?	0	0	1	?	?	?	?	?	?	?	?	?	?	?	?	?	?	?	?	?	?	?	?	?
Axelia	?	?	?	?	?	?	?	0	?	?	?	?	?	?	?	0	?	1	?	0	?	?	0	?	?	0	1	?	1	?	?	?	?	?	?	?	?	?	0
Axelrodichthys	0	1	0	1	0	0	?	1	1	?	0	0	0	0	1	2	1	0	1	1	1	1	1	0	0	0	1	0	1	1	1	?	1	0	0	1	1	1	0
Caridosuctor	0	0	1	0	0	0	?	0	0	?	?	0	0	0	0	0	0	?	0	0	0	1	0	0	1	1	?	0	1	0	0	0	?	?	?	?	?	?	?
Chinlea	0	1	0	1	0	0	0	1	1	?	0	?	?	0	?	2	1	0	?	1	0	?	0	?	1	1	?	?	1	?	1	?	?	?	?	?	?	?	?
Coccoderma	0	0	0	0	0	0	0	0	?	1	0	0	1	0	1	3	0	1	?	0	0	1	0	0	1	0	?	0	1	0	0	0	?	?	?	?	?	?	?
Coelacanthus	?	?	?	?	0	?	?	?	?	?	0	?	0	0	?	0	?	1	0	0	0	1	0	0	1	0	0	0	1	0	1	?	?	?	?	?	?	?	?
Diplocercides	0	1	1	0	{01}	0	?	0	0	0	0	0	0	0	0	0	0	1	0	0	0	0	0	1	0	0	0	0	0	0	0	1	0	1	1	1	0	0	0
Diplurus	0	1	0	1	0	0	0	{12}	1	0	0	?	?	1	1	3	0	0	1	?	1	?	0	?	0	0	0	0	1	1	?	?	1	0	0	?	1	1	0
Dobrogeria	0	?	1	1	0	?	?	?	?	?	?	?	?	?	?	0	?	?	1	?	?	?	?	?	?	?	?	?	1	0	0	0	1	1	0	0	1	?	?
Euporosteus	?	?	?	?	?	?	?	?	?	?	?	?	?	?	?	?	?	?	?	?	?	?	?	?	?	?	?	?	?	?	?	?	0	1	?	?	?	?	0
Foreyia	0	1	1	1	1	0	0	0	1	0	0	?	0	0	1	1	0	0	?	1	0	1	0	0	0	1	0	?	1	0	?	?	?	0	?	?	?	?	?
Garnbergia	?	?	0	?	0	0	?	?	?	?	?	?	?	?	?	3	0	0	?	?	?	?	?	?	?	?	?	?	?	?	?	?	?	?	?	?	?	?	?
Gavinia	?	0	?	?	?	?	?	?	?	0	0	0	0	0	0	0	0	?	0	0	0	0	?	?	?	?	?	?	0	?	?	?	?	?	?	?	?	?	?
Guizhoucoelacanthus	0	1	?	1	0	0	?	0	1	?	0	?	0	0	?	3	0	0	?	0	0	?	0	?	?	0	?	?	1	?	?	0	?	?	?	?	?	?	?
Hadronector	0	1	1	0	0	0	?	0	0	?	1	?	0	0	?	0	0	1	0	0	0	?	0	?	?	?	?	0	0	0	0	0	?	?	?	?	?	?	?
Heptanema	0	?	?	1	0	?	?	?	?	?	?	?	?	?	?	3	0	0	?	1	?	?	?	?	?	?	?	?	?	?	?	?	?	?	?	?	?	?	?
Holophagus	0	1	1	1	0	0	?	?	{12}	?	1	?	?	0	1	1	0	0	1	1	0	?	0	?	1	1	?	1	1	0	0	0	?	?	?	?	1	1	?
Holopterygius	?	?	?	?	?	?	?	?	?	?	?	?	?	?	?	?	?	?	0	0	?	0	?	?	?	0	0	?	?	?	?	?	?	?	?	?	?	?	?
Indocoelacanthus	0	1	?	?	0	?	?	?	?	?	?	?	?	0	?	2	?	?	?	1	1	1	0	?	?	?	?	?	?	?	?	?	?	?	?	?	?	?	?
Latimeria	1	1	1	1	0	1	1	2	2	1	1	1	1	1	1	1	0	0	1	1	0	1	0	0	1	1	0	1	1	0	0	0	1	0	0	0	1	1	1
Laugia	?	?	0	0	0	0	0	0	1	1	0	0	1	0	1	0	0	1	0	0	0	1	0	0	0	0	0	0	1	0	0	0	1	1	0	1	1	0	0
Libys	1	1	0	1	0	1	?	?	?	?	1	?	1	2	?	3	0	1	?	1	0	1	0	0	1	0	1	1	1	?	0	0	1	?	?	?	?	?	1
Lochmocercus	0	?	1	?	0	0	?	0	0	0	1	0	0	0	0	?	0	1	?	0	0	0	0	?	0	0	?	0	0	?	?	?	?	?	?	?	?	?	?
Lualabaea	?	?	?	1	0	?	?	1	1	?	?	?	?	?	?	2	0	?	?	1	1	1	1	?	?	?	?	?	?	?	?	?	?	?	?	?	?	?	?
Luopingcoelacanthus	0	?	0	?	1	?	?	?	?	?	?	0	0	0	1	?	0	1	1	1	0	1	0	?	?	0	?	?	1	?	?	?	1	0	?	?	?	?	0
Macropoma	1	1	?	1	0	1	1	1	1	1	1	0	1	0	1	1	0	0	1	1	0	1	0	0	?	0	0	1	1	0	0	0	1	0	0	0	1	1	1
Mawsonia	0	1	0	1	0	1	0	1	1	?	0	0	0	0	1	2	1	0	1	1	1	1	{01}	?	?	0	{01}	?	1	1	1	?	?	1	0	?	1	1	0
Megalocoelacanthus	?	?	?	?	?	?	?	?	?	?	?	?	?	2	?	3	?	?	1	1	0	1	0	?	0	0	1	1	1	0	1	?	1	0	0	1	1	?	1
Miguashaia	0	0	1	?	0	?	?	?	?	0	0	0	0	0	?	0	0	1	?	0	0	0	0	?	?	0	0	?	?	0	{01}	0	?	?	?	?	?	?	?
Ngamugawi	0	1	1	0	1	?	?	0	0	0	0	0	0	0	0	0	0	?	0	0	0	1	0	1	0	0	1	0	0	0	0	1	0	0	1	?	0	0	0
Parnaibaia	?	?	?	1	0	0	0	1	1	?	?	?	?	?	?	2	1	0	1	1	1	?	?	?	0	0	0	?	1	1	?	?	?	?	?	?	?	?	?
Piveteauia	?	0	?	0	?	?	0	?	?	?	0	1	0	?	?	0	0	1	1	0	0	1	0	?	?	?	0	?	1	?	?	?	?	?	?	?	?	?	0
Polyosteorhynchus	0	1	1	0	0	0	?	0	0	?	1	?	0	0	0	0	0	1	0	0	0	1	0	?	0	0	?	0	1	0	0	0	?	?	?	?	?	?	?
Rebellatrix	?	?	?	?	?	?	?	?	?	?	?	?	?	?	?	?	?	?	1	?	?	?	?	?	?	?	?	?	?	?	0	?	?	?	?	?	?	?	?
Reidus	0	?	?	?	?	?	?	?	?	?	?	?	?	?	1	3	?	?	1	1	1	1	0	0	0	0	0	?	1	1	0	0	?	?	?	?	?	?	?
Rhabdoderma	0	0	1	0	0	0	0	0	0	0	1	1	0	0	0	0	0	1	0	0	0	1	0	0	1	1	0	0	1	0	0	0	1	1	0	1	0	?	0
Rieppelia	0	1	0	?	1	0	1	2	2	?	?	?	?	1	1	1	0	0	?	?	?	1	0	0	0	1	0	?	?	?	?	?	1	?	0	?	1	?	?
Sassenia	0	0	1	0	0	0	0	0	0	0	1	0	0	0	0	1	0	1	?	0	0	1	0	?	1	?	?	0	1	0	1	?	0	1	0	1	0	0	?
Serenichthys	0	1	1	0	1	0	0	?	?	0	1	?	0	0	?	0	0	1	?	0	?	0	0	?	?	?	?	?	?	?	?	?	?	?	?	?	?	?	?
Spermatodus	0	0	1	0	0	0	?	0	0	?	?	?	0	0	0	0	0	1	0	?	?	1	0	0	1	1	1	0	?	?	0	0	1	1	0	?	?	0	0
Swenzia	1	1	?	1	0	?	1	1	1	1	1	0	1	0	1	1	0	0	?	1	0	1	?	?	?	?	0	?	1	0	0	0	?	?	?	?	?	?	?
Ticinepomis	0	?	?	1	1	?	1	?	?	?	?	?	?	1	?	0	0	0	1	{01}	0	1	0	?	?	1	0	1	1	0	?	?	1	0	0	0	1	?	0
Trachymetopon	?	?	?	?	0	?	?	?	?	?	?	?	?	?	?	2	?	0	1	1	0	1	1	?	?	0	?	0	1	0	?	0	1	1	0	?	1	?	?
Undina	0	?	1	1	?	0	?	?	?	?	1	0	1	0	1	0	0	0	1	1	0	?	0	?	1	1	0	?	1	0	0	0	1	0	0	0	1	1	1
Whiteia	0	1	1	1	0	0	0	0	0	1	1	1	0	0	0	0	0	1	1	1	0	1	0	0	1	0	0	?	1	0	0	0	1	0	0	1	1	?	0
Wimania	0	1	?	1	0	0	?	?	?	?	?	?	0	?	?	?	1	1	?	0	?	?	?	?	1	?	0	?	?	?	?	?	?	?	?	1	?	?	?
Yunnancoelacanthus	0	?	0	?	?	0	?	?	?	?	?	?	?	?	1	0	0	?	?	0	?	0	0	?	?	0	0	0	?	?	?	?	?	?	?	?	?	?	?

**Taxon / Character**	**78**	**79**	**80**	**81**	**82**	**83**	**84**	**85**	**86**	**87**	**88**	**89**	**90**	**91**	**92**	**93**	**94**	**95**	**96**	**97**	**98**	**99**	**100**	**101**	**102**	**103**	**104**	**105**	**106**	**107**	**108**	**109**	**110**	**111**	**112**
Onychodus (outgroup)	1	0	1	-	0	-	?	1	0	0	?	?	?	0	0	?	?	?	?	?	1	0	0	?	?	?	1	?	0	0	?	?	1	?	0
Allenypterus	?	?	?	?	?	?	?	?	?	?	0	?	?	1	0	0	1	0	0	1	0	0	0	0	0	0	1	1	0	1	0	0	0	1	0
Atacamaia	?	?	?	?	?	?	?	?	?	?	0	?	?	?	?	?	?	?	?	?	?	?	?	?	?	?	?	?	?	?	?	?	?	0	?
Axelia	?	?	?	?	?	?	?	?	?	?	0	?	?	?	?	?	?	?	?	?	?	?	1	?	?	?	?	?	?	?	0	?	?	?	0
Axelrodichthys	0	0	0	1	1	0	?	1	1	1	0	1	0	1	0	?	1	1	1	1	0	{01}	1	1	0	0	1	0	1	1	0	0	?	0	1
Caridosuctor	?	?	?	?	?	?	?	?	?	?	?	?	?	1	0	0	1	?	0	1	1	0	0	1	0	0	1	1	1	1	0	0	?	0	0
Chinlea	?	?	?	?	?	?	?	?	?	1	?	?	?	1	?	?	1	?	1	?	?	1	0	1	0	0	1	?	1	1	0	0	?	0	1
Coccoderma	?	?	?	?	?	?	?	?	?	?	0	?	?	1	1	0	1	1	0	1	0	{01}	0	1	1	1	1	0	1	1	1	0	1	0	0
Coelacanthus	?	?	?	?	?	?	?	?	?	?	0	?	?	1	?	0	1	0	0	1	0	0	0	1	0	0	1	0	1	1	0	0	?	0	0
Diplocercides	1	0	1	?	0	1	0	0	0	0	0	?	?	?	?	?	0	0	0	?	?	0	0	?	0	?	1	0	0	1	0	0	0	?	0
Diplurus	0	0	?	?	?	0	?	1	1	1	0	1	0	1	0	{01}	1	1	1	0	0	{01}	1	1	0	0	1	0	1	1	0	0	?	0	1
Dobrogeria	?	?	?	?	?	0	?	?	1	1	0	?	?	1	?	?	?	?	?	?	?	?	?	?	?	?	?	?	?	?	?	?	?	?	?
Euporosteus	1	0	1	0	?	1	?	0	?	0	?	?	?	?	?	?	?	?	?	?	?	?	?	?	?	?	?	?	?	?	?	?	?	?	?
Foreyia	?	1	?	?	?	?	?	1	?	?	1	?	?	1	0	1	1	?	0	0	?	0	1	?	0	?	1	0	1	1	0	0	?	?	1
Garnbergia	?	?	?	?	?	?	?	?	?	?	?	?	?	?	?	?	?	?	?	?	0	?	0	1	?	?	?	?	?	?	?	0	?	0	0
Gavinia	?	?	?	?	?	?	?	?	?	?	?	?	?	?	?	?	?	?	?	?	?	?	?	?	?	?	0	1	1	0	?	0	?	?	0
Guizhoucoelacanthus	?	?	?	?	?	?	?	?	?	?	?	?	?	1	?	1	1	0	0	?	0	1	0	1	0	?	1	0	1	1	0	0	0	0	0
Hadronector	?	?	?	?	?	?	?	?	?	?	1	?	?	1	0	1	1	1	0	1	0	1	0	1	0	0	1	0	1	1	0	0	?	0	?
Heptanema	?	?	?	?	?	?	?	?	?	?	?	?	?	1	0	?	1	?	1	1	?	1	1	1	0	?	1	0	1	1	0	0	?	?	1
Holophagus	?	?	?	?	1	?	?	1	1	?	?	?	?	1	?	0	1	1	0	1	0	{01}	1	1	0	0	1	?	1	1	1	1	?	0	0
Holopterygius	0	0	?	?	?	?	?	?	?	?	?	?	?	1	0	0	?	0	0	0	?	?	?	0	?	?	1	1	0	1	?	0	?	1	?
Indocoelacanthus	?	?	?	?	?	?	?	?	?	?	0	?	?	?	?	?	?	?	1	?	0	?	?	?	?	0	?	?	?	1	?	?	?	?	0
Latimeria	0	1	0	1	1	0	0	1	1	1	1	1	0	1	0	0	1	1	0	0	0	1	1	1	0	0	1	0	1	1	0	0	1	0	0
Laugia	1	0	?	?	0	0	0	1	1	1	0	0	0	1	0	0	1	1	0	1	0	1	0	1	1	1	1	1	1	1	1	0	?	0	0
Libys	?	1	?	?	?	?	?	?	?	?	1	?	?	1	0	{01}	1	1	0	1	0	1	1	1	0	0	1	0	1	1	{01}	{01}	1	0	{01}
Lochmocercus	?	?	?	?	?	?	?	?	?	?	0	?	?	1	0	?	1	?	?	?	1	0	0	0	0	0	1	0	0	1	0	0	?	0	?
Lualabaea	?	?	?	?	?	?	?	?	?	?	0	?	?	?	?	?	?	?	?	?	?	0	1	?	?	?	?	?	?	?	?	?	?	?	1
Luopingcoelacanthus	?	1	?	?	?	?	?	?	1	?	?	?	?	1	0	?	1	0	0	1	0	0	1	1	0	0	1	0	1	1	0	0	?	0	1
Macropoma	0	1	?	1	1	0	?	1	1	1	1	?	1	1	1	0	1	1	0	1	0	1	1	1	0	0	1	0	1	1	0	0	1	0	1
Mawsonia	?	?	?	?	?	0	?	?	?	1	0	?	?	1	?	0	1	?	1	1	{01}	1	{01}	1	0	0	1	0	1	1	0	0	?	?	1
Megalocoelacanthus	0	1	?	?	?	0	?	1	?	1	1	1	1	1	?	?	?	1	?	?	?	?	?	?	?	?	?	?	?	?	?	?	?	?	?
Miguashaia	?	?	?	?	?	?	?	?	?	?	?	?	?	1	?	?	0	?	?	?	?	0	0	?	0	?	0	1	0	0	0	0	0	0	0
Ngamugawi	1	0	1	?	?	0	0	?	0	0	0	?	?	1	0	?	?	0	?	?	?	?	?	?	?	?	?	?	?	?	?	?	0	?	0
Parnaibaia	1	?	?	?	?	?	?	?	1	?	?	?	0	1	?	?	1	?	1	1	0	1	1	?	0	0	1	0	1	1	0	0	?	?	1
Piveteauia	1	1	?	?	?	?	?	?	?	?	?	?	?	1	?	0	?	1	0	1	0	1	0	1	1	?	1	1	1	1	0	0	?	0	0
Polyosteorhynchus	?	?	?	?	?	?	?	?	?	?	0	?	?	1	?	0	1	1	0	1	1	1	0	0	0	0	1	0	1	1	0	0	?	0	0
Rebellatrix	?	?	?	?	?	?	?	?	?	?	?	?	?	1	0	0	1	1	0	?	0	0	0	1	0	0	1	0	1	1	0	0	0	?	0
Reidus	?	?	?	?	?	?	?	?	?	?	?	?	?	?	?	?	?	?	?	?	?	?	?	?	?	?	?	?	?	?	?	?	?	?	?
Rhabdoderma	0	0	0	?	0	0	0	1	0	1	0	?	?	1	0	{01}	1	0	0	1	1	{01}	0	1	0	0	1	0	1	1	0	0	0	0	0
Rieppelia	0	1	?	?	?	?	?	?	?	?	?	?	?	1	0	1	1	?	0	0	?	0	1	?	0	0	1	1	0	1	0	0	?	0	0
Sassenia	?	0	1	?	0	0	1	1	0	1	0	?	?	1	?	?	?	?	?	?	?	?	?	?	?	?	?	?	?	?	?	?	?	0	0
Serenichthys	?	?	?	?	?	?	?	?	?	?	?	?	?	1	0	?	1	?	0	?	?	1	0	?	0	?	1	0	0	1	0	0	?	0	0
Spermatodus	1	0	?	?	0	0	?	1	0	1	?	?	?	?	?	?	?	?	?	?	?	?	?	?	?	?	?	?	?	?	?	?	?	?	0
Swenzia	?	1	?	?	?	?	?	?	?	?	?	?	?	?	?	0	1	?	0	1	0	?	?	1	0	?	1	0	1	?	?	?	?	?	0
Ticinepomis	0	1	?	?	1	?	?	1	1	?	?	?	0	1	0	1	1	?	0	0	0	1	1	1	0	0	1	0	1	1	0	0	?	0	{01}
Trachymetopon	?	?	0	?	0	0	?	1	?	1	0	?	?	1	?	0	1	1	1	1	0	0	1	1	0	0	1	0	1	1	0	0	1	0	1
Undina	0	1	?	?	1	?	?	1	1	1	1	?	0	1	0	0	1	?	0	1	0	1	1	1	0	0	1	0	1	1	{01}	{01}	1	0	0
Whiteia	0	0	?	1	1	0	1	1	1	1	0	0	?	1	?	1	1	0	0	{01}	0	1	{01}	1	0	0	1	0	1	1	0	0	1	0	0
Wimania	?	?	?	?	?	?	?	?	?	?	0	?	?	?	?	?	?	?	0	?	?	?	?	?	?	?	?	?	?	?	?	?	?	?	0
Yunnancoelacanthus	?	?	?	?	?	?	?	1	?	?	?	?	?	?	?	?	1	0	0	?	?	1	1	1	0	0	1	?	?	1	0	0	?	0	1

S2.4 List of apomorphies

Tree # 10

List of apomorphies:

Branch	Character	Steps	CI	Change
node_96 <-> Onychodontiformes (Onychodus)	64 (Coronoid fangs)	1	0.200	0 <=> 1
	74 (Temporal excavation)	1	0.333	1 <-> 0
	91 (Extracleithrum)	1	1.000	1 <=> 0
	110 (Lateral line openings in scales)	1	0.333	0 <=> 1
node_96 --> node_94	8 (parietal)	1	0.333	0 ==> 1
	14 (Intertemporal)	1	1.000	1 ==> 0
	22 (Supraorbital sensory canal)	1	1.000	0 ==> 1
	35 (Jugal)	1	1.000	0 ==> 1
	40 (Position of the preopercle within the cheek)	1	0.333	0 --> 1
	94 (Posterior neural and haemal spines)	1	0.500	0 --> 1
	107 (Fin ray)	1	1.000	0 ==> 1
node_94 --> node_56	23 (Supraorbital sensory canals opening as)	1	0.400	2 ==> 0
	62 (Number of anterior coronoids)	1	1.000	0 --> 1
	70 (Oral pit line)	1	1.000	0 ==> 1
	85 (Separate lateral ethmoids)	1	1.000	1 --> 0
node_56 --> node_53	7 (Internasal)	1	0.500	0 --> 1
	9 (Anterior and posterior pairs of parietals)	1	0.167	1 ==> 0
	78 (Buccohypophysial canal)	1	0.250	1 --> 0
node_53 --> node_51	2 (Parietonasal versus postparietal shield)	1	0.200	0 --> 1
	29 (Cheek bones)	1	0.250	0 --> 1
	33 (Postorbital)	1	0.200	0 --> 1
	52 (Infraorbital, jugal and preopercular sensory canals)	1	0.333	0 --> 1
	53 (Pit lines)	1	0.333	0 --> 1
	54 (Dermal bones of the cheek ornamented with)	1	0.333	0 --> 3
	98 (Basal plate of anterior dorsal fin)	1	0.333	1 --> 0
	105 (Caudal lobes)	1	0.167	0 ==> 1
	111 (Ventral keel scales)	1	1.000	0 ==> 1
node_51 --> Allenypterus	60 (Dentary teeth)	1	0.333	0 ==> 1
node_51 --> Holopterygius	97 (Ossified lung)	1	0.250	1 ==> 0
node_53 --> node_52	20 (Pair(s)_of_lateral_extrascapulars_(without_the_triple_junction_for_sensory_canals))	1	0.400	0 --> 1
node_52 --> Serenichthys	43 (Lachrymojugal)	1	0.333	0 ==> 1
	99 (Fin rays in the anterior dorsal fin)	1	0.200	0 ==> 1
node_56 --> node_55	12 (Number of supraorbitals/tectals)	1	0.333	0 --> 1
	37 (Squamosal)	1	0.250	0 ==> 1
	43 (Lachrymojugal)	1	0.333	0 --> 1
	49 (Infraorbital sensory canal)	1	0.200	1 ==> 0
	94 (Posterior neural and haemal spines)	1	0.500	1 --> 0
node_55 --> node_54	83 (Superficial ophthalmic branch of anterodorsal lateral line nerve)	1	1.000	0 ==> 1
node_54 --> Diplocercides	23 (Supraorbital sensory canals opening as)	1	0.400	0 ==> 2
node_55 --> Ngamugawi	24 (Medial branch of otic canal)	1	0.250	0 ==> 1
	30 (Spiracular (postspiracular))	1	0.200	1 ==> 0
	60 (Dentary teeth)	1	0.333	0 ==> 1
	65 (Prearticular and/or coronoid teeth)	1	0.200	0 ==> 1
	72 (Processus connectens)	1	0.250	1 ==> 0
node_94 --> node_93	11 (Parietal descending process)	1	1.000	0 --> 1
	16 (Supratemporal descending process)	1	0.500	0 ==> 1
	27 (Middle and posterior pit lines)	1	0.333	0 ==> 1
	60 (Dentary teeth)	1	0.333	0 ==> 1
	67 (Dentary sensory pore)	1	0.500	0 --> 1
	73 (Basipterygoid process)	1	1.000	1 --> 0
	81 (Vomers)	1	1.000	0 --> 1
	87 (Dorsum sellae)	1	1.000	0 --> 1
	101 (Basal support of the second dorsal fin)	1	0.500	0 --> 1
	106 (Fin rays)	1	0.333	0 ==> 1
node_93 --> node_91	7 (Internasal)	1	0.500	0 --> 1
	20 (Pair(s)_of_lateral_extrascapulars_(without_the_triple_junction_for_sensory_canals))	1	0.400	0 --> 1
	24 (Medial branch of otic canal)	1	0.250	0 --> 1
	29 (Cheek bones)	1	0.250	0 --> 1
	40 (Position of the preopercle within the cheek)	1	0.333	1 --> 0
	63 (Coronoid)	1	0.167	0 ==> 1
node_91 --> node_90	71 (Orbitosphenoid and basisphenoid regions)	1	1.000	0 ==> 1
	80 (Suprapterygoid process)	1	1.000	1 ==> 0
node_90 --> node_87	5 (Premaxilla)	1	0.200	0 ==> 1
	6 (Anterior opening of the rostral organ)	1	0.333	0 --> 1
	17 (Posterior margin of the skull roof)	1	0.333	0 ==> 1
	41 (Subopercle)	1	0.200	1 --> 0
	47 (Posterior opening(s) of the rostral organ mark(s) bone as)	1	0.500	0 --> 1
	48 (Anterior and/or posterior branches of the infraorbital canal within the postorbital)	1	0.333	0 --> 1
	49 (Infraorbital sensory canal)	1	0.200	1 ==> 0
	53 (Pit lines)	1	0.333	0 --> 1
	75 (Otico-occipital)	1	1.000	0 --> 1
	86 (Separate basioccipital)	1	1.000	0 --> 1
	98 (Basal plate of anterior dorsal fin)	1	0.333	1 ==> 0
node_87 --> node_86	57 (Retroarticular and articular)	1	0.500	0 ==> 1
	95 (Occipital neural arches)	1	0.333	0 ==> 1
node_86 --> node_85	99 (Fin rays in the anterior dorsal fin)	1	0.200	0 ==> 1
	110 (Lateral line openings in scales)	1	0.333	0 ==> 1
node_85 --> node_82	2 (Parietonasal versus postparietal shield)	1	0.200	0 --> 1
	9 (Anterior and posterior pairs of parietals)	1	0.167	1 --> 0
	18 (Extrascapulars)	1	0.200	0 --> 1
	40 (Position of the preopercle within the cheek)	1	0.333	0 ==> 1
	42 (Lachrymojugal)	1	1.000	0 ==> 1
	72 (Processus connectens)	1	0.250	1 ==> 0
	76 (Supraoccipital)	1	1.000	0 --> 1
	78 (Buccohypophysial canal)	1	0.250	1 ==> 0
	82 (Prootic)	1	0.500	0 ==> 1
node_82 --> node_79	20 (Pair(s)_of_lateral_extrascapulars_(without_the_triple_junction_for_sensory_canals))	1	0.400	1 ==> 2
	48 (Anterior and/or posterior branches of the infraorbital canal within the postorbital)	1	0.333	1 --> 0
	89 (Basibranchial tooth plates)	1	1.000	0 --> 1
	100 (Anterior dorsal fin)	1	0.500	0 ==> 1
node_79 --> node_58	2 (Parietonasal versus postparietal shield)	1	0.200	1 --> 0
	8 (parietal)	1	0.333	1 ==> 0
	21 (Median extrascapular)	1	0.333	0 --> 1
	34 (Postorbital)	1	0.500	0 --> 1
node_58 --> Atacamaia	10 (Parietals and postparietals)	1	0.333	0 ==> 1
	33 (Postorbital)	1	0.200	0 ==> 1

node_58 --> node_57	23 (Supraorbital sensory canals opening as)	1	0.400	2 --> 0
	55 (Orbital space)	1	0.333	0 --> 1
node_57 --> Axelia	65 (Prearticular and/or coronoid teeth)	1	0.200	0 ==> 1
node_79 --> node_78	13 (Preorbital)	1	0.250	1 --> 0
	15 (Postparietal descending process)	1	0.500	0 ==> 1
	26 (Pit lines)	1	0.167	0 ==> 1
	46 (Posterior opening of the rostral organ marks)	1	0.500	0 --> 1
	56 (Sclerotic ossicles)	1	0.333	1 ==> 0
	58 (Dentary)	1	0.500	0 ==> 1
	63 (Coronoid)	1	0.167	1 --> 0
node_78 --> node_67	16 (Supratemporal descending process)	1	0.500	1 --> 0
	27 (Middle and posterior pit lines)	1	0.333	1 --> 0
	28 (Dermal bones of the skull ornamented with)	1	0.333	0 ==> 2
	54 (Dermal bones of the cheek ornamented with)	1	0.333	0 ==> 2
	59 (Dentary)	1	0.333	0 ==> 1
	68 (Mandibular sensory canal on the splenial)	1	0.500	0 --> 1
	96 (Ossified ribs)	1	1.000	0 ==> 1
node_67 --> node_66	112 (Scale ornament)	1	0.333	0 ==> 1
node_66 --> node_63	6 (Anterior opening of the rostral organ)	1	0.333	1 --> 0
	18 (Extrascapulars)	1	0.200	1 ==> 0
	19 (Extrascapulars)	1	1.000	0 ==> 1
	30 (Spiracular (postspiracular))	1	0.200	1 --> 0
	34 (Postorbital)	1	0.500	0 ==> 1
	55 (Orbital space)	1	0.333	0 ==> 1
	69 (Oral pit line)	1	0.250	0 ==> 1
node_63 --> node_62	61 (Principal coronoid)	1	1.000	0 --> 1
node_62 --> node_61	4 (Premaxillary teeth)	1	0.250	0 --> 1
	9 (Anterior and posterior pairs of parietals)	1	0.167	0 --> 1
	20 (Pair(s)_of_lateral_extrascapulars_(without_the_triple_junction_for_sensory_canals))	1	0.400	2 ==> 1
	32 (Postorbital)	1	1.000	0 ==> 1
	65 (Prearticular and/or coronoid teeth)	1	0.200	0 --> 1
node_61 --> Axelrodichthys	24 (Medial branch of otic canal)	1	0.250	1 ==> 0
node_61 --> node_60	44 (contact between the lachrymojugal and the preorbital or tectal-supraorbital series)	1	0.500	0 --> 1
	72 (Processus connectens)	1	0.250	0 ==> 1
	82 (Prootic)	1	0.500	1 --> 0
node_60 --> node_59	55 (Orbital space)	1	0.333	1 --> 0
	68 (Mandibular sensory canal on the splenial)	1	0.500	1 --> 0
	99 (Fin rays in the anterior dorsal fin)	1	0.200	1 ==> 0
node_59 --> Trachymetopon	59 (Dentary)	1	0.333	1 ==> 0
node_60 --> Mawsonia	21 (Median extrascapular)	1	0.333	0 ==> 1
node_62 --> Parnaibaia	21 (Median extrascapular)	1	0.333	0 ==> 1
	26 (Pit lines)	1	0.167	1 ==> 0
	78 (Buccohypophysial canal)	1	0.250	0 ==> 1
node_63 --> Chinlea	59 (Dentary)	1	0.333	1 ==> 0
	63 (Coronoid)	1	0.167	0 --> 1
	64 (Coronoid fangs)	1	0.200	0 ==> 1
	100 (Anterior dorsal fin)	1	0.500	1 ==> 0
node_66 --> node_65	23 (Supraorbital sensory canals opening as)	1	0.400	2 --> 0
	28 (Dermal bones of the skull ornamented with)	1	0.333	2 ==> 3
	33 (Postorbital)	1	0.200	0 --> 1
	52 (Infraorbital, jugal and preopercular sensory canals)	1	0.333	0 --> 1
	54 (Dermal bones of the cheek ornamented with)	1	0.333	2 ==> 3
node_65 --> node_64	9 (Anterior and posterior pairs of parietals)	1	0.167	0 --> 1
	97 (Ossified lung)	1	0.250	1 --> 0
node_64 --> Diplurus	36 (Squamosal)	1	0.250	0 ==> 1
	38 (Preopercle)	1	0.500	0 ==> 1
node_78 --> node_77	4 (Premaxillary teeth)	1	0.250	0 --> 1
	30 (Spiracular (postspiracular))	1	0.200	1 --> 0
	41 (Subopercle)	1	0.200	0 ==> 1
	45 (Posterior nostril on the lachrymojugal)	1	0.500	0 --> 1
	64 (Coronoid fangs)	1	0.200	0 --> 1
	66 (Subopercular branch of the mandibular sensory canal)	1	1.000	0 --> 1
	74 (Temporal excavation)	1	0.333	1 ==> 0
	79 (Parasphenoid)	1	0.500	0 --> 1
node_77 --> Dobrogeria	72 (Processus connectens)	1	0.250	0 ==> 1
node_77 --> node_76	88 (Ventral swelling of the palatoquadrate)	1	0.500	0 ==> 1
node_76 --> node_69	13 (Preorbital)	1	0.250	0 --> 1
	17 (Posterior margin of the skull roof)	1	0.333	1 ==> 0
	20 (Pair(s)_of_lateral_extrascapulars_(without_the_triple_junction_for_sensory_canals))	1	0.400	2 --> 0
	24 (Medial branch of otic canal)	1	0.250	1 --> 0
	33 (Postorbital)	1	0.200	0 ==> 1
	36 (Squamosal)	1	0.250	0 --> 1
	43 (Lachrymojugal)	1	0.333	0 ==> 1
	46 (Posterior opening of the rostral organ marks)	1	0.500	1 --> 0
	52 (Infraorbital, jugal and preopercular sensory canals)	1	0.333	0 --> 1
	93 (Number of neural arches)	1	0.333	0 ==> 1
	97 (Ossified lung)	1	0.250	1 ==> 0
node_69 --> node_68	1 (Parietonasal and postparietal shields)	1	1.000	0 ==> 1
	2 (Parietonasal versus postparietal shield)	1	0.200	1 ==> 0
	18 (Extrascapulars)	1	0.200	1 --> 0
	23 (Supraorbital sensory canals opening as)	1	0.400	2 --> 0
	28 (Dermal bones of the skull ornamented with)	1	0.333	0 ==> 1
	54 (Dermal bones of the cheek ornamented with)	1	0.333	0 ==> 1
	99 (Fin rays in the anterior dorsal fin)	1	0.200	1 ==> 0
node_68 --> Foreyia	23 (Supraorbital sensory canals opening as)	1	0.400	0 --> 4
	31 (Postorbital)	1	0.333	0 ==> 1
	45 (Posterior nostril on the lachrymojugal)	1	0.500	1 ==> 0
	52 (Infraorbital, jugal and preopercular sensory canals)	1	0.333	1 --> 0
	112 (Scale ornament)	1	0.333	0 ==> 1
node_68 --> Rieppelia	5 (Premaxilla)	1	0.200	1 ==> 0
	29 (Cheek bones)	1	0.250	1 ==> 0
	41 (Subopercle)	1	0.200	1 ==> 0
	46 (Posterior opening of the rostral organ marks)	1	0.500	0 --> 2
	47 (Posterior opening(s) of the rostral organ mark(s) bone as)	1	0.500	1 ==> 2
	105 (Caudal lobes)	1	0.167	0 ==> 1
	106 (Fin rays)	1	0.333	1 ==> 0
node_76 --> node_75	9 (Anterior and posterior pairs of parietals)	1	0.167	0 --> 1
	12 (Number of supraorbitals/tectals)	1	0.333	0 ==> 1
	25 (Anterior branches of supratemporal commissure)	1	1.000	0 --> 1
	28 (Dermal bones of the skull ornamented with)	1	0.333	0 ==> 3
	48 (Anterior and/or posterior branches of the infraorbital canal within the postorbital)	1	0.333	0 --> 1
	49 (Infraorbital sensory canal)	1	0.200	0 ==> 1
	51 (Jugal sensory canal)	1	0.500	0 ==> 1
	63 (Coronoid)	1	0.167	0 --> 1
	77 (Toothed area of the parasphenoid)	1	1.000	0 ==> 1

node_75 -->	node_74	54 (Dermal bones of the cheek ornamented with)	1	0.333	0 ==> 1
node_74 -->	Holophagus	108 (Paired fin rays)	1	0.500	0 ==> 1
		109 (Median fin rays)	1	1.000	0 ==> 1
node_74 -->	node_73	3 (Snout bones)	1	0.333	0 --> 1
		23 (Supraorbital sensory canals opening as)	1	0.400	2 --> 0
		31 (Postorbital)	1	0.333	0 --> 1
		39 (Preopercle)	1	1.000	0 ==> 1
		44 (contact between the lachrymojugal and the preorbital or tectal-supraorbital series)	1	0.500	0 ==> 1
node_73 -->	node_70	10 (Parietals and postparietals)	1	0.333	0 ==> 1
		30 (Spiracular (postspiracular))	1	0.200	0 ==> 1
node_70 -->	Latimeria	3 (Snout bones)	1	0.333	1 --> 0
		46 (Posterior opening of the rostral organ marks)	1	0.500	1 ==> 2
		47 (Posterior opening(s) of the rostral organ mark(s) bone as)	1	0.500	1 ==> 2
		50 (Prominent branches of the jugal sensory canal within the squamosal)	1	0.250	0 ==> 1
		52 (Infraorbital, jugal and preopercular sensory canals)	1	0.333	0 ==> 1
		97 (Ossified lung)	1	0.250	1 ==> 0
node_70 -->	Swenzia	26 (Pit lines)	1	0.167	1 ==> 0
node_73 -->	node_72	5 (Premaxilla)	1	0.200	1 --> 0
		41 (Subopercle)	1	0.200	1 --> 0
		64 (Coronoid fangs)	1	0.200	1 ==> 0
		90 (Anterior basibranchial tooth plates)	1	1.000	0 ==> 1
		112 (Scale ornament)	1	0.333	0 --> 1
node_72 -->	node_71	23 (Supraorbital sensory canals opening as)	1	0.400	0 ==> 3
		31 (Postorbital)	1	0.333	1 --> 0
		52 (Infraorbital, jugal and preopercular sensory canals)	1	0.333	0 ==> 2
		54 (Dermal bones of the cheek ornamented with)	1	0.333	1 ==> 3
		56 (Sclerotic ossicles)	1	0.333	0 --> 1
		65 (Prearticular and/or coronoid teeth)	1	0.200	0 ==> 1
		74 (Temporal excavation)	1	0.333	0 --> 1
node_71 -->	Megalocoelacanthus	63 (Coronoid)	1	0.167	1 ==> 0
		69 (Oral pit line)	1	0.250	0 ==> 1
node_72 -->	Macropoma	28 (Dermal bones of the skull ornamented with)	1	0.333	3 ==> 1
		92 (Anocleithrum)	1	0.500	0 ==> 1
node_75 -->	Undina	5 (Premaxilla)	1	0.200	1 ==> 0
		10 (Parietals and postparietals)	1	0.333	0 ==> 1
node_82 -->	node_81	5 (Premaxilla)	1	0.200	1 --> 0
		6 (Anterior opening of the rostral organ)	1	0.333	1 --> 0
		28 (Dermal bones of the skull ornamented with)	1	0.333	0 ==> 3
		50 (Prominent branches of the jugal sensory canal within the squamosal)	1	0.250	0 --> 1
		53 (Pit lines)	1	0.333	1 --> 0
		84 (Process on braincase for articulation of infrabranchial 1)	1	0.500	0 --> 1
		93 (Number of neural arches)	1	0.333	0 ==> 1
		95 (Occipital neural arches)	1	0.333	1 ==> 0
node_81 -->	node_80	18 (Extrascapulars)	1	0.200	1 --> 0
		27 (Middle and posterior pit lines)	1	0.333	1 --> 0
		54 (Dermal bones of the cheek ornamented with)	1	0.333	0 ==> 3
		56 (Sclerotic ossicles)	1	0.333	1 ==> 0
		110 (Lateral line openings in scales)	1	0.333	1 --> 0
node_80 -->	Garnbergia	13 (Preorbital)	1	0.250	1 ==> 0
node_81 -->	Whiteia	41 (Subopercle)	1	0.200	0 ==> 1
		47 (Posterior opening(s) of the rostral organ mark(s) bone as)	1	0.500	1 ==> 0
		49 (Infraorbital sensory canal)	1	0.200	0 ==> 1
		58 (Dentary)	1	0.500	0 ==> 1
node_85 -->	node_84	3 (Snout bones)	1	0.333	0 --> 1
		38 (Preopercle)	1	0.500	0 --> 1
		102 (Pelvics)	1	1.000	0 ==> 1
		103 (Pelvic bones of each side)	1	1.000	0 --> 1
		105 (Caudal lobes)	1	0.167	0 --> 1
node_84 -->	node_83	17 (Posterior margin of the skull roof)	1	0.333	1 ==> 0
		30 (Spiracular (postspiracular))	1	0.200	1 ==> 0
		51 (Jugal sensory canal)	1	0.500	0 ==> 1
		57 (Retroarticular and articular)	1	0.500	1 --> 0
		108 (Paired fin rays)	1	0.500	0 ==> 1
node_83 -->	Coccoderma	28 (Dermal bones of the skull ornamented with)	1	0.333	0 ==> 3
		36 (Squamosal)	1	0.250	0 ==> 1
		54 (Dermal bones of the cheek ornamented with)	1	0.333	0 ==> 3
		92 (Anocleithrum)	1	0.500	0 ==> 1
		105 (Caudal lobes)	1	0.167	1 --> 0
node_83 -->	Laugia	8 (parietal)	1	0.333	1 ==> 0
		26 (Pit lines)	1	0.167	0 ==> 1
		63 (Coronoid)	1	0.167	1 ==> 0
node_84 -->	Piveteauia	15 (Postparietal descending process)	1	0.500	0 ==> 1
		50 (Prominent branches of the jugal sensory canal within the squamosal)	1	0.250	0 ==> 1
		79 (Parasphenoid)	1	0.500	0 ==> 1
node_87 -->	Coelacanthus	13 (Preorbital)	1	0.250	1 ==> 0
		26 (Pit lines)	1	0.167	0 ==> 1
		28 (Dermal bones of the skull ornamented with)	1	0.333	0 ==> 3
		33 (Postorbital)	1	0.200	0 ==> 1
		36 (Squamosal)	1	0.250	0 ==> 1
		69 (Oral pit line)	1	0.250	0 ==> 1
node_90 -->	node_89	29 (Cheek bones)	1	0.250	1 --> 0
		64 (Coronoid fangs)	1	0.200	0 ==> 1
node_89 -->	node_88	4 (Premaxillary teeth)	1	0.250	0 --> 1
		23 (Supraorbital sensory canals opening as)	1	0.400	2 ==> 0
		78 (Buccohypophysial canal)	1	0.250	1 --> 0
node_88 -->	Caridosuctor	12 (Number of supraorbitals/tectals)	1	0.333	0 ==> 1
		105 (Caudal lobes)	1	0.167	0 ==> 1
node_88 -->	Rhabdoderma	9 (Anterior and posterior pairs of parietals)	1	0.167	1 ==> 0
		50 (Prominent branches of the jugal sensory canal within the squamosal)	1	0.250	0 ==> 1
node_89 -->	Spermatodus	18 (Extrascapulars)	1	0.200	0 ==> 1
		26 (Pit lines)	1	0.167	0 ==> 1
		37 (Squamosal)	1	0.250	0 ==> 1
		65 (Prearticular and/or coronoid teeth)	1	0.200	0 ==> 1
node_91 -->	Sassenia	28 (Dermal bones of the skull ornamented with)	1	0.333	0 ==> 1
		37 (Squamosal)	1	0.250	0 ==> 1
		54 (Dermal bones of the cheek ornamented with)	1	0.333	0 ==> 1
		69 (Oral pit line)	1	0.250	0 ==> 1
		84 (Process on braincase for articulation of infrabranchial 1)	1	0.500	0 ==> 1

node_93 --> node_92	2 (Parietonasal versus postparietal shield)	1	0.200	0 ==> 1
	23 (Supraorbital sensory canals opening as)	1	0.400	2 ==> 1
	95 (Occipital neural arches)	1	0.333	0 ==> 1
	99 (Fin rays in the anterior dorsal fin)	1	0.200	0 ==> 1
node_92 --> Hadronector	67 (Dentary sensory pore)	1	0.500	1 --> 0
	88 (Ventral swelling of the palatoquadrate)	1	0.500	0 ==> 1
	93 (Number of neural arches)	1	0.333	0 ==> 1
	98 (Basal plate of anterior dorsal fin)	1	0.333	1 ==> 0
node_92 --> Polyosteorhynchus	4 (Premaxillary teeth)	1	0.250	0 ==> 1
	101 (Basal support of the second dorsal fin)	1	0.500	1 --> 0
node_96 --> node_95	37 (Squamosal)	1	0.250	0 ==> 1
	49 (Infraorbital sensory canal)	1	0.200	1 ==> 0
	104 (Diphycercal tail)	1	1.000	1 ==> 0
	105 (Caudal lobes)	1	0.167	0 --> 1
node_95 --> Gavinia	106 (Fin rays)	1	0.333	0 ==> 1

S2.5 List of character changes

Tree # 10

List of character changes:

Character	CI	Steps	Changes
1 (Parietonasal and postparietal shields)	1.000	1	node_69 0 ==> 1 node_68
2 (Parietonasal versus postparietal shield)	0.200	1	node_53 0 --> 1 node_51
		1	node_85 0 --> 1 node_82
		1	node_79 1 --> 0 node_58
		1	node_69 1 ==> 0 node_68
		1	node_93 0 ==> 1 node_92
3 (Snout bones)	0.333	1	node_74 0 --> 1 node_73
		1	node_70 1 --> 0 Latimeria
		1	node_85 0 --> 1 node_84
4 (Premaxillary teeth)	0.250	1	node_62 0 --> 1 node_61
		1	node_78 0 --> 1 node_77
		1	node_89 0 --> 1 node_88
		1	node_92 0 ==> 1 Polyosteorhynchus
5 (Premaxilla)	0.200	1	node_90 0 ==> 1 node_87
		1	node_68 1 ==> 0 Rieppelia
		1	node_73 1 --> 0 node_72
		1	node_75 1 ==> 0 Undina
		1	node_82 1 --> 0 node_81
6 (Anterior opening of the rostral organ)	0.333	1	node_90 0 --> 1 node_87
		1	node_66 1 --> 0 node_63
		1	node_82 1 --> 0 node_81
7 (Internasal)	0.500	1	node_56 0 --> 1 node_53
		1	node_93 0 --> 1 node_91
8 (parietal)	0.333	1	node_96 0 ==> 1 node_94
		1	node_79 1 ==> 0 node_58
		1	node_83 1 ==> 0 Laugia
9 (Anterior and posterior pairs of parietals)	0.167	1	node_56 1 ==> 0 node_53
		1	node_85 1 --> 0 node_82
		1	node_62 0 --> 1 node_61
		1	node_65 0 --> 1 node_64
		1	node_76 0 --> 1 node_75
		1	node_88 1 ==> 0 Rhabdoderma
10 (Parietals and postparietals)	0.333	1	node_58 0 ==> 1 Atacamaia
		1	node_73 0 ==> 1 node_70
		1	node_75 0 ==> 1 Undina
11 (Parietal descending process)	1.000	1	node_94 0 --> 1 node_93
12 (Number of supraorbitals/tectals)	0.333	1	node_56 0 --> 1 node_55
		1	node_76 0 ==> 1 node_75
		1	node_88 0 ==> 1 Caridosuctor
13 (Preorbital)	0.250	1	node_79 1 --> 0 node_78
		1	node_76 0 --> 1 node_69
		1	node_80 1 ==> 0 Garnbergia
		1	node_87 1 ==> 0 Coelacanthus
14 (Intertemporal)	1.000	1	node_96 1 ==> 0 node_94
15 (Postparietal descending process)	0.500	1	node_79 0 ==> 1 node_78
		1	node_84 0 ==> 1 Piveteauia
16 (Supratemporal descending process)	0.500	1	node_94 0 ==> 1 node_93
		1	node_78 1 --> 0 node_67
17 (Posterior margin of the skull roof)	0.333	1	node_90 0 ==> 1 node_87
		1	node_76 1 ==> 0 node_69
		1	node_84 1 ==> 0 node_83
18 (Extrascapulars)	0.200	1	node_85 0 --> 1 node_82
		1	node_66 1 ==> 0 node_63
		1	node_69 1 --> 0 node_68
		1	node_81 1 --> 0 node_80
		1	node_89 0 ==> 1 Spermatodus
19 (Extrascapulars)	1.000	1	node_66 0 ==> 1 node_63
20 (Pair(s)_of_lateral_extrascapulars_(without_the_triple_junction_for_sensory_canals))	0.400	1	node_53 0 --> 1 node_52
		1	node_93 0 --> 1 node_91
		1	node_82 1 ==> 2 node_79
		1	node_62 2 ==> 1 node_61
		1	node_76 2 --> 0 node_69
21 (Median extrascapular)	0.333	1	node_79 0 --> 1 node_58
		1	node_60 0 ==> 1 Mawsonia
		1	node_62 0 ==> 1 Parnaibaia
22 (Supraorbital sensory canal)	1.000	1	node_96 0 ==> 1 node_94
23 (Supraorbital sensory canals opening as)	0.400	1	node_94 2 ==> 0 node_56
		1	node_54 0 ==> 2 Diplocercides
		1	node_58 2 --> 0 node_57
		1	node_66 2 --> 0 node_65
		1	node_69 2 --> 0 node_68
		1	node_68 0 --> 4 Foreyia
		1	node_74 2 --> 0 node_73
		1	node_72 0 ==> 3 node_71
		1	node_89 2 ==> 0 node_88
		1	node_93 2 ==> 1 node_92
24 (Medial branch of otic canal)	0.250	1	node_55 0 ==> 1 Ngamugawi
		1	node_93 0 --> 1 node_91
		1	node_61 1 ==> 0 Axelrodichthys
		1	node_76 1 --> 0 node_69
25 (Anterior branches of supratemporal commissure)	1.000	1	node_76 0 --> 1 node_75
26 (Pit lines)	0.167	1	node_79 0 ==> 1 node_78
		1	node_62 1 ==> 0 Parnaibaia
		1	node_70 1 ==> 0 Swenzia
		1	node_83 0 ==> 1 Laugia
		1	node_87 0 ==> 1 Coelacanthus
		1	node_89 0 ==> 1 Spermatodus

Character	CI	Steps	Change
27 (Middle and posterior pit lines)	0.333	1	node_94 0 ==> 1 node_93
		1	node_78 1 --> 0 node_67
		1	node_81 1 --> 0 node_80
28 (Dermal bones of the skull ornamented with)	0.333	1	node_78 0 ==> 2 node_67
		1	node_66 2 ==> 3 node_65
		1	node_69 0 ==> 1 node_68
		1	node_76 0 ==> 3 node_75
		1	node_72 3 ==> 1 Macropoma
		1	node_82 0 ==> 3 node_81
		1	node_83 0 ==> 3 Coccoderma
		1	node_87 0 ==> 3 Coelacanthus
		1	node_91 0 ==> 1 Sassenia
29 (Cheek bones)	0.250	1	node_53 0 --> 1 node_51
		1	node_93 0 --> 1 node_91
		1	node_68 1 ==> 0 Rieppelia
		1	node_90 1 --> 0 node_89
30 (Spiracular (postspiracular))	0.200	1	node_55 1 ==> 0 Ngamugawi
		1	node_66 1 --> 0 node_63
		1	node_78 1 --> 0 node_77
		1	node_73 0 ==> 1 node_70
		1	node_84 1 ==> 0 node_83
31 (Postorbital)	0.333	1	node_68 0 ==> 1 Foreyia
		1	node_74 0 --> 1 node_73
		1	node_72 1 --> 0 node_71
32 (Postorbital)	1.000	1	node_62 0 ==> 1 node_61
33 (Postorbital)	0.200	1	node_53 0 --> 1 node_51
		1	node_58 0 ==> 1 Atacamaia
		1	node_66 0 --> 1 node_65
		1	node_76 0 ==> 1 node_69
		1	node_87 0 ==> 1 Coelacanthus
34 (Postorbital)	0.500	1	node_79 0 --> 1 node_58
		1	node_66 0 ==> 1 node_63
35 (Jugal)	1.000	1	node_96 0 ==> 1 node_94
36 (Squamosal)	0.250	1	node_64 0 ==> 1 Diplurus
		1	node_76 0 --> 1 node_69
		1	node_83 0 ==> 1 Coccoderma
		1	node_87 0 ==> 1 Coelacanthus
37 (Squamosal)	0.250	1	node_56 0 ==> 1 node_55
		1	node_89 0 ==> 1 Spermatodus
		1	node_91 0 ==> 1 Sassenia
		1	node_96 0 ==> 1 node_95
38 (Preopercle)	0.500	1	node_64 0 ==> 1 Diplurus
		1	node_85 0 --> 1 node_84
39 (Preopercle)	1.000	1	node_74 0 ==> 1 node_73
40 (Position of the preopercle within the cheek)	0.333	1	node_96 0 --> 1 node_94
		1	node_93 1 --> 0 node_91
		1	node_85 0 ==> 1 node_82
41 (Subopercle)	0.200	1	node_90 1 --> 0 node_87
		1	node_78 0 ==> 1 node_77
		1	node_68 1 ==> 0 Rieppelia
		1	node_73 1 --> 0 node_72
		1	node_81 0 ==> 1 Whiteia
42 (Lachrymojugal)	1.000	1	node_85 0 ==> 1 node_82
43 (Lachrymojugal)	0.333	1	node_52 0 ==> 1 Serenichthys
		1	node_56 0 --> 1 node_55
		1	node_76 0 ==> 1 node_69
44 (contact between the lachrymojugal and the preorbital or tectal-supraorbital series)	0.500	1	node_61 0 --> 1 node_60
		1	node_74 0 ==> 1 node_73
45 (Posterior nostril on the lachrymojugal)	0.500	1	node_78 0 --> 1 node_77
		1	node_68 1 ==> 0 Foreyia
46 (Posterior opening of the rostral organ marks)	0.500	1	node_79 0 --> 1 node_78
		1	node_76 1 --> 0 node_69
		1	node_68 0 --> 2 Rieppelia
		1	node_70 1 ==> 2 Latimeria
47 (Posterior opening(s) of the rostral organ mark(s) bone as)	0.500	1	node_90 0 --> 1 node_87
		1	node_68 1 ==> 2 Rieppelia
		1	node_70 1 ==> 2 Latimeria
		1	node_81 1 ==> 0 Whiteia
48 (Anterior and/or posterior branches of the infraorbital canal within the postorbital)	0.333	1	node_90 0 --> 1 node_87
		1	node_82 1 --> 0 node_79
		1	node_76 0 --> 1 node_75
49 (Infraorbital sensory canal)	0.200	1	node_56 1 ==> 0 node_55
		1	node_90 1 ==> 0 node_87
		1	node_76 0 ==> 1 node_75
		1	node_81 0 ==> 1 Whiteia
		1	node_96 1 ==> 0 node_95
50 (Prominent branches of the jugal sensory canal within the squamosal)	0.250	1	node_70 0 ==> 1 Latimeria
		1	node_82 0 --> 1 node_81
		1	node_84 0 ==> 1 Piveteauia
		1	node_88 0 ==> 1 Rhabdoderma
51 (Jugal sensory canal)	0.500	1	node_76 0 ==> 1 node_75
		1	node_84 0 ==> 1 node_83
52 (Infraorbital, jugal and preopercular sensory canals)	0.333	1	node_53 0 --> 1 node_51
		1	node_66 0 --> 1 node_65
		1	node_76 0 --> 1 node_69
		1	node_68 1 --> 0 Foreyia
		1	node_70 0 ==> 1 Latimeria
		1	node_72 0 ==> 2 node_71
53 (Pit lines)	0.333	1	node_53 0 --> 1 node_51
		1	node_90 0 --> 1 node_87
		1	node_82 1 --> 0 node_81

54 (Dermal bones of the cheek ornamented with)	0.333	1	node_53 0 --> 3 node_51
		1	node_78 0 ==> 2 node_67
		1	node_66 2 ==> 3 node_65
		1	node_69 0 ==> 1 node_68
		1	node_75 0 ==> 1 node_74
		1	node_72 1 ==> 3 node_71
		1	node_81 0 ==> 3 node_80
		1	node_83 0 ==> 3 Coccoderma
		1	node_91 0 ==> 1 Sassenia
55 (Orbital space)	0.333	1	node_58 0 --> 1 node_57
		1	node_66 0 ==> 1 node_63
		1	node_60 1 --> 0 node_59
56 (Sclerotic ossicles)	0.333	1	node_79 1 ==> 0 node_78
		1	node_72 0 --> 1 node_71
		1	node_81 1 ==> 0 node_80
57 (Retroarticular and articular)	0.500	1	node_87 0 ==> 1 node_86
		1	node_84 1 --> 0 node_83
58 (Dentary)	0.500	1	node_79 0 ==> 1 node_78
		1	node_81 0 ==> 1 Whiteia
59 (Dentary)	0.333	1	node_78 0 ==> 1 node_67
		1	node_59 1 ==> 0 Trachymetopon
		1	node_63 1 ==> 0 Chinlea
60 (Dentary teeth)	0.333	1	node_51 0 ==> 1 Allenypterus
		1	node_55 0 ==> 1 Ngamugawi
		1	node_94 0 ==> 1 node_93
61 (Principal coronoid)	1.000	1	node_63 0 --> 1 node_62
62 (Number of anterior coronoids)	1.000	1	node_94 0 --> 1 node_56
63 (Coronoid)	0.167	1	node_93 0 ==> 1 node_91
		1	node_79 1 --> 0 node_78
		1	node_63 0 --> 1 Chinlea
		1	node_76 0 --> 1 node_75
		1	node_71 1 ==> 0 Megalocoelacanthus
		1	node_83 1 ==> 0 Laugia
64 (Coronoid fangs)	0.200	1	Onychod 1 <=> 0 node_96
		1	node_63 0 ==> 1 Chinlea
		1	node_78 0 --> 1 node_77
		1	node_73 1 ==> 0 node_72
		1	node_90 0 ==> 1 node_89
65 (Prearticular and/or coronoid teeth)	0.200	1	node_55 0 ==> 1 Ngamugawi
		1	node_57 0 ==> 1 Axelia
		1	node_62 0 --> 1 node_61
		1	node_72 0 ==> 1 node_71
		1	node_89 0 ==> 1 Spermatodus
66 (Subopercular branch of the mandibular sensory canal)	1.000	1	node_78 0 --> 1 node_77
67 (Dentary sensory pore)	0.500	1	node_94 0 --> 1 node_93
		1	node_92 1 --> 0 Hadronector
68 (Mandibular sensory canal on the splenial)	0.500	1	node_78 0 --> 1 node_67
		1	node_60 1 --> 0 node_59
69 (Oral pit line)	0.250	1	node_66 0 ==> 1 node_63
		1	node_71 0 ==> 1 Megalocoelacanthus
		1	node_87 0 ==> 1 Coelacanthus
		1	node_91 0 ==> 1 Sassenia
70 (Oral pit line)	1.000	1	node_94 0 ==> 1 node_56
71 (Orbitosphenoid and basisphenoid regions)	1.000	1	node_91 0 ==> 1 node_90
72 (Processus connectens)	0.250	1	node_55 1 ==> 0 Ngamugawi
		1	node_85 1 ==> 0 node_82
		1	node_61 0 ==> 1 node_60
		1	node_77 0 ==> 1 Dobrogeria
73 (Basipterygoid process)	1.000	1	node_94 1 --> 0 node_93
74 (Temporal excavation)	0.333	1	Onychod 0 <-> 1 node_96
		1	node_78 1 ==> 0 node_77
		1	node_72 0 --> 1 node_71
75 (Otico-occipital)	1.000	1	node_90 0 --> 1 node_87
76 (Supraoccipital)	1.000	1	node_85 0 --> 1 node_82
77 (Toothed area of the parasphenoid)	1.000	1	node_76 0 ==> 1 node_75
78 (Buccohypophysial canal)	0.250	1	node_56 1 --> 0 node_53
		1	node_85 1 ==> 0 node_82
		1	node_62 0 ==> 1 Parnaibaia
		1	node_89 1 --> 0 node_88
79 (Parasphenoid)	0.500	1	node_78 0 --> 1 node_77
		1	node_84 0 ==> 1 Piveteauia
80 (Suprapterygoid process)	1.000	1	node_91 1 ==> 0 node_90
81 (Vomers)	1.000	1	node_94 0 --> 1 node_93
82 (Prootic)	0.500	1	node_85 0 ==> 1 node_82
		1	node_61 1 --> 0 node_60
83 (Superficial ophthalmic branch of anterodorsal lateral line nerve)	1.000	1	node_55 0 ==> 1 node_54
84 (Process on braincase for articulation of infrabranchial 1)	0.500	1	node_82 0 --> 1 node_81
		1	node_91 0 ==> 1 Sassenia
85 (Separate lateral ethmoids)	1.000	1	node_94 1 --> 0 node_56
86 (Separate basioccipital)	1.000	1	node_90 0 --> 1 node_87
87 (Dorsum sellae)	1.000	1	node_94 0 --> 1 node_93
88 (Ventral swelling of the palatoquadrate)	0.500	1	node_77 0 ==> 1 node_76
		1	node_92 0 ==> 1 Hadronector
89 (Basibranchial tooth plates)	1.000	1	node_82 0 --> 1 node_79
90 (Anterior basibranchial tooth plates)	1.000	1	node_73 0 ==> 1 node_72
91 (Extracleithrum)	1.000	1	Onychod 0 <=> 1 node_96

92 (Anocleithrum)	0.500	1	node_72 0 ==> 1 Macropoma
		1	node_83 0 ==> 1 Coccoderma
93 (Number of neural arches)	0.333	1	node_76 0 ==> 1 node_69
		1	node_82 0 ==> 1 node_81
		1	node_92 0 ==> 1 Hadronector
94 (Posterior neural and haemal spines)	0.500	1	node_96 0 --> 1 node_94
		1	node_56 1 --> 0 node_55
95 (Occipital neural arches)	0.333	1	node_87 0 ==> 1 node_86
		1	node_82 1 ==> 0 node_81
		1	node_93 0 ==> 1 node_92
96 (Ossified ribs)	1.000	1	node_78 0 ==> 1 node_67
97 (Ossified lung)	0.250	1	node_51 1 ==> 0 Holopterygius
		1	node_65 1 --> 0 node_64
		1	node_76 1 ==> 0 node_69
		1	node_70 1 ==> 0 Latimeria
98 (Basal plate of anterior dorsal fin)	0.333	1	node_53 1 --> 0 node_51
		1	node_90 1 ==> 0 node_87
		1	node_92 1 ==> 0 Hadronector
99 (Fin rays in the anterior dorsal fin)	0.200	1	node_52 0 ==> 1 Serenichthys
		1	node_86 0 ==> 1 node_85
		1	node_60 1 ==> 0 node_59
		1	node_69 1 ==> 0 node_68
		1	node_93 0 ==> 1 node_92
100 (Anterior dorsal fin)	0.500	1	node_82 0 ==> 1 node_79
		1	node_63 1 ==> 0 Chinlea
101 (Basal support of the second dorsal fin)	0.500	1	node_94 0 --> 1 node_93
		1	node_92 1 --> 0 Polyosteorhynchus
102 (Pelvics)	1.000	1	node_85 0 ==> 1 node_84
103 (Pelvic bones of each side)	1.000	1	node_85 0 --> 1 node_84
104 (Diphycercal tail)	1.000	1	node_96 1 ==> 0 node_95
105 (Caudal lobes)	0.167	1	node_53 0 ==> 1 node_51
		1	node_68 0 ==> 1 Rieppelia
		1	node_85 0 --> 1 node_84
		1	node_83 1 --> 0 Coccoderma
		1	node_88 0 ==> 1 Caridosuctor
		1	node_96 0 --> 1 node_95
106 (Fin rays)	0.333	1	node_94 0 ==> 1 node_93
		1	node_68 1 ==> 0 Rieppelia
		1	node_95 0 ==> 1 Gavinia
107 (Fin ray)	1.000	1	node_96 0 ==> 1 node_94
108 (Paired fin rays)	0.500	1	node_74 0 ==> 1 Holophagus
		1	node_84 0 ==> 1 node_83
109 (Median fin rays)	1.000	1	node_74 0 ==> 1 Holophagus
110 (Lateral line openings in scales)	0.333	1	Onychod 1 <=> 0 node_96
		1	node_86 0 ==> 1 node_85
		1	node_81 1 --> 0 node_80
111 (Ventral keel scales)	1.000	1	node_53 0 ==> 1 node_51
112 (Scale ornament)	0.333	1	node_67 0 ==> 1 node_66
		1	node_68 0 ==> 1 Foreyia
		1	node_73 0 --> 1 node_72

S2.6 Correspondence between the old and the new characters numbering

This study			From Forey (1998) to Toriño et al. (2021)	
#Character			#	Character
1	New character	<—>	-	
2	New character	<—>	-	
3	New definition	<—>	2	Snout bones
4	New character	<—>	-	
5	<——————>		5	Premaxilla
6	<——————>		6	Anterior opening of the rostral organ
7	New definition	<—>	3	Internasal
8	<——————>		7	Parietal
9	<——————>		8	Anterior and posterior pairs of parietals
10	<——————>		28	Parietals and postparietals
11	<——————>		11	Parietal descending process
12	New definition	<—>	9	Number of supraorbitals/tectals
13	<——————>		10	Preorbital
14	<——————>		12	Intertemporal
15	<——————>		13	Postparietal descending process
16	<——————>		14	Supratemporal descending process
17	<——————>		18	Posterior margin of the skull roof
18	<——————>		15	Extrascapulars
19	<——————>		16	Extrascapulars
20	New definition	<—>	17	Number of extrascapulars
21	New character	<—>	-	
22	<——————>		19	Supraorbital sensory canal
23	New definition	<—>	23	Supraorbital sensory canals opening through bones
24	<——————>		20	Medial branch of otic canal
25	<——————>		22	Anterior branches of supratemporal commissure
26	<——————>		26	Pit lines
27	<——————>		25	Middle and posterior pit lines
28	New definition	<—>	27	Parietals and postparietals
29	<——————>		29	Cheek bones
30	<——————>		30	Spiracular (postspiracular)
31	<——————>		40	Postorbital
32	<——————>		41	Postorbital
33	<——————>		42	Postorbital
34	<——————>		43	Postorbital
35	New character	<—>	-	
36	<——————>		37	Squamosal
37	<——————>		34	Squamosal
38	<——————>		38	Preoperculum
39	<——————>		39	Preoperculum
40	New character	<—>	-	
41	<——————>		32	Suboperculum
42	New definition	<—>	35+36	Lachrymojugal
43	New character	<—>	-	
44	<——————>		51	contact between the lachrymojugal and the preorbital or tectal-supraorbital series
45	New character	<—>	-	
46	New character	<—>	-	
47	New character	<—>	-	
48	New definition	<—>	44	Infraorbital canal within the postorbital

This study		From Forey (1998) to Toriño et al. (2021)	
#	Character	#	Character
49	<————————>	45	Infraorbital sensory canal
50	New definition <——>	46	Jugal sensory canal
51	<————————>	47	Jugal canal
52	<————————>	50	Infraorbital, jugal and preopercular sensory canals
53	<————————>	48	Pit lines
54	New definition <——>	49	Ornaments upon cheek bones
55	New character <——>	-	
56	<————————>	52	Sclerotic ossicles
57	<————————>	53	Retroarticular and articular
58	<————————>	57	Dentary
59	<————————>	65	Dentary
60	New definition <——>	54	Dentary teeth
61	<————————>	66	Principal coronoid
62	New definition <——>	55	Number of anterior coronoids
63	<————————>	56	Coronoid
64	<————————>	67	Coronoid fangs
65	New definition <——>	68	Prearticular and/or coronoid teeth
66	<————————>	60	Subopercular branch of the mandibular sensory canal
67	<————————>	61	Dentary sensory pore
68	New character <——>	-	
69	New character <——>	-	
70	<————————>	58	Oral pit line
71	<————————>	69	Orbitosphenoid and basisphenoid regions
72	<————————>	71	Processus connectens
73	<————————>	72	Basipterygoid process
74	<————————>	74	Temporal excavation
75	<————————>	75	Otico-occipital
76	<————————>	76	Supraoccipital
77	New character <——>	-	
78	<————————>	78	Parasphenoid
79	<————————>	79	Suprapterygoid process
80	<————————>	80	Vomers
81	<————————>	81	Vomers
82	<————————>	82	Prootic
83	<————————>	83	Superficial ophthalmic branch of anterodorsal lateral line nerve
84	<————————>	84	Process on braincase for articulation of infrabranchial 1
85	<————————>	85	Separate lateral ethmoids
86	<————————>	86	Separate basioccipital
87	<————————>	87	Dorsum sellae
88	<————————>	110	Ventral swelling of the palatoquadrate
89	New character <——>	-	
90	New character <——>	-	
91	<————————>	88	Extracleithrum
92	<————————>	89	Anocleithrum
93	New character <——>	-	
94	<————————>	90	Posterior neural and haemal spines
95	<————————>	91	Occipital neural arches
96	<————————>	92	Ossified ribs
97	New definition <——>	107	Ossified lung

This study		From Forey (1998) to Toriño et al. (2021)	
#Character		#	Character
98	<————————>	101	Basal plate of D1
99	<————————>	96	Fin rays in D1
100	<————————>	98	D1
101	<————————>	102	D2 basal support
102	<————————>	100	Pelvics
103	<————————>	108	Pelvic bones of each side
104	<————————>	93	Diphycercal tail
105	<————————>	97	Caudal lobes
106	<————————>	94	Fin rays
107	<————————>	95	Fin ray
108	<————————>	99	Paired fin rays
109	<————————>	103	Median fin rays
110	<————————>	105	Lateral line openings in scales
111	<————————>	109	Ventral keel scales
112	<————————>	104	Scale ornament

This study			From Forey (1998) to Toriño et al. (2021)	
	#Character		#	Character
-	Deleted	<——	1	Intracranial joint margin
-	Deleted	<——	4	Premaxillae
-	Deleted	<——	21	Otic canal
-	Deleted	<——	24	Anterior pit line
-	Deleted	<——	31	Preoperculum
-	Deleted	<——	33	Quadratojugal
-	Deleted	<——	62	Ornaments
-	Deleted	<——	63	Dentary
-	Deleted	<——	64	Splenial
-	Deleted	<——	70	Optic foramen
-	Deleted	<——	73	Antotic process
-	Deleted	<——	77	Vestibular fontanelle
-	Deleted	<——	107	Scales
