# Supplementary material for: A deep dive into the coelacanth phylogeny
Source: PLoS One. 2025 Jun 6;20(6):e0320214. doi: 10.1371/journal.pone.0320214 (PMC12143573; doi:10.1371/journal.pone.0320214)
Supplement: S4 Data — (PDF) [file pone.0320214.s004.pdf]

Supporting information 4 for:

A deep dive within the coelacanth phylogeny

Christophe Ferrante[1, 2¶], Lionel Cavin[1, 2¶*]

[1]Natural history Museum of Geneva, Geneva, Switzerland.

[2]Department of Earth Sciences, University of Geneva, Geneva, Switzerland.

*Corresponding authors

E-mail: lionel.cavin@ville-ge.ch (LC)

E-mail: paleo-ferrante.ch@bluewin.ch (CF)

¶These authors contributed equally to this work.

Table of contents

PLATE 1 — DERMAL BONES OF THE SKULL ROOF OF SOME COELACANTHS 2

PLATE 2 — DERMAL BONES OF THE SKULL ROOF OF SOME COELACANTHS 3

PLATE 3 — DERMAL BONES OF THE SKULL ROOF OF SOME COELACANTHS 4

PLATE 4 — DERMAL BONES OF THE SKULL ROOF OF SOME COELACANTHS 5

PLATE 5 — DERMAL BONES OF THE SKULL ROOF OF SOME COELACANTHS 6

PLATE 6 — DERMAL BONES OF THE SKULL ROOF OF SOME OSTEICHTHYES 7

PLATE 7 — DERMAL BONES OF THE CHEEK, LOWER JAW AND PECTORAL GIRDLE OF SOME COELACANTHS 8

PLATE 8 — DERMAL BONES OF THE CHEEK, LOWER JAW AND PECTORAL GIRDLE OF SOME COELACANTHS 9

PLATE 9 — DERMAL BONES OF THE CHEEK, LOWER JAW AND PECTORAL GIRDLE OF SOME OSTEICHTHYES 10

PLATE 10 — GULAR PLATES OF SOME COELACANTHS 11

PLATE 11 — GULAR PLATES OF SOME COELACANTHS 12

PLATE 12 — GULAR PLATES OF SOME COELACANTHS 13

PLATE 13 — PALATOQUADRATE OF SOME COELACANTHS 14

PLATE 14 — PALATOQUADRATE OF SOME COELACANTHS 15

REFERENCE OF THE ILLUSTRATIONS 16

Plate 1 — Dermal bones of the skull roof of some coelacanths

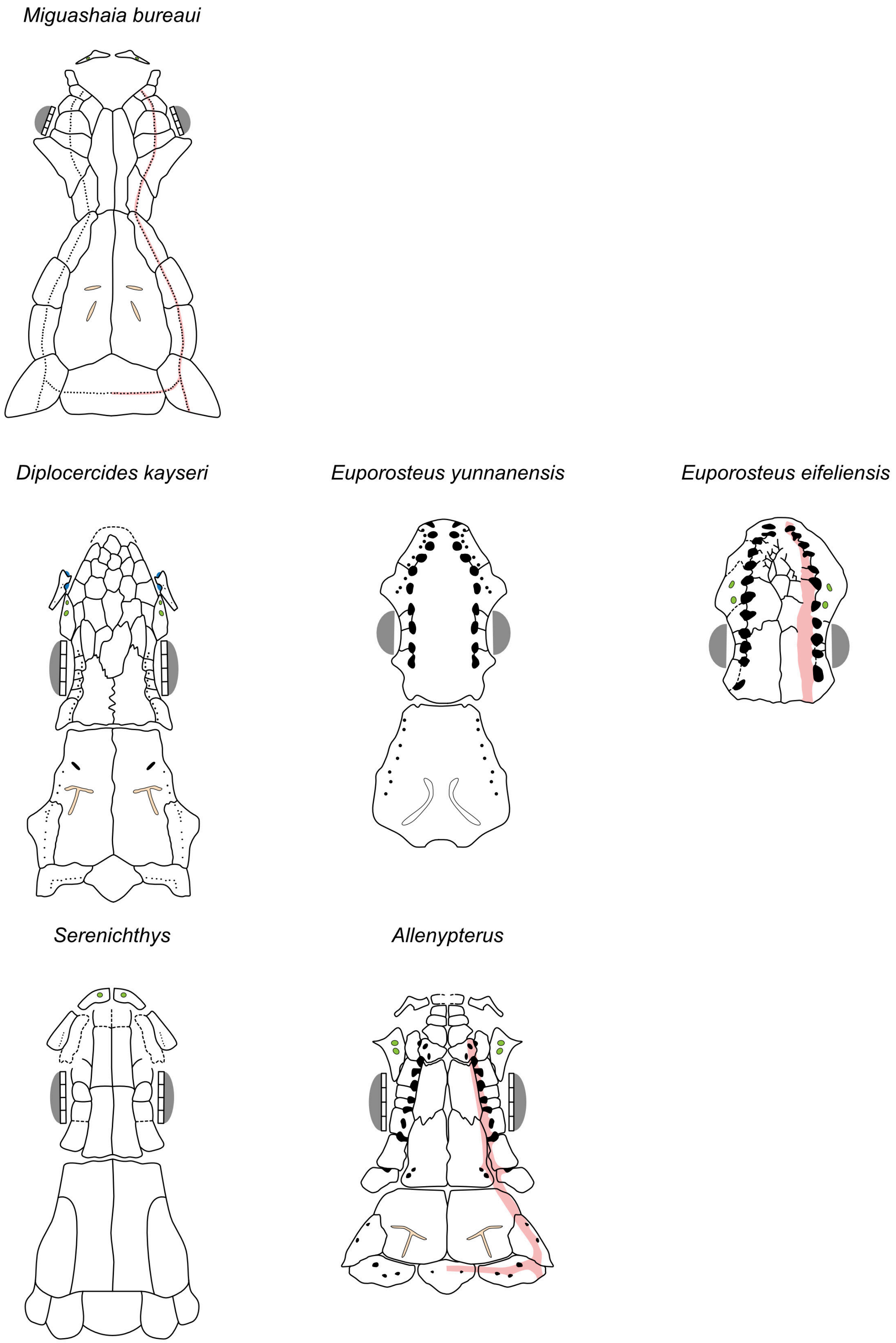

Plate 2 — Dermal bones of the skull roof of some coelacanths

Hadronector

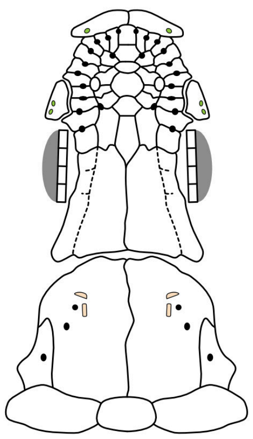

Sassenia groenlandica

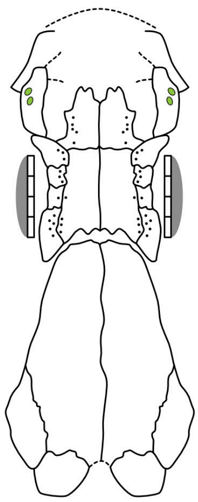

Spermatodus

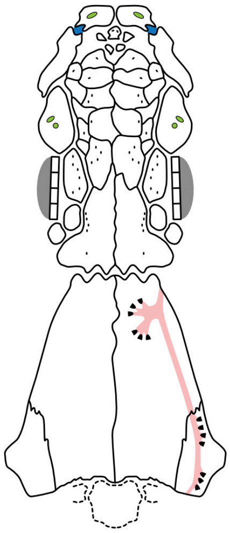

Caridosuctor

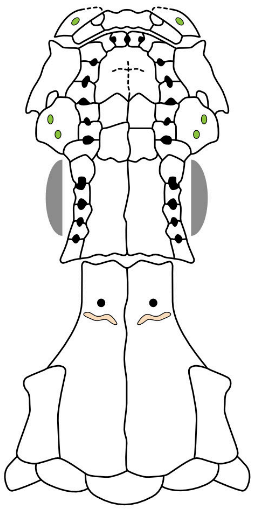

Rhabdoderma elegans

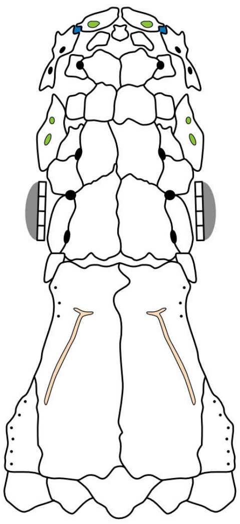

Plate 3 — Dermal bones of the skull roof of some coelacanths

Coelacanthus granulatus

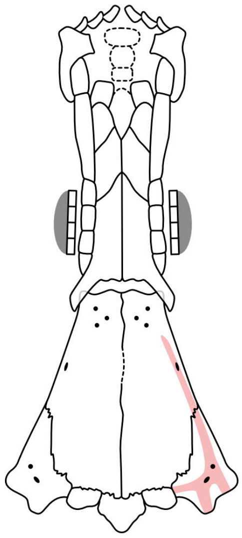

Laugia groenlandica

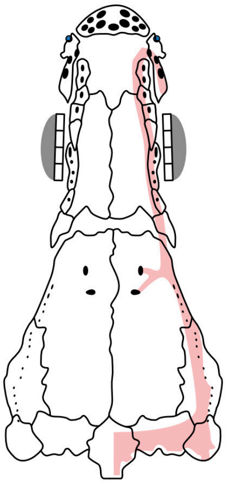

Coccoderma

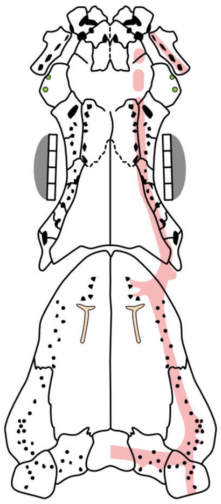

Yunnacoelacanthus

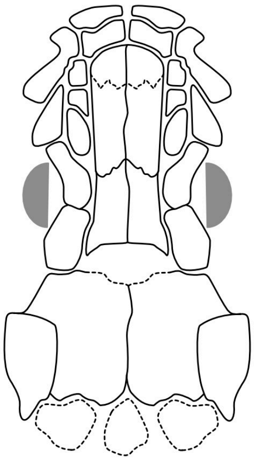

Whiteia woodwardi

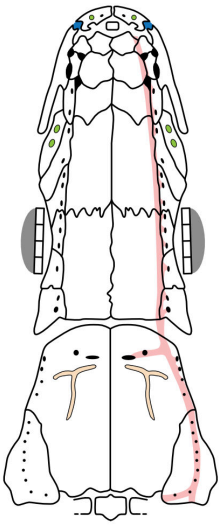

Guizhoucoelacanthus

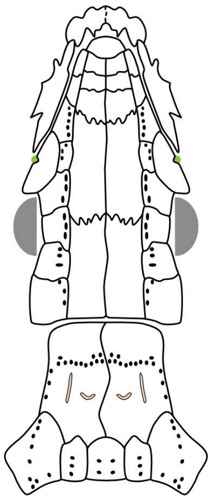

Wimania

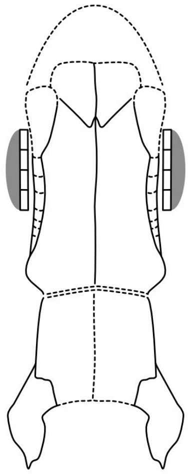

Axelia

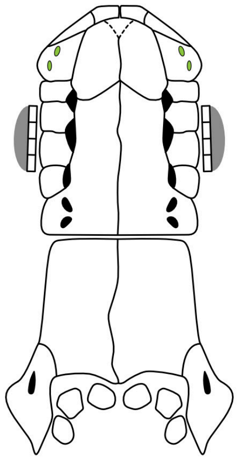

Atacamaia

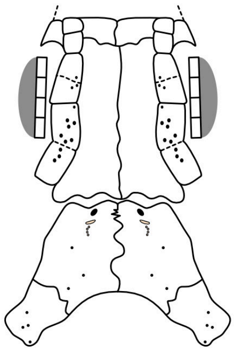

Plate 4 — Dermal bones of the skull roof of some coelacanths

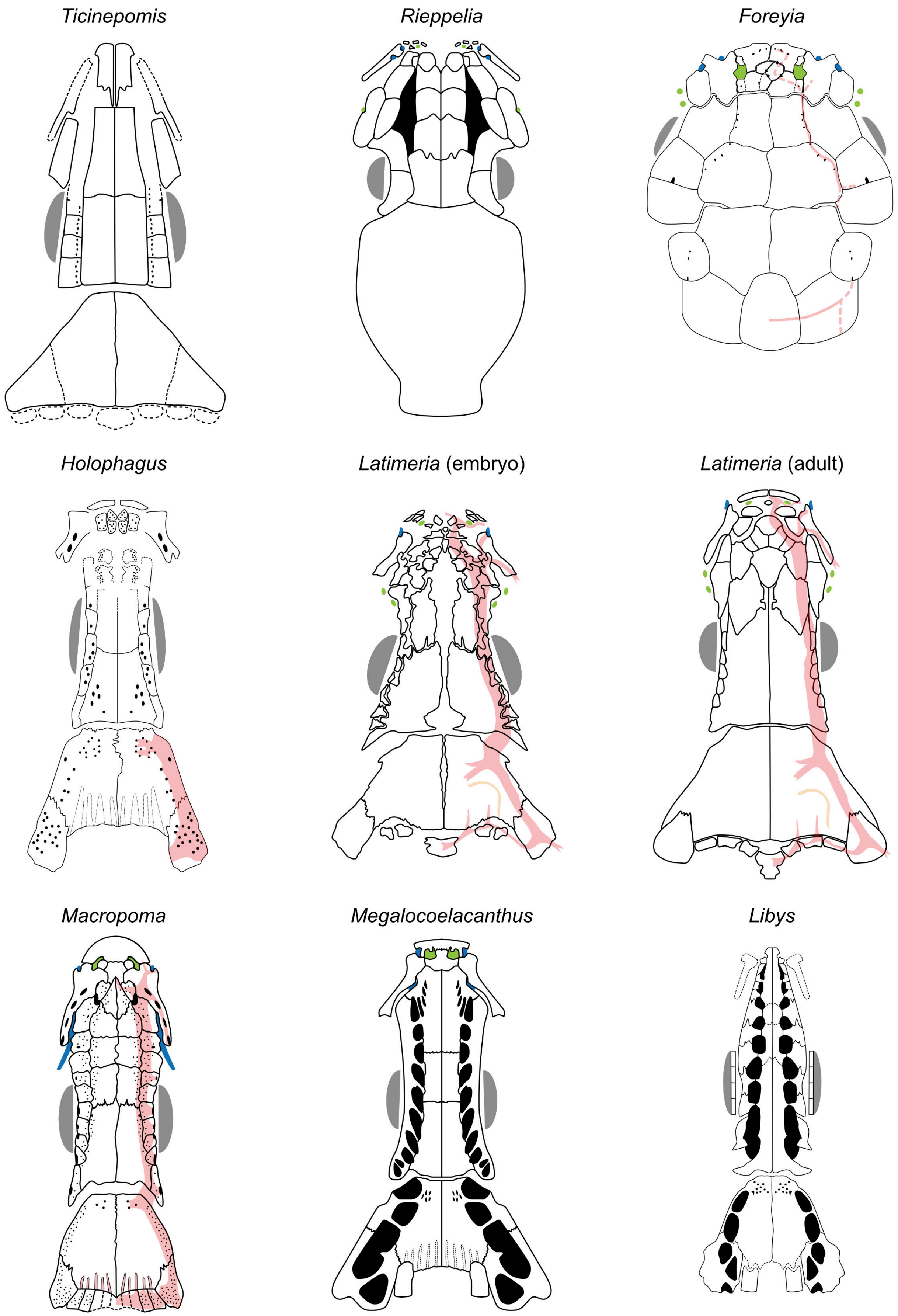

Plate 5 — Dermal bones of the skull roof of some coelacanths

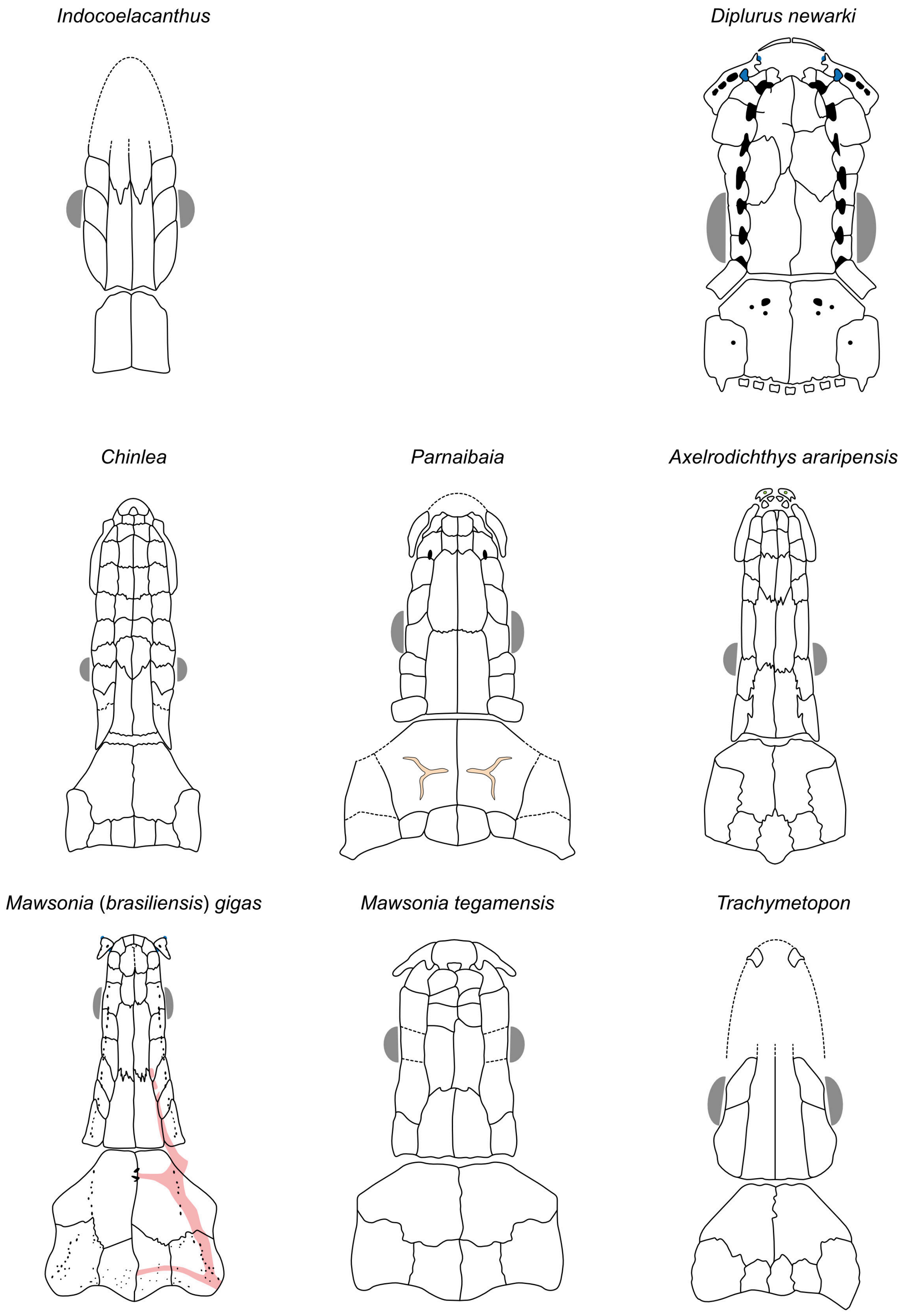

Plate 6 — Dermal bones of the skull roof of some Osteichthyes

Onychodus jandemarrai

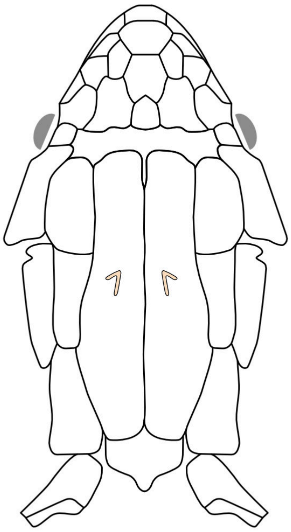

Porolepis brevis

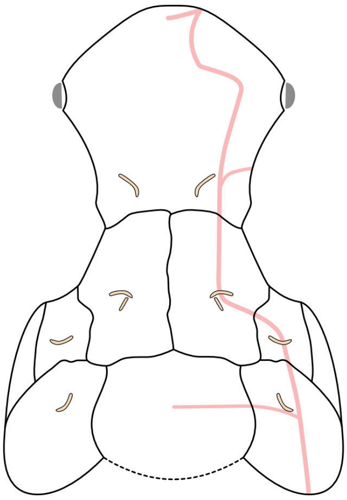

Mimipiscis bartrami

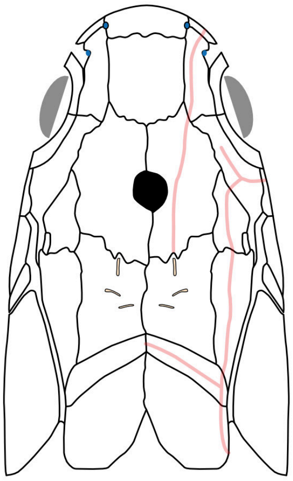

Guiyu oneiros

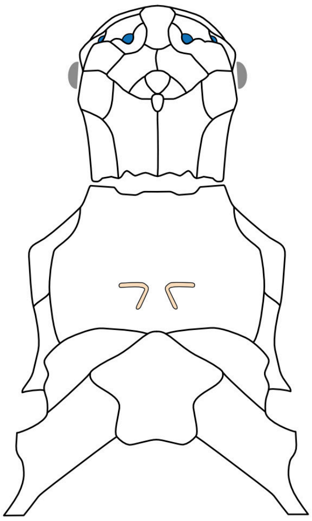

Plate 7 — Dermal bones of the cheek, lower jaw and pectoral girdle of some coelacanths

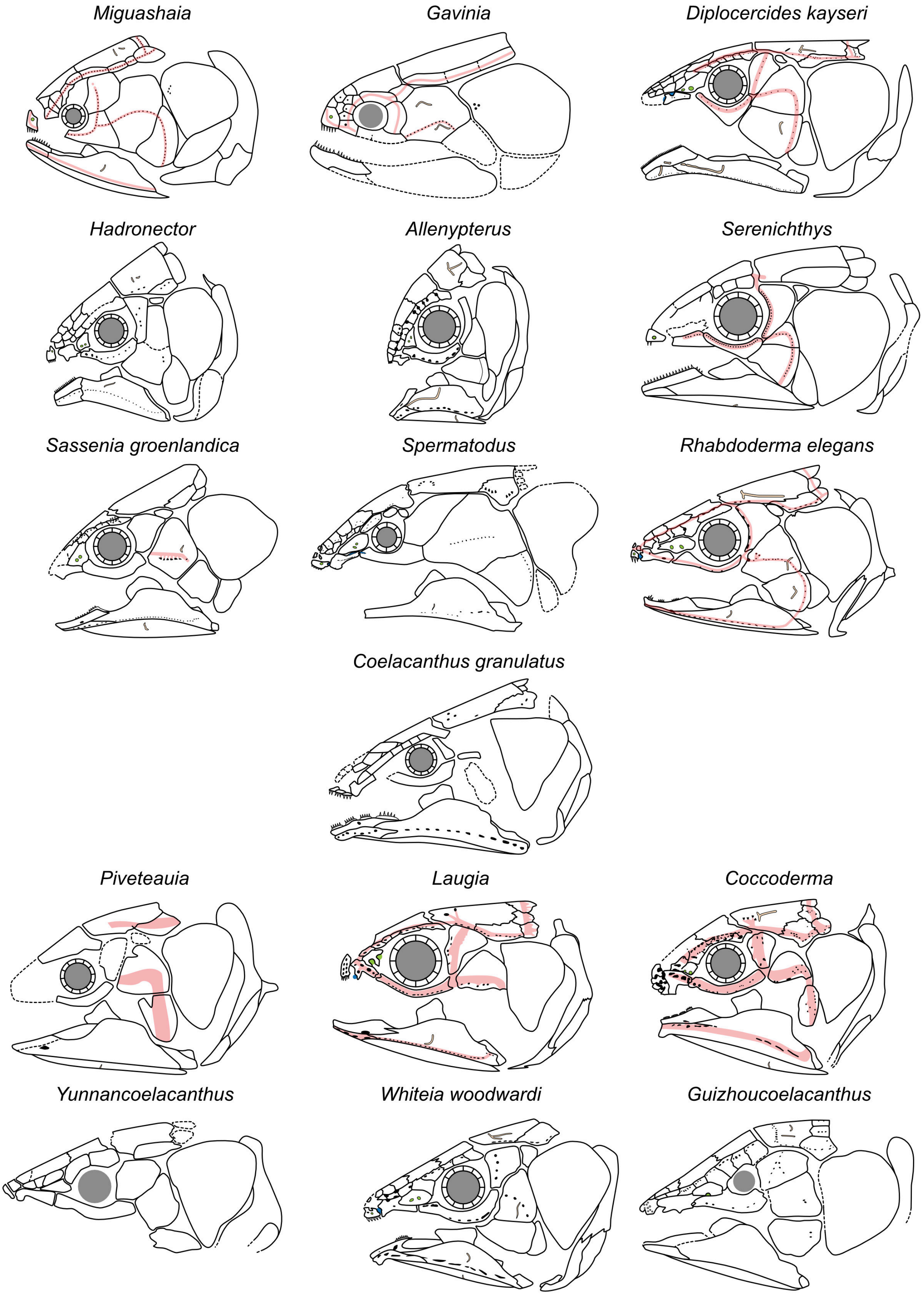

Plate 8 — Dermal bones of the cheek, lower jaw and pectoral girdle of some coelacanths

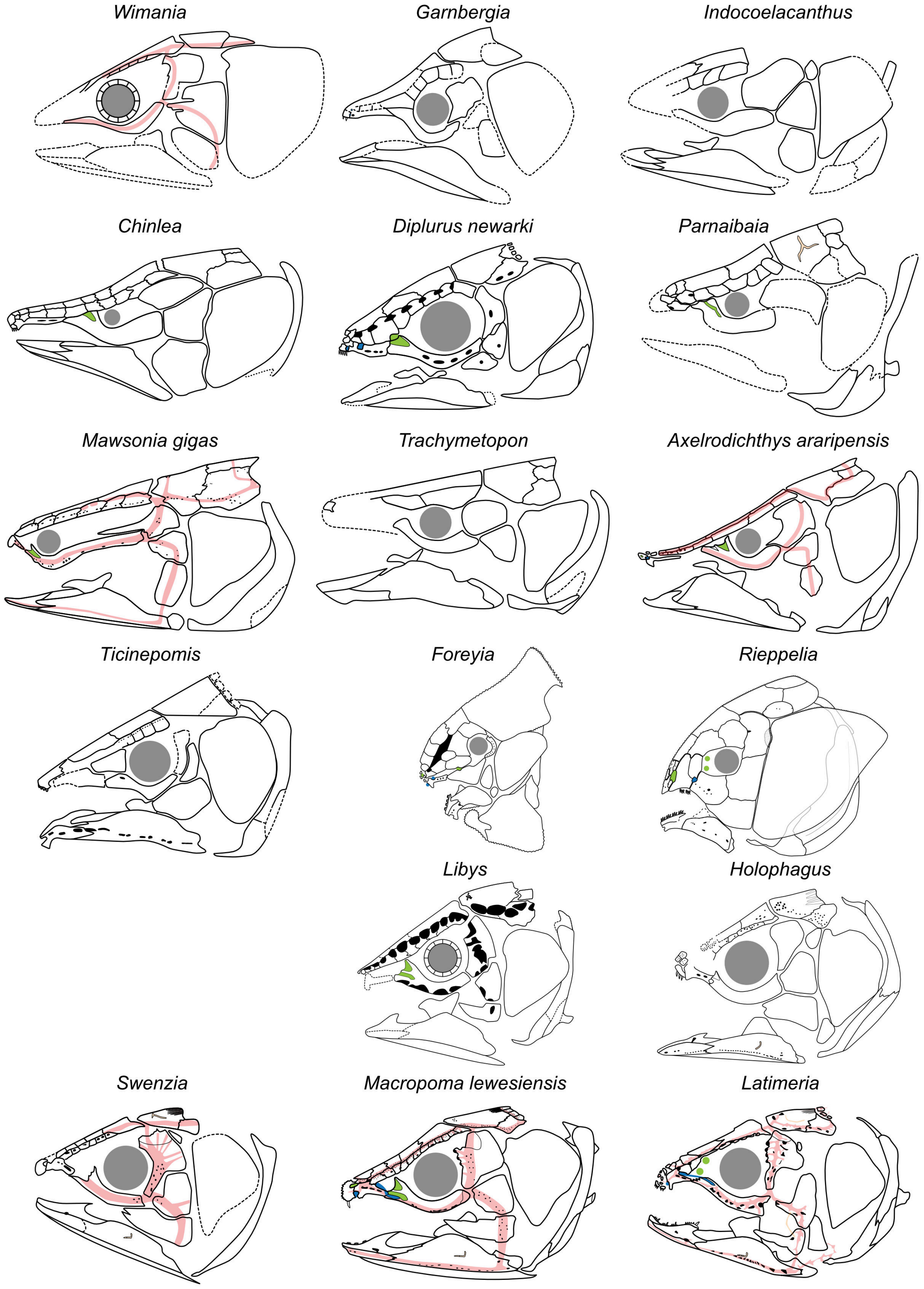

Plate 9 — Dermal bones of the cheek, lower jaw and pectoral girdle of some Osteichthyes

Onychodus jandemarrai

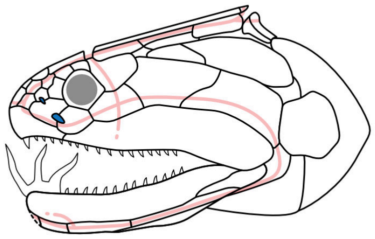

Porolepis brevis

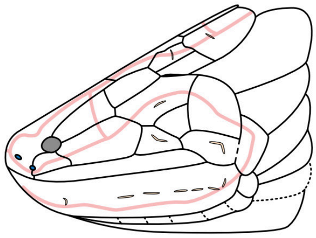

Mimipiscis bartrami

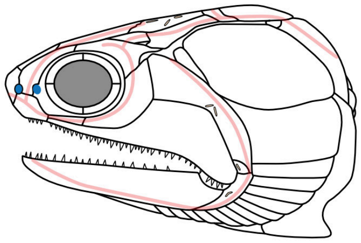

Guiyu oneiros

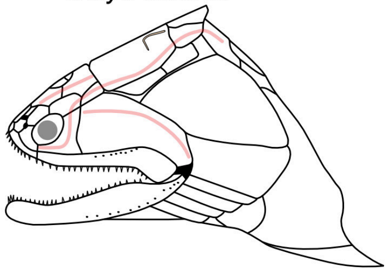

Plate 10 — Gular plates of some coelacanths

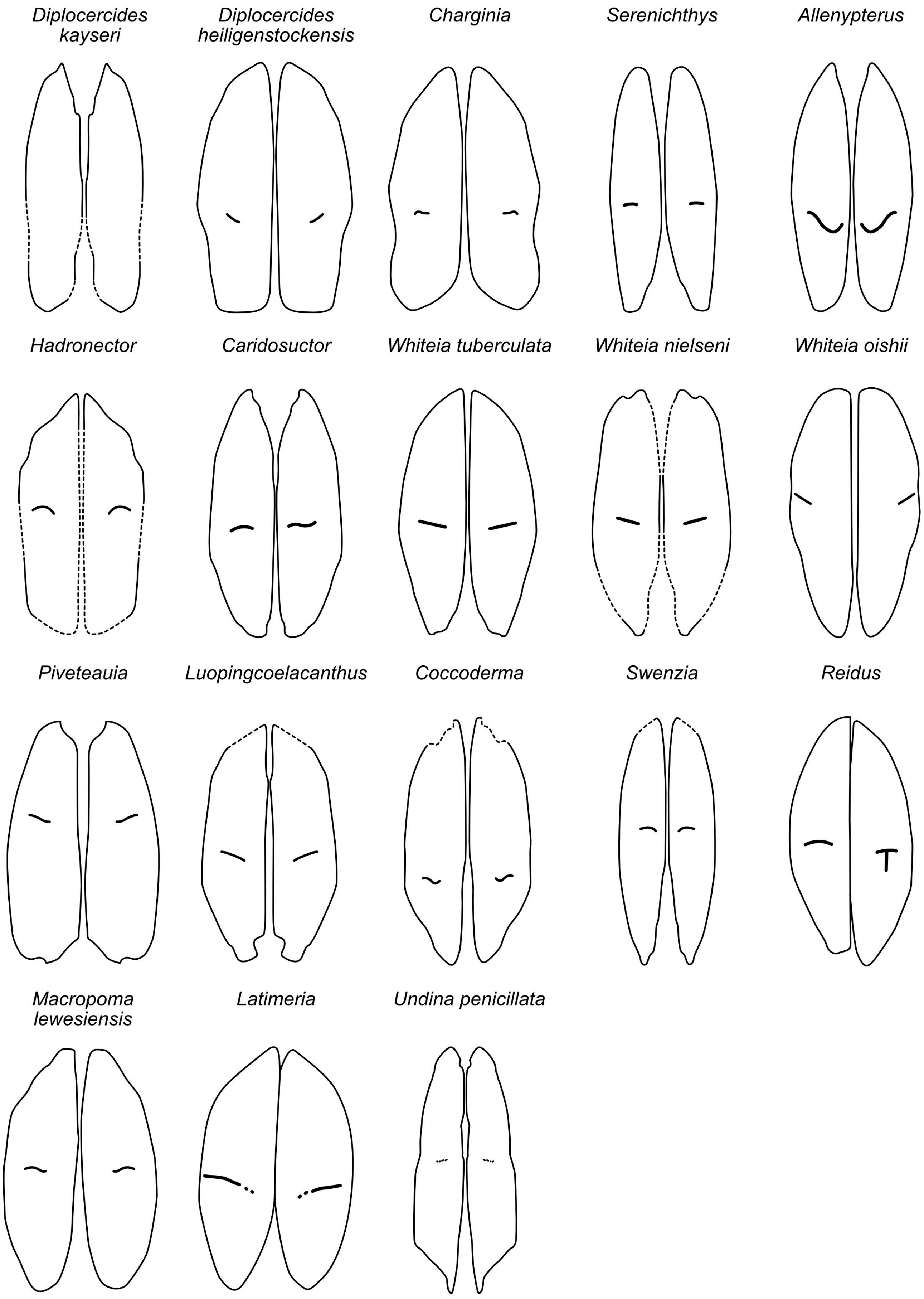

Plate 11 — Gular plates of some coelacanths

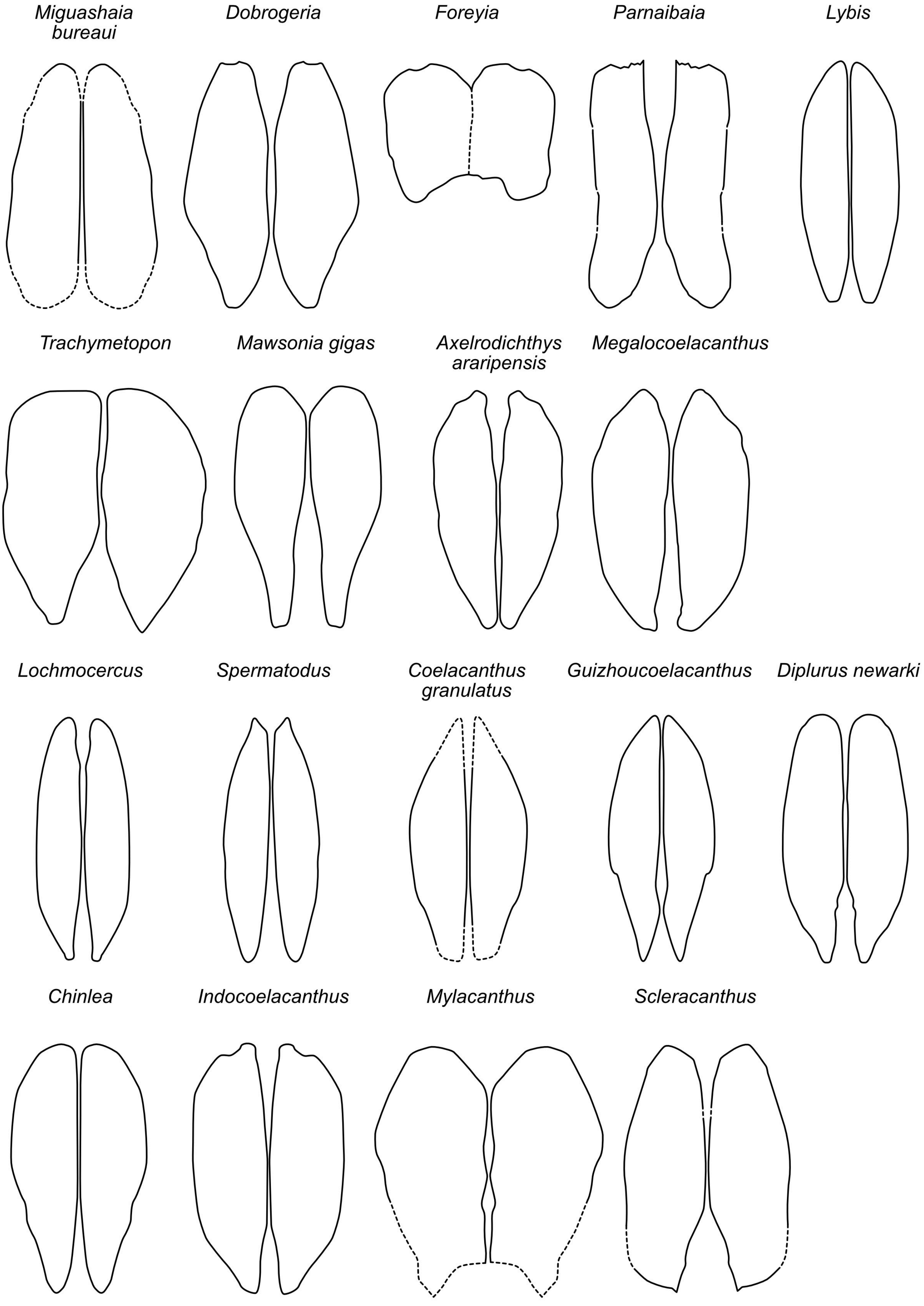

Plate 12 — Gular plates of some coelacanths

Allenypterus

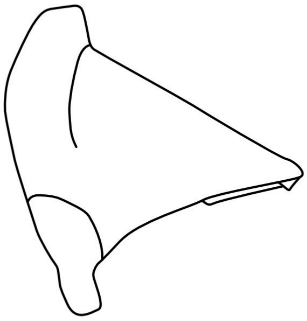

Coelacanthus granulatus

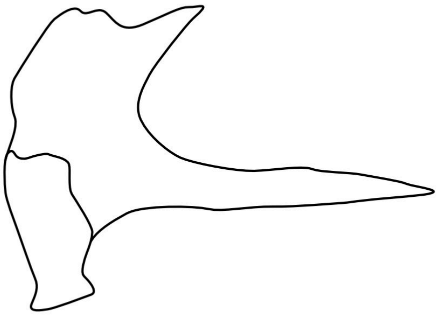

Diplocercides kayseri

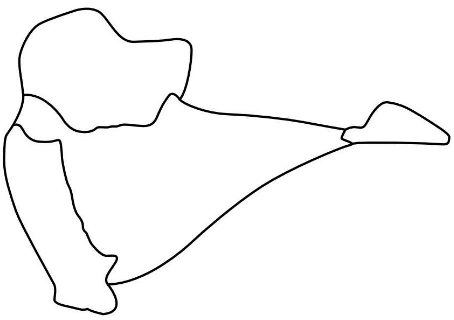

Diplurus newarki

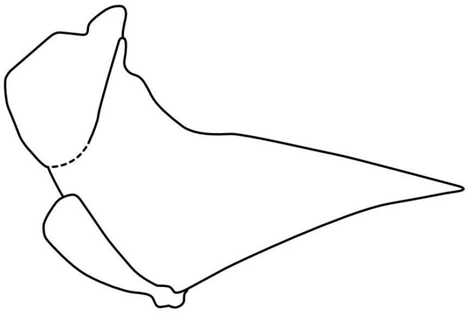

Axelrodichthys araripensis

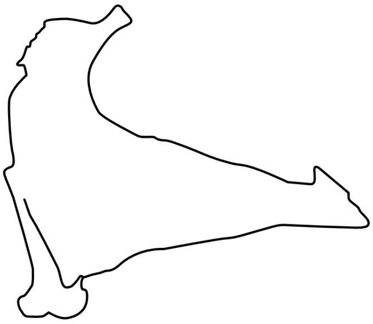

Axelrodichthys megadromos

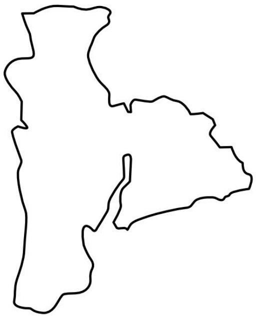

Hadronector

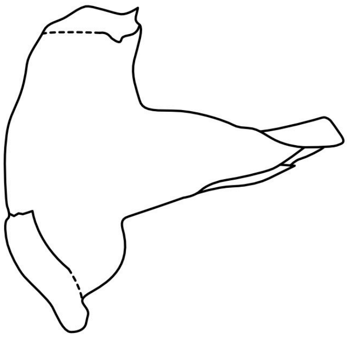

Lochmocercus

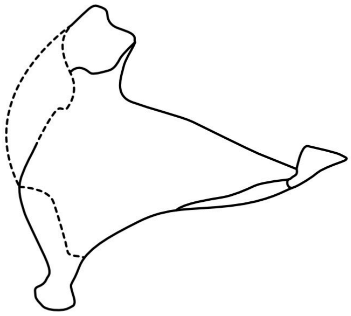

Lualabaea

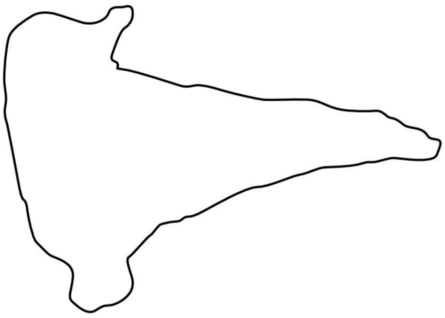

Mawsonia gigas

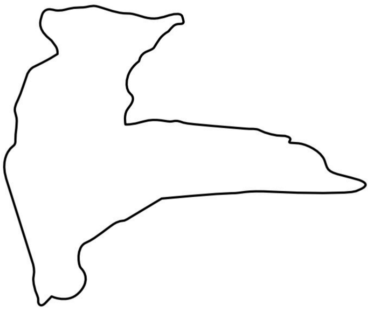

Mawsonia gigas

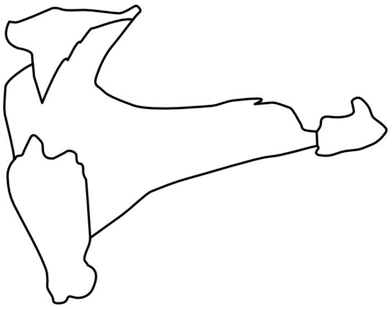

Axelrodichthys lavocati

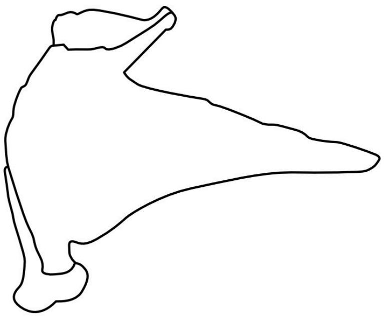

Plate 13 — Palatoquadrate of some coelacanths

Polyosteorynchus

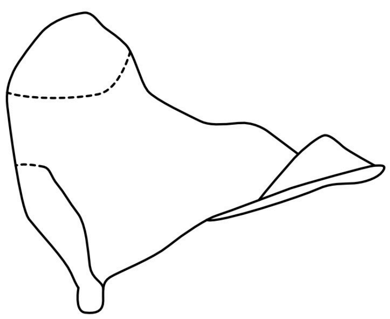

Rhabdoderma elegans

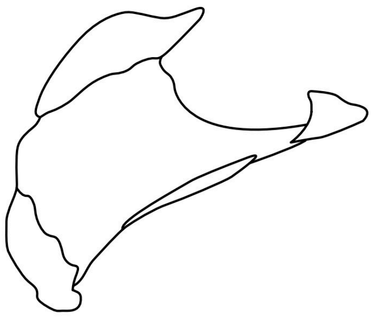

Sassenia tuberculata

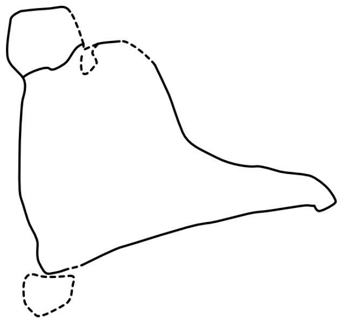

Trachymetopon

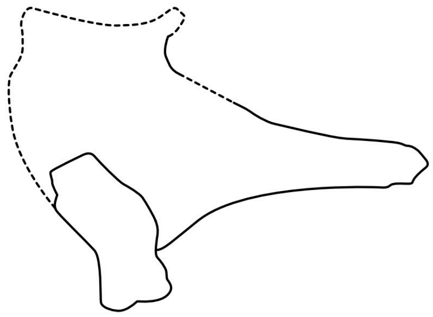

Indocoelacanthus

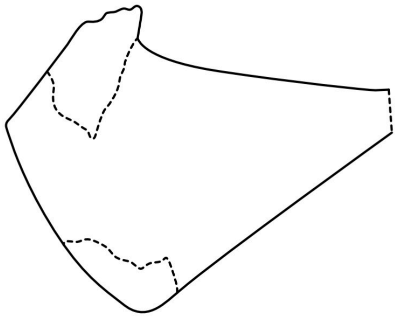

Whiteia woodwardi

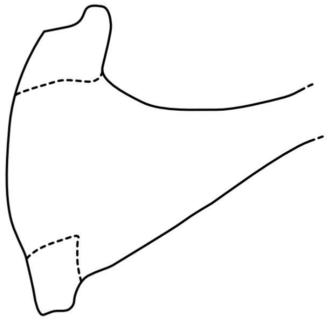

Wimania

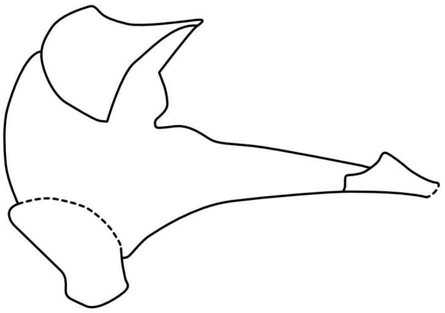

Axelia

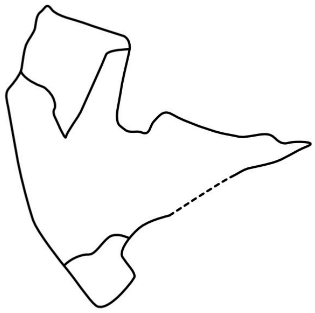

Youngichtys

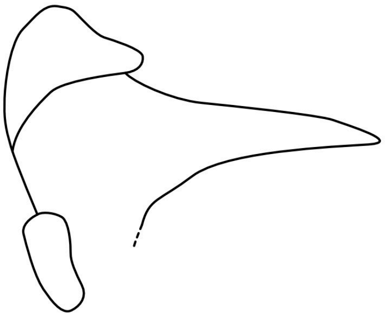

Plate 14 — Palatoquadrate of some coelacanths

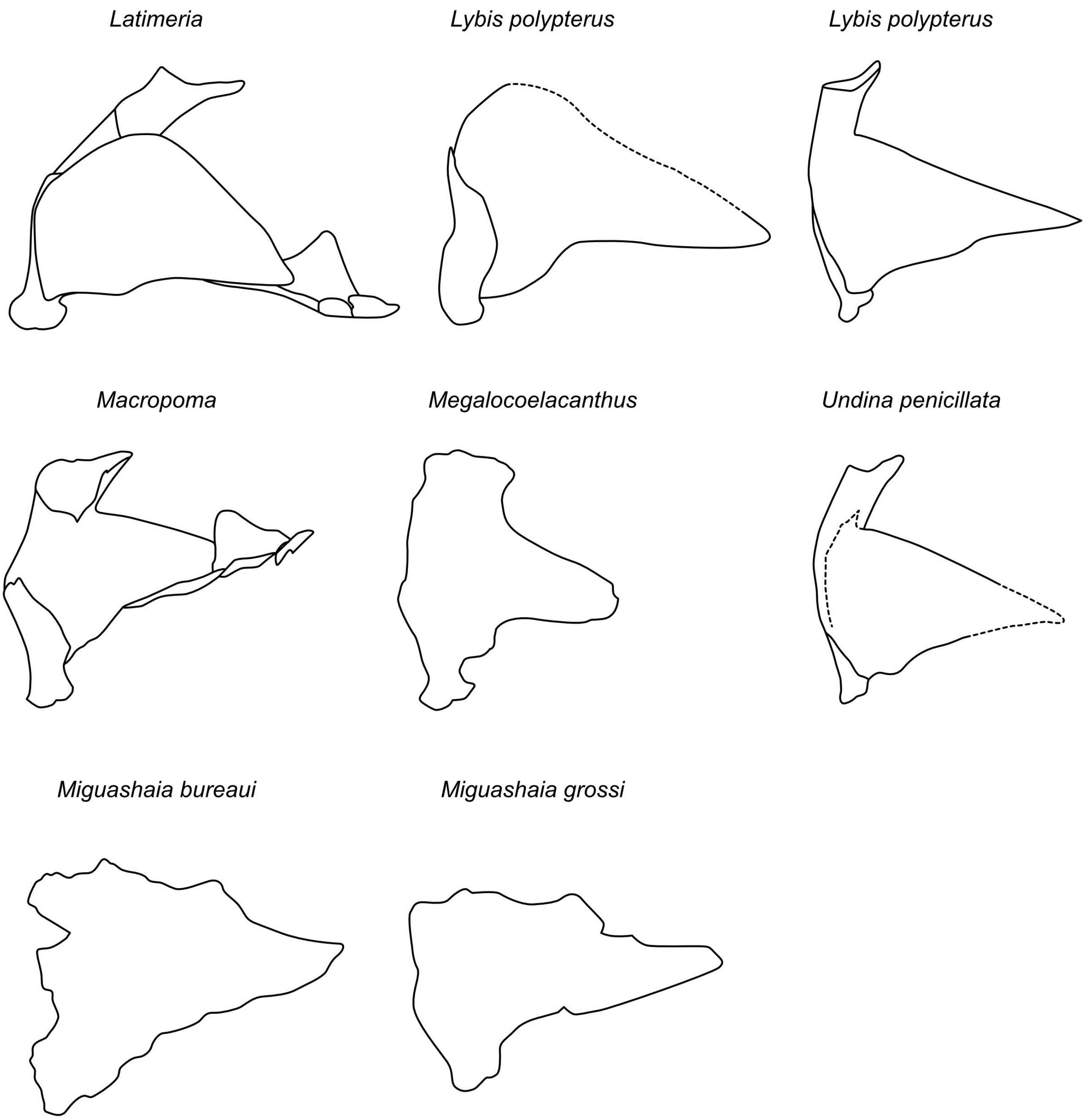

Reference of the illustrations

The illustrations on the plates above and many figures in the main article have been redrawn, modified or adapted from various authors listed below.

Coelacanths

Allenypterus montanus: Forey (1998, figs 3.5, 4.6).

Atacamaia solitaria: Arratia and Schultze (2015, figs 3, 4).

Axelia robusta: Stensiö (1921, fig. 43, pl. 11).

Axelrodichthys araripensis: Maisey (1986, figs 14, 26a); Forey (1998, figs 4.17, 5.10); Fragoso et al. (2018, figs 2b, 15a, 19a, S1c, S13).

Axelrodichthys megadromos: Cavin et al. (2016, fig. 2A1).

Axelrodichthys lavocati: Fragoso et al. (2018, fig.19C).

Caridosuctor populosum: Forey (1998, fig. 3.3c); Lund & Lund (1985, figs 18, 24).

Chagrinia enodis: Cloutier (1996, fig. 9D).

Chinlea sorenseni: Elliott (1987, figs 2a-b, 3).

Coccoderma suevicum: Forey (1998, figs 3.11b, 4.11, 4.12B, 5.7); BSM 1870.XIV.23 (personal observation).

Coelacanthus granulatus: Schaumberg (2005, figs 5, 6, 10, 11, 18); Forey (1998, figs 3.7, 5.4a, 11.4).

Diplocercides kayseri: Stensiö (1937, figs 3, 7a, 17, pl. 10 fig 1); Forey (1998, figs 3.4, 4.5, 5.2, 7.1D); Cloutier (1996, fig. 9B).

Diplocercides heiligenstockensis: Cloutier (1996, fig. 9C).

Diplurus newarki: Schaeffer (1952, figs 4, 6, 7a, 10a, pl. 16.2).

Dobrogeria aegyssensis: Cavin & Gradinăru (2014, fig. 12B).

Euporosteus eifelensis: Stensiö (1937, fig. 10), Forey (1998, fig. 6.3a).

Euporosteus yunnanensis: Zhu et al. (2012, fig. 3b,e).

Foreyia maxkuhni: Cavin et al. (2017, fig. S6d); PIMUZ A/I 4620 (personal observation).

Garnbergia ommata: Martin & Wenz (1984, fig. 1, pl. 1, fig 1 pl. 2 fig. 1).

Gavinia syntrips: Long (1999, figs 5b, 6b, 7, 12); Mondéjar-Fernàndez (2020, fig. 17C).

Guizhoucoelacanthus guanlingensis: Geng et al. (2009, fig. 2).

Hadronector donbairdi: Lund & Lund (1985, figs 38, 43, 45); Cloutier (1991, fig. 4); Forey (1998, fig. 4.7).

Holophagus gulo: Forey (1998, figs 3.18, 5.12b, 11.8).

Indocoelacanthus robustus: Jain (1974, figs 2, 3, 4, 5).

Latimeria chalumnae: Forey (1998, figs 2.3, 2.5, 3.1, 3.2, 4.1, 5.1, 7.1B); Jarvik (1980, fig. 222).

Laugia groenlandica: Forey (1998, figs 3.8a, 3.9a, 4.10).

Libys polypterus: Forey (1998, fig. 3.17); Reis (1888, pl. 3 fig 1); Hauser & Martill (2013, fig. 5b); Mäuser (2018, fig 3.2); BSM 1870.XIV.502 and BSM AS.I.801 (personal observation).

Lochmocercus aciculodontus: Lund & Lund (1985, figs 68, 70).

Lualabaea lerichei: MRAC R.G.10.046 (personal observation).

Luopingcoelacanthus eurylacrimalis: Wen et al. (2012, fig. 1).

Macropoma lewesiensis: Forey (1998, figs 3.19a, 3.21a, 4.18, 4.19, 7.2A).

Mawsonia gigas: Maisey (1986, figs 1, 7a, 10); Fragoso et al. (2018, fig.19B); Yabumoto (2002, figs 2, 4); Toriño et al. (2021, figs 3, 4).

Megalocoelacanthus dobiei: Dutel et al. (2012, figs 2, 4, 5, 11A, 14C).

Miguashaia bureaui: Cloutier (1996, figs 3, 5, 11A); Forey (1998, figs 3.3A, 4.4); Mondéjar-Fernández (2020, fig. 17D).

Miguashaia grossi: Forey et al. (2000, fig. 4A).

Mylacanthus spinosus: Stensiö (1921, pl.19 fig. 3).

Parnaibaia maranhaoensis: Yabumoto (2008, fig. 3).

Piveteauia madagascariensis: Clément (1999, figs 1, 2).

Polyosteorhynchus simplex: Lund & Lund (1985, fig. 54).

Reidus hilli: Graf (2012, fig. 2A,D).

Rhabdoderma elegans: Forey (1981, figs 2, 5, 6, 7); Forey (1998, figs 4.8, 7.1E, 11.14).

Rieppelia heinzfurreri: Ferrante & Cavin (2023, figs 2c, S8).

Sassenia groenlandica: Forey (1998, figs 3.11, 4.13).

Sassenia tuberculata: Stensiö (1921, pl.10 fig. 1, 4).

Scleracanthus asper: Stensiö (1921, pl.19 fig. 1).

Serenichthys kowiensis: Gess & Coates (2015, figs 1c, 2).

Spermatodus pustulosus: Westoll (1939, figs 1a, 2); Forey 1998, figs 3.12, 3.13a, 3.14, 5.8a).

Swenzia latimerae: Clément (2005, figs 5, 9b).

Ticinepomis peyeri: PIMUZ-T3925, PIMUZ-T2651 and PIMUZ-T978 (personal observation).

Trachymetopon liassicum: Dutel et al. (2015, figs 1b, 3, 4, 5).

Undina penicillata: BSM 1873 XIV 517 (personal observation).

Whiteia woodwardi: Forey (1998, figs 3.15, 4.15, 5.9a); Moy-Thomas (1935 (fig. 4).

Whiteia tuberculata: Forey (1998, 5.9F).

Whiteia nielseni: Forey (1998, 5.9D).

Whiteia oishii: Yabumoto & Brito (2016, fig. 3B).

Wimania sinuosa: Stensiö (1921, figs 21, 25, 26, pl. 4.1).

Youngichthys xinhuainsis: Wang & Liu (1981, fig. 3).

Yunnancoelacanthus acrotuberculatus: Wen et al. (2013, fig. 7b-c).

<u>Osteichthyes</u>

Guiyu oneiros: Mondéjar-Fernández (2019, fig.16A); Zhu et al. (2009, fig.3).

Mimipiscis bartrami: Choo (2012, fig. 18a-b).

Onychodus jandermarrai: Andrews et al. (2006, fig. 4); Mondéjar-Fernández (2019, fig.16B).

Porolepis brevis: Jarvik (1980, figs 184B, 186A); Mondéjar-Fernández et al. (2019, fig.10B).
