# Supplementary material for: A deep dive into the coelacanth phylogeny
Source: PLoS One. 2025 Jun 6;20(6):e0320214. doi: 10.1371/journal.pone.0320214 (PMC12143573; doi:10.1371/journal.pone.0320214)
Supplement: Attachment — Submitted filename: Reply to reviewers comments.docx [file pone.0320214.s006.docx]

# Answers to reviewer

Dear reviewer,

We would like to thank you for taking the time to review our long manuscript and for providing us with your valuable comments that improve it.

We have taken all of your comments into account. We have made all of the corrections you requested and have followed up on almost all of your comments, except one (see below for details).

Best regards,

The authors.

**Reviewer:**

1) There are some omissions in the comprehensive review of extinct coelacanth phylogenetic diversity and phylogenetic studies that need to be addressed. A recently published revision of *Diplurus newarki* from 2022 (<https://bmcecolevol.biomedcentral.com/articles/10.1186/s12862-022-02043-4>) was not cited or discussed, and the species erected in that contribution ("*D. enigmaticus*") and in a second study by one of the authors ("*Whiteia giganteus*" <https://palaeo-electronica.org/content/2023/3804-big-dockum-group-coelacanth>) were not included in your comprehensive review without comment. These studies provide phylogenies that place these species. Even if you disagree with the erection of these new taxa (and indeed, the authors of those studies split them based on few characters), the goal of your manuscript is clearly to provide a much-needed service by reviewing extinct coelacanth diversity in the context of their phylogeny. So, you need note why you do not consider these species valid and for what reasons if you exclude them from your list (especially since they are recently published). Secondly, the first study indicates that some features considered in your paper to be intraspecific variation (e.g., ridge counts on the opercle) are suggested to correspond to different species by the first paper. Also note that the first paper documented teeth from *Diplurus newarki*, which is important insofar as you note that this character coding was one you needed to review. This excellent paper is a service to the literature and these corrections will only further that service.

# Answers to comment No°1

In this paper, we selected coelacanth species for which the available descriptions and diagnoses are relevant to characterize the coding of the genera used in our analyses. Since our phylogenetic analysis was based on genera and not on species (contrary to Clement et al., 2024), our aim was not to explain and assert the validity of all known species.

In the first version of our manuscript, we did not cite Brownstein (2023) and Brownstein & Bissell (2022) that both describe new species of coelacanths, namely *Whiteia* *giganteus* (Brownstein, 2023) and *Diplurus* *enigmaticus* (Brownstein & Bissell, 2022). Nevertheless, we agree that we need to cite these two articles with some brief comments on the species that have been erected. Please find below, the paragraphs we added:

**New text.** Lines 3494-3510 - (paragraph 5. Corrected and commented taxon scoring - *Whiteia*)

“Recently, Brownstein (2023) erected the species *Whiteia giganteus* based on a large, nearly complete but heavily weathered skull (specimen YPM VP 3928; Schaeffer & Gregory, 1961; Schaeffer, 1967; Elliott, 1987). This species is distinguished by a non-hooked dentary and a pronounced ventral angle of the lachrymojugal (Brownstein, 2023), which is thought to be unique within *Whiteia* (Forey, 1998; Wendruff, 2011, PhD thesis; Yabumoto et al. 2019). However, examination of the available photographs (Brownstein, 2023, figs 1A, 2, 4) indicates that the shape of the dentary and lachrymojugal of YPM VP 3928 cannot be positively assessed because of their altered appearance, which prevents recognition of the boundaries of these bones. Unfortunately, we also consider that the other characters used to assign YPM VP 3928 to the genus *Whiteia* and distinguish it as a distinct species are too poorly preserved to allow recognition of a new species, a claim already made by Schaeffer & Gregory (1961). Brownstein (2023) noted the presence of ridges radiating from the ossification centers of the bones of the skull roof in YPM VP 3928. This ornamentation appears to be formed by altered coarse rugosities (Brownstein, 2023, Fig. 1C), a type of ornamentation known only in mawsoniids. Before being assigned to *Whiteia giganteus* (Brownstein, 2023), specimen YPM VP 3928 had already been examined by several authors (Schaeffer, 1967; Elliott, 1987; Forey, 1998), who assigned it to *Chinlea sorenseni*, an assignment not mentioned by Brownstein (2023). Based on the presence of the coarse rugosities ornamentation (albeit highly altered), which is the only reliable diagnostic character on YPM VP 3928, the assignment to *Chinlea sorenseni* is therefore possible. *Whiteia giganteus* should be considered a *nomen dubium*.”

**New text.** Lines 2333-2357 - (paragraph 5. Corrected and commented taxon scoring - *Diplurus*)

“Recently, Brownstein & Bissell (2022) erected the species *Diplurus enigmaticus* based on 5 specimens (the holotype YPM VPPU 14924 and the four referenced specimens 14939, 14943, 14949 and AMNH 15222). All these specimens had been previously referred to as *Diplurus newarki* by Schaeffer (1952). According to Brownstein & Bissell (2022), *D. enigmaticus* is distinguished by the combination of four diagnostic characters which are (1) a maximum standard length of about 150 mm (shared with *D. newarki*), (2) four angular foramina, (3) numerous (> 20) well-demarcated radiating ridges on the operculum and (4) a premaxilla with a reduced number (8) of enlarged conical teeth. Regarding the angulars illustrated by Brownstein & Bissell (2022, fig. 8), examination of the photographs suggests that the pores pointed to by arrowheads in *D. newarki* are not always correctly identified, and that their number is likely overestimated in all specimens (YPM VPPU 14558, 14918, 14929, 14933, 14944, 29366). In some specimens, the identified pores appear to be located on the splenial, and in other specimens, the arrowheads point to fissures in the bones. It appears that *D. newarki* has only four pores, as already indicated by previous authors (Schaeffer, 1952; Forey, 1998). Comparing the illustrations provided by Brownstein & Bissell (2022, fig. 9) and Schaeffer (1952, pl. 8 and 9), it appears that there is no variation in the size and shape of the teeth between *D. newarki* and *D. enigmaticus*. There is apparently only variation in the development of the dorsal lamina of the premaxilla, which is developed in some specimens (YPM VPPU 14924 and 14943) of *D. enigmaticus* and not in at least one specimen of *D. newarki* (YPM VPPU 14944, Schaeffer, 1958, pl. 9.1), resulting in a premaxilla represented only by its tooth-bearing portion. It is not clear whether the presence of numerous ridges on the opercle of *D. enigmaticus* has any specific significance (which is not the case according to Schaeffer (1952)), but since the association of this character with the number of angular foramens is here ruled out, we consider that this trait is too weak to allow the recognition of a new species. At this stage, further studies are necessary to determine whether the variation in these traits (premaxilla shape and ornamentation on the operculum) reflects individual or specific variations. Therefore, there are currently insufficient diagnostic characters to identify a new species within the available sample of *D. newarki*.”

The reviewer requested some more details about the reason why we didn’t retain the two taxa discussed about in our discussion.

**Complementary information**

We added below some complementary detailed explanation concerning the validity of *Whiteia giganteus* (Brownstein, 2023) and *Diplurus enigmaticus* (Brownstein & Bissell, 2022), which are summarized in the paragraphs added in our paper (above).

***Whiteia giganteus***

Brownstein (2023) erected the species *Whiteia giganteus* on the base of a single specimen represented by a poorly preserved skull (YPM VP 3928) from the Dockum Formation, Texas, USA. This skull has been first described by Schaeffer & Gregory (1961), who did not assign this specimen to a genus. Indeed, they wrote that “*Although the sandstone concretion preserves the external form of the skull, most of the dermal bones are incomplete, and their shape and form cannot be determined*” because this specimen represents a “*badly weathered skull*” (Schaeffer & Gregory, 1961, p.10). This highly weathered preservation is confirmed by the photos provided by Brownstein (2023, figs 1-4) and is also reported in other papers (Schaeffer, 1967; Elliott, 1987). Although the poor preservation of YPM VP 3928, Schaeffer & Gregory (1961) commented some characters of this skull proposing few comparisons with *Wihteia* and *Laugia* (which were in 1961 the only well-known Triassic species of coelacanths, including also *Diplurus*). Schaeffer & Gregory (1961, p.13) concluded that “*In the absence of really diagnostic characters, it is not possible to present any conclusions regarding the affinities of this unique specimen. Certain resemblances to genera such as Whitea or Laugia in the narrowness of the frontals or in general skull proportions can have little significance until more and better specimens are discovered*.” Such discoveries were done six years later when Schaeffer (1967) described *Chinlea sorenseni*. In his work, Schaeffer (1967, p.323) clearly refers YPM VP 3928 to *Chinlea sorenseni*. Ten years after, Elliott (1987) described another well-preserved and very large skull of *Chinlea sorenseni,* and discussed YPM VP 3928. Elliott (1987, p.51) wrote that “*It is clear now from comparison with the new specimen that the large skull from the Dockum should be assigned to Chinlea*” (even if the species name is not written here, Elliott implicitly assigned YPM VP 3928 to *Chinlea sorenseni*). In 1998, Forey referred YPM VP 3928 to *Chinlea sorenseni* without, indeed, a clear revision of this specimen. The specimen YPM VP 3928 was then referred to *Chinlea sorenseni* in three subsequent studies (Schaeffer, 1967, p.323; Elliott, 1987, pp. 48 and 51; Forey, 1998, p.305).

Brownstein (2023) never mentioned that YPM VP 3928 was first referred to *Chinlea sorenseni*, stating only at page 2 that the “*skull was preliminarily referred to the coelacanth †Chinlea sp. by Schaeffer (1967), an assignment that was agreed upon without extensive review of the specimen by Forey (1998)*”. The Elliott’s paper (1987), however, is omitted in Brownstein (2023)’s study. This author (2023) did not explain why YPM VP 3928 does not belong to *Chinlea sorenseni*. It is unlikely that Schaeffer (1967) would have attributed YPM VP 3928 to *Chinlea sorenseni* if there were any doubt about this attribution (same remark for Elliott, 1987). Forey's attribution of this specimen to *Chinlea sorenseni* (Forey, 1998) is also worth considering, as in this publication Forey only discussed coelacanth material that he had personally observed and studied (if he referred to a specimen of a species but hasn't examined the material directly, he clearly indicated this point).

According to Brownstein (2023), *Whiteia giganteus* is assignable to the genus *Whiteia* based on the combination of 10 characters that are: (1) the presence of an elongated preorbital region more than 1/3rd the length of the total skull roof, (2) a short, widened postparietal shield and mediolaterally compressed parietonasal shield, (3) a first anterior supraorbital that excludes the preorbital from the orbital margin, (4) a lachrymojugal that is deflected ventroanteriorly at its anterior end, (5) a large, rounded opercle with a strongly pointed ventral terminus, (6) a cheek composed of the abutting lachrymojugal, postorbital, preopercle, and squamosal, (7) a small subopercle, (8) a shallow mandible, (9) gular plates that terminate anterior to the posterior terminus of the mandibles and (10) a sparse ornamentation of the skull dermal bones; and distinguished by 9 characters that are (1) a large (>1 m) size, (2) posterior pterygoid teeth grow to nearly twice as large as anterior crowns on tooth plate, (3) an enlarged angular foramina row, (4) the anterior terminus of lachrymojugal reaches discrete apex, (5) a lachrymojugal with a pronounced ventral angle below the orbit, (6) a dentary that is not hook-shaped, (7) a prefrontal equal to less than 15% of total skull length (8) a parietonasal shield with a flat to convex lateral profile and (9) a straightened angular foramina row.

Unfortunately, most of these characters present problems in their description and illustrations (it should be pointed out that the photos are in a high-quality resolution). Indeed, there are some discrepancies between the text and what can be seen in the photos. Here, we will discuss just a few of them.

Brownstein (2023, p.4) mentions that *Whiteia giganteus* belongs to *Whiteia* because it has “*a first anterior supraorbital that excludes the preorbital from the orbital margin*”. Regarding the photos (Brownstein, 2023, figs1-2), it is clear that there is no bone that can be identified positively as a preorbital (e.g. with foramens for the posterior openings of the rostral organ) located dorsally to the anterior part of the laychrymojugal of YPM VP 3928. Moreover, such a peculiar character has never been observed or mentioned in any known coelacanth included in the genus *Whiteia* (Forey, 1998; Wendruff, 2011, PhD dissertation; Yabumoto et al. 2019).

*Whiteia giganteus* is distinguished from other *Whiteia* by having “*a lachrymojugal with a pronounced ventral angle below the orbit*” (Brownstein, 2023, p.4), which is another intriguing character. Indeed, such a lachrymojugal is known only in some few and peculiar coelacanth such *Foreyia, Rieppelia, Ticinepomis, Serenichthys* and *Diplocercides kayseri* (Stensïo, 1937; Forey, 1998; Gess & Coates, 2015; Ferrante et al. 2023; Ferrante & Cavin, 2023; character 43 in our article). Unfortunately, regarding the photos (Brownstein, 2023, figs 1A-2), it clearly appears that this bone is badly damaged, with the entire lower portion completely weathered, making it difficult to appreciate its exact overall shape. However, the global shape of the lachrymojugal of YPM VP 3928 recalls more the one of *Chinlea sorenseni* (Schaeffer, 1967, pl. 28; Elliott, 1987, figs 1-3) or other coelacanths such as *Axelrodichthys araripensis* (Maisey, 1986, fig.26A), another mawsoniid coelacanth. Its shape is rather “usual” and is not similar to that of the peculiar coelacanths cited above. Furthermore, the very bad preservation of the lachrymojugal of YPM VP 3928 precludes to use this feature as a diagnostic character.

Brownstein (2023, p.2) wrote that *Whiteia giganteus* “*Differs from †Whiteia nielseni and †W. uyenoteruyai, but shares with †W. woodwardi, †W. tuberculata, and †W. africana a dentary that is not hook-shaped*”. However, all these cited species of *Whiteia,* including the type species *W. woodwardi*, have a dentary hooked-shaped (Forey, 1998; Wendruff, 2011, PhD dissertation; Yabumoto et al. 2019; character 58 in our article here). Moreover, Wendruff (2011, PhD dissertation, p.87) emended the diagnosis of *Whiteia* and mentioned that *Whiteia* has, among other diagnostic characters, a hook-shaped dentary. Based on the photos (Brownstein, 2023, fig.4), the shape of the dentary of YPM VP 3928 cannot be positively assessed because it is covered on its dorsal margin by sheets of bone or sediment.

The small subopercle is not described in the text and is labelled with a question mark as “*subopercle? (mold)*” (Brownstein, 2023, fig. 1A).

The ornamentation of YPM VP 3928 is probably the only reliable visible character. Brownstein (2023, p.2) mentioned that *Whiteia giganteus* presents a “*poorly ornamented skull characteristic of †Whiteia that differentiates it from other large Mesozoic coelacanths*”. However, there are at least 10 species of Mesozoic coelacanths having a skull not or poorly unornamented that are *Coccoderma, Diplurus, Garnbergia, Guizhoucoelacanthus, Holophagus, Heptanema, Libys, Megalocoelacanthus*, *Swenzia* and *Undina*. Brownstein (2023, p.5) then wrote that “*Ornaments on the postparietals are the best preserved of those from the skull roof and consist of ridges radiating from ossification centers*”, a feature that can be indeed observed on the photo (Brownstein, 2023, fig. 1C). However, most of the skull bones of all *Whiteia* species are smooth with sometimes a few tubercles (e.g. Forey, 1998) and no “*ridges*” have yet been reported in any *Whiteia* species. The kind of ornamentation on the skull of YPM VP 3928 is clearly identifiable as coarse rugosities that have been weathered. Such an ornamentation pattern is observed only in mawsoniids, such as *Chinlea sorenseni*. The last observation supports the assignment of YPM VP 3928 to *Chinlea sorenseni,* as already stated by Schaeffer (1961), Forey (1998) and Elliott (1987).

Based on all previous remarks, the assignment of YPM VP 3928 to *Whiteia giganteus* is based on no reliable diagnostic or not properly described characters. The reliability of *Whiteia giganteus* is questionable and this species should be then seen as a *nomen dubium*.

The opinion of the first author (CF) is that YPM VP 3928 represents a mawsoniid, most likely *Chinlea sorenseni*, particularly because of the bones’ ornamentation. This view is in agreement with that of previous authors (Schaeffer, 1967; Elliott, 1987; Forey, 1998).

*Diplurus enigmaticus*

Brownstein & Bissell (2022) erected *Diplurus enigmaticus* on the basis of 6 specimens (the holotype YPM VPPU 14924 and the 5 referred specimens 14939,14943, 14949, 14558b and AMNH 15222) that were previously referred by Schaeffer (1952) to *Diplurus newarki*. In their study, Brownstein & Bissell (2022) “*quantify phenotypic disparity and species richness in a sample of over 500 individual coelacanths from a single locality*”. At this point, it seems that *Diplurus enigmaticus* is a quite rare species in this locality as only 6 specimens have been identified over more than 500 specimens.

According to Brownstein & Bissell (2022, pp.6-7), *Diplurus enigmaticus* is distinguished by the combination of 4 diagnostic characters that are (1) a maximum standard length of approximately 150 mm (shared with *Diplurus newarki*; 690 mm in *D. longicaudatus*), (2) four angular foramina (zero to two in *D. longicaudatus*; five or more in *D. newarki*), (3) numerous (>20) well-delimited radiating ridges on opercle (maximum of seven observed in *D. newarki*; irregular lineations and tubercles present in *Diplurus longicaudatus*) and (4) a premaxilla with reduced number (8) of enlarged, conical teeth (11 in each element in *D. newarki*). Brownstein & Bissell (2022) concluded by writing that “*Apart from the features noted in the diagnosis section, †Diplurus enigmaticus individuals fall within the range of variation observed in †Diplurus newarki and †Diplurus longicaudatus*”.

The difference between *Diplurus longicaudatus*, on the one hand, and both *Diplurus newarki* and *Diplurus enigmaticus*, on the other, seems to be particularly marked (Tab. 1). In contrast, the difference between *Diplurus newarki* and *Diplurus enigmaticus* appears to be less marked.

|  | *Diplurus newarki* | *Diplurus enigmaticus* | *Diplurus longicaudatus* |
| --- | --- | --- | --- |
| ST length (mm) | 150 | 150 | 690 |
| Nb of “angular foramina” | 5 or more | 4 | 0-2 |
| Ornamentation on the opercle | well-delimited radiating ridges (max of 7) | well-delimited radiating ridges (>20) | irregular lineations (numerous weak ridges) and tubercles |
| Nb of pmx teeth | 11 | 8 | ? |

**Tab. 1.** Characters mentioned in the diagnosis of *Diplurus enigmaticus* by Brownstein & Bissell (2022).

(1) The maximum standard length of *Diplurus enigmaticus* is shared with *Diplurus newarki* and only differs greatly from that of *Diplurus longicaudatus*. This character is thus not a specific character that can serve to distinguishes *Diplurus enigmaticus*.

(2) Schaeffer (1952, p.45) first described the angular of *Diplurus newarki* and wrote that on the angular “*There are four large sensory canal pores distributed along the posterior surface*”. Including the “*three or more large pores on*” the splenial, the mandibular sensory canal of *Diplurus newarki* thus opens through a total of at least 7 pores. In his review of *Diplurus newarki*, Forey (1998, p.151) wrote that “The mandibular sensory canal opens through four posteroventrally directed pores within the angular and through two pores within the splenial, in addition to opening through the offset dentary pore”.

Brownstein & Bissell (2022, fig.8) observed five and more pores (their foramina) on the angular of *Diplurus newarki* and only four on *Diplurus enigmaticus*. They illustrated this feature with 6 specimens referred to *Diplurus newarki* (YPM VPPU 14558, 14918, 14929, 14933, 14944, 29366) and 3 specimens referred to *Diplurus enigmaticus* (YPM VPPU 14924, 14949, 14939). We agree that there are four pores on these 3 latter specimens (YPM VPPU 14924, 14949, 14939). Our interpretation of the situation in the other specimens, however, differs from those of Brownstein & Bissell (2022). The photos on the figure 8 of Brownstein & Bissell (2022) shows that the pores are likely overestimated in all specimens of *Diplurus newarki* (YPM VPPU 14558, 14918, 14929, 14933, 14944, 29366). In YPM VPPU 14929, 6 pores are pointed out, but the 2 anterior most pores are open on the splenial (one posterior pore is incorrectly pointed); there are thus 4 pores. In YPM VPPU 14933, 5 pores are pointed out but the anterior most pore is on the splenial; there are thus 4 pores. The angular of the specimens YPM VPPU 14918, 14944 and 29366 is crushed in such a way that there are many cracks, preventing a correct and proper identification of the pores. Moreover, in the last three specimens, some of the features identified as pores are in fact clearly cracks. In the specimen YPM VPPU 14558, 6 pores are pointed but the anterior most pore is clearly the one occurring between the angular and splenial and is not, properly speaking, on the angular. However, this specimen is the only one with a small variation. In the posterior part, there is 1 large pore with, posterior to it, 1 small pore and, anterior to it, 2 small pores separated by a thin bony bar. There is 1 additional small pore occurring in the most anterior part of the angular. The specimen YPM VPPU 14558 is thus the only specimen showing apparently 5 pores (4 pores in the posterior part as usual in *Diplurus newarki*, plus 1 extra small pore in the anterior part). However, this variation is probably rather an individual variation than a specific variation (i.e. the extra small pore having opened on the angular rather than the spenial).

Based on these remarks, *Diplurus newarki* appears to not have 5 or more pores on the angular but only 4 pores as already stated by previous authors (Schaeffer, 1952; Forey, 1998). Consequently, there are no difference in the number of pores between *Diplurus newarki* and *Diplurus enigmaticus.*

(3) Schaeffer (1952, p.42) described the opercle of *Diplurus newarki*, writing that the ornamentation is made of “*low radiating striae. This type of ornamentation, when present, is always confined to the posterior border. The striae, however, may have a more general distribution. In a few specimens of D. newarki from the Princeton locality, there are five to 20 short ridges on the dorsal half of the opercular and, according to Shainin (1943), some specimens of this species from Granton may have sparsely distributed tubercles*”. Forey (1998, p.313) did not dwell on the description of the type of ornamentation in *Diplurus*, stating only that it varies (on bones and scales) between *Diplurus longicaudatus* and *Diplurus newarki* and stated that the opercle of *Diplurus longicaudatus* is entirely covered with ornament while in *Diplurus newarki* the ornament is confined to the dorsal half of the opercle.

Brownstein & Bissell (2022, p.9) wrote that their “*quantification of key skull characteristics in coelacanths from both Firestone Library and Granton Quarry shows that small-bodied coelacanths with heavily striated opercles also consistently possess four angular foramina, whereas †Diplurus newarki consistently possesses fewer than 10 opercle striations and five angular foramina*”. As demonstrated above, *Diplurus newarki* does not have five but only four pores on the angular. There is therefore no link between the number of pores on the angular and a difference in the ornamental pattern on the opercle in *Diplurus newarki*, as claimed by Brownstein & Bissell (2022). This conclusion is thus similar to the conclusion of Schaeffer (1952, p.42), who writes that “*The variations in opercular ornamentation of D. newarki are clearly not of taxonomic significance*”.

Furthermore, as other authors have already mentioned (Guess & Coates, 2015; Ferrante et al. 2023, our article), the ornamentation is a character that must be used with caution in phylogenetic analyses. Indeed, this character can present variations between individuals and with ontogenetic stages (using the number of striae to differentiate species is therefore clearly irrelevant).

(4) The description and illustration of the coelacanth premaxilla is very important, as the snout bones, and in particular the premaxilla and the teeth, are poorly known in this group. Indeed, in most coelacanth fossils, the premaxilla is either not preserved or only poorly preserved.

Schaeffer (1952, p.37, pls 8 and 9) first described the premaxilla of *Diplurus newarki* using two well-preserved specimens (YPM VPPU 14943 and 14944). Unfortunately, he didn’t describe the size of the teeth and only mentioned in the diagnosis page 53 “*Premaxillary small, splint-like, with teeth*”. Following the description of Schaeffer (1952), Forey (1998) only mentioned that the premaxillae are preserved as narrow splints bearing very small teeth.

Brownstein & Bissell (2022, fig.9) illustrated the difference between premaxillae of *Diplurus enigmaticus* and *Diplurus newarki* using three specimens (YPM VPPU 14924, 14944, 29366). First, it is disturbing to note that the specimen figured and labelled as YPM VPPU 14944 by Brownstein & Bissell (2022, fig.9) is not the same that the one labelled as YPM VPPU 14944 and figured by Schaeffer (1952, p.37, pl. 9.1). Moreover, the two “*small premaxilla*” of *Diplurus newarki* (YPM VPPU 14944, 29366) illustrated by Brownstein & Bissell (2022, fig.9) appear small likely because of their mode of preservation being covered by sediment or other snout bones.

Nonetheless, the holotype of *Diplurus enigmaticus* (YPM VPPU 14924) shows indeed a “larger” (in the height) premaxilla with 8 small conical teeth. However, it appears that the premaxilla is not fully visible, as its medial part is overlapped by the supraorbital series. In addition, as the teeth appear to be regularly spaced, it is obvious that at least one tooth (possibly more) is missing on the most medial portion. The specimen YPM VPPU 14943 referred by Brownstein & Bissell (2022) to *Diplurus enigmaticus* and illustrated by Schaeffer (1952, p.37, pls 8 and 9) shows a similar “high” premaxilla with very small teeth. Seven teeth are preserved here but there is the space for at least 10 to 11 teeth. Regarding the teeth of the specimen YPM VPPU 14943 of *Diplurus enigmaticus* (Schaeffer, 1952, pl. 8), there is in fact any difference (in shape or size) with the specimen YPM VPPU 14944 of *Diplurus newarki* (Schaeffer, 1952, pl. 9.1).

At this point, the only difference seems to be the bony development of the premaxilla observed in two specimens of *Diplurus enigmaticus* (YPM VPPU 14924 and 14943). This bony development is known as the dorsal lamina of the premaxilla (Forey, 1998; our character 5). Some coelacanths have either a premaxilla with a dorsal lamina, or a premaxilla with only a tooth-bearing portion. Unfortunately, the development of the dorsal lamina of premaxilla and its relationships with the neighboring bones (rostral bones) is currently poorly understood. The only point that can be brought is that the snout bones tend to fuse in some coelacanths (e.g. *Macropoma*), which may be a specific or individual variation (e.g. Forey, 1998). Schaeffer (1952, fig.5) and Forey (1998) have already demonstrated that *Diplurus newarki* shows a variation in the bone pattern of the parietal shield, with the skull bones tending to fuse. The development of the dorsal lamina observed in the two specimens referred as *Diplurus enigmaticus* could be an intraspecific difference rather than a specific variation. The development of the dorsal lamina of premaxilla in coelacanth could also be produced by the fusion of the premaxilla and the neighboring bones (i.e. the small rostral bones) but this statement requires further studies.

Consequently, the premaxillary teeth of *Diplurus enigmaticus* are not different (in size and shape) to that of *Diplurus newarki* and the presence of the dorsal lamina cannot yet be used with certainty as a specific character to properly distinguish *Diplurus enigmaticus* as a distinct species (in fact this last feature is not mentioned in the diagnose made by Brownstein & Bissell, 2022). There is thus no significant difference between the teeth and premaxilla of *Diplurus enigmaticus* and *Diplurus newarki*.

In summary, (1) the maximum standard length does not differentiate *Diplurus enigmaticus* from *Diplurus newarki*, (2) the number of pores on the angular of *Diplurus enigmaticus* is similar to that of *Diplurus newarki*, (3) the ornamentation on the opercle is of no specific significance and (4) the number and size of premaxillary teeth do not differentiate *Diplurus enigmaticus* from *Diplurus newarki*. Consequently, there are no diagnostic characters that can positively allow the recognition of *Diplurus enigmaticus* as a distinct species. The reliability of *Diplurus enigmaticus* is therefore questionable and this species should probably be considered as a *nomen dubium*.

To conclude this extensive answer, we agree that it would have been a mistake not to mention *Whiteia giganteus* (Brownstein, 2023) and *Diplurus enigmaticus* (Brownstein & Bissell, 2022), and we believe that our comments will serve the literature and future research on coelacanths. We therefore thank the reviewer for pointing out the need to cite and comment these two articles.

**Reviewer:**

2) The introduction and discussion need to be expanded. Although I understand that this paper is primarily a follow-up to the other recent work by the authors, it needs to be better set in the context of the literature on coelacanths and similarly old groups of 'fishes' (lungfishes, sturgeons and paddlefishes, gars and bowfin, bichirs).
Additional discussion of the 'living fossilhood' of coelacanths is needed, for example, because this is essential to your point on stability. Some key studies that should be cited include Casane and Laurenti (2013) on coelacanth 'living fossilhood' (you cite the response paper to this but not the original, and it is always good scholarship to review both particularly since the response is by one of the authors of the current manuscript) as well as a couple of recent papers that have looked at morphological and molecular evolution in living fossils:
Meyer et al. (2021), Nature : the Neoceratodus genome
Schartl et al. (2024), Nature : the Lepidosiren genome
Wang et al. (2021), Cell: the Protopterus genome
Brownstein et al. (2024), Evolution showing molecular stasis in gars and sturgeons but not coelacanths and lungfishes

# Answers to comment No°2

We reworked and extended the introduction and conclusion as requested (see revised text at lines 46-114 and 5137-5165). We also included some of the suggested literature (i.e. Casane & Laurenti 2023; Brownstein et al., 2024).

However, we do not consider that some of the suggested papers (Meyer et al. 2021; Schartl et al., 2024; Wang et al., 2021) should be cited here, because their topic strays too far from the theme of our article. The aim of our paper is not to discuss morphological characters triggered by molecular evolution in ‘living fossils’. As you correctly pointed out, this paper is a follow-up to our recent work (Ferrante & Cavin, 2023), in which we already discussed some ideas on the morphological character triggered by molecular mechanism.

We have made it clearer that this work is a continuation of our previous work (see in the text).

We have also added a final paragraph in the conclusion dealing with future research directions.

**Reviewer**

As it is, the discussion makes it seem like only a handful of authors have considered the evolutionary history of coelacanths (both timescale and morphology-based relationships). A lot more has been done than that, simply put. For example, the timescale of coelacanth evolution is something you figure in this study but do not discuss at any length in the introduction or discussion. The results of Clement et al. (2024) in Nature Communications for example should be discussed at greater length than a short paragraph at the end of the discussion, especially since you all produce somewhat different hypotheses of the timescale of coelacanth evolution. From the last paragraph, it seems like this paper was rapidly worked after the publication of that paper, which is understandable, but in a revision I would like to see some additional engagement with that paper. Particularly, your phylogeny appears to support a very rapid diversification of coelacanths at the Permian-Triassic mass extinction. This is super interesting and definitely matches expectations from the authors' previous work on the Triassic coelacanths of Switzerland. I would like some more text to focus on the time tree and its implications for our understanding of coelacanth evolution, and this is one detail I would definitely appreciate more information about given that Clement et al. (2024) do not recover anywhere near as pronounced a signature of Permo-Triassic diversification. Would this support coelacanths as Elvis Taxa?

We added a paragraph “6.4. Time-scaled phylogeny” describing with more information and references to previous studies (see revised text).

We agree that our comments on the Clement et al. paper were somewhat brief. Therefore, we included the new genus in our phylogenetic analysis. Ngamugawi's character scoring was based on a detailed review of the characters used in the Clement et al. paper, with some comments and corrections for some scoring. We added a new paragraphs dedicated to Ngamugawi.

**New text.** Lines 3085-3149 (paragraph 5. Corrected and commented taxon scoring - *Ngamugawi*)

“Ngamugawi

Types and only species: N. wirngarri, early Frasnian (Late Devonian), Western Australia [13].

This genus was recently described and scored by Clement et al. [13] on the basis of two well-preserved specimens (lacking most of the posterior portion of the skeleton) from the Gogo Formation in Western Australia. We scored Ngamugawi based on information from Clement et al.’ datamatrix [13], except for the characters discussed below. For characters present in our list only, we completed the scoring based on description and illustrations of Clement et al. [13].

Ngamugawi has one of the best-preserved premaxilla ([13], fig. S1C) of any known fossil coelacanth. The premaxilla bears a well-developed dorsal lamina and a foramen for the anterior rostral organ, and five preserved teeth plus at least two missing teeth along its labial margin. Ngamugawi has thus a total of at least seven premaxillary teeth (character 4), which is the plesiomorphic state.

On the parietonasal shield of Ngamugawi, Clement et al. [13] identified one pair of large posterior parietals and a pair of smaller parietals (‘preparietals’ in Clement et al. [13]; characters 8 and 9).

On the anterior margin of the orbit is a large bone pierced by two foramens. Although these two foramens are identified as the anterior and posterior openings of the rostral organ ([13], fig. 1G-F), this large bone, clearly in the position of the preorbital, is not identified in their work. This large bone is aligned with the supraorbital series, and is in direct contact with the parietal series, thus being not separated from the supraorbital-tectal series as is the preorbital in most coelacanths ([13], 2024, figs 1A-B, F-G). A similar situation has already been reported in Axelia and Wimania by Stensiö [66], who identified in the anterior margin of the orbit a ‘nasal-antorbital’ bone, an ossification that Forey [7] named ‘tectal-preorbital’. Although Forey [7] did not refute Stensiö's observation, he concluded that this 'unusual' restoration of the snout in Wimania and Axelia is uncertain pending the description of new material. A similar case also occurs in both species of Euporosteus. E. yunnanensis does not have a distinct preorbital bone ([2], fig. 3a-e] and in E. eifeliensis, our reconstruction (Fig 42) suggests that the preorbital is not separate from the supraorbital-tectal series, similarly to Ngamugawi. Further studies are needed to better understand this peculiarity and the relationship of the preorbital bones with the supraorbital and tectal series. We have therefore left the condition of the presence of a preorbital as unknown in Ngamugawi (character 13) but we have nevertheless scored the characters dealing with the posterior openings of the rostral organ (characters 46 and 47).

We scored the posterior margin of the postparietal shield of Ngamugawi as straight (character 17) ([13], fig. 1A, F], as in Diplocercides (Fig 39A) for example, unlike Clement et al. [13] who scored it as embayed.

Clement et al. [13] described and illustrated the supraorbital sensory canal passing through the sutures between the supraorbitals and the parietals but scored this canal as running through centre of ossification. We therefore corrected this scoring (character 22).

According to our definition of the postorbital position, this bone is located behind the level of the intracranial joint (character 34). Indeed, in Ngamugawi, the anterior margin of the postorbital is located at the very limit of the intracranial articulation as in Diplocercides (Figs 13, 39B) and is not spanning the intracranial joint [13] as in Chinlea or Mawsonia (Figs 13, 35B, 54B), for example.

Clement et al. ([13], fig. 1G) reconstructed the parietonasal and postparietal shields of Ngamugawi with a very straight profile in lateral view, thus making the squamosal without contact with the postparietal bone. If a shallow angle had been marked between the two halves of the skull roof in the head reconstruction ([13], fig. 1G), the squamosal would have been in almost direct contact with the postparietal, making this condition similar to that of Diplocercides (Fig 39). Consequently, the condition of the squamosal of Ngamugawi is scored as ‘extending behind the postorbital to reach the skull roof’ (character 37).

Clement et al. [13] described the lachrymojugal of Ngamugawi as an elbow-shaped bone expanded ventrally similar to that of Diplocerides kayseri and Serenichthys. However, they scored the condition of the posteroventral extension of the lachrymojugal of Ngamugawi as ‘absent’ while they scored it as ‘present’ in D. kayseri and Serenichthys. The posteroventral extension of the lachrymojugal of Ngamugawi ([13], fig.2A-D) appears to be poorly developed, as in Serenichthys ([29], fig.2). Consequently, we scored the condition of the lachrymojugal of Ngamugawi (character 43) similarly to that of Serenichthys and Diplocerides.

According to Clement et al. [13], the jugal sensory canal opens within the squamosal through a few large pores running along the ventral margin of the bone, sending out prominent branches. However, the squamosal of Ngamugawi is illustrated ([13], fig.1 A, G) with a simple jugal sensory canal that runs in the middle of the bone (character 51) without prominent branches (character 50). Furthermore, the pores are rather small (character 52) compared to the size of the canal itself and to the pores of the supraorbital canal.

On the lower jaw of Ngamugawi, there are a posterodorsal glenoid fossa and a posterior articulation point articulating respectively with the palatoquadrate and symplectic [13]. The CT-scans of the lower jaw ([13], fig. 2K-N) show that there are no clear distinct retroarticular and articular bones. Ngamugawi seems to present rather the plesiomorphic condition with a co-ossified retroarticular-articular bone (character 57) as in many other Palaeozoic coelacanths such as Diplocercides, Caridosuctor, Allenypterus or Rhabdoderma ([7], figs 5.2, 5.3) for instance.”

We have modified the description of the trees and their related figures; Fig 73, 74, 75 and 76, as well as the list of support nodes in chapters 6 and 7 according to the new tree obtained (not many changes). See the text for details.

We also added in the discussion (chapter 8) the following comment:

**New text.** Lines 5053-5076 + 5120-5125.

“ Recently, Clement et al. (2024) described a new coelacanth from the Upper Devonian Gogo Formation of Western Australia and provided a new phylogeny based on a new data matrix that includes many corrections and new characters [132]. This character revision was conducted in parallel with our own revision, making it impossible to integrate the information they used into our own study. However, we briefly discuss their results, particularly in relation to diversity dynamics at the Permian–Triassic boundary. As in Toriño et al. (2021) and in the present study, Clement et al. (2024) found a peak of diversity in the Triassic (their Figure 3; unfortunately, in this figure, most of the Early Triassic occurrences are graphically located in the Late Permian). In all three studies, the Triassic diversity peak is formed partly by basal Coelacanthiformes (mainly Laugiidae, Whiteiidae and Axeliidae), with taxa present in the Early Triassic, and partly by Latimerioidei, with taxa present mainly in the Middle Triassic. Bayesian inference indicates that the diversification driving the Triassic diversity peak occurred in the Permian, and that the rate of evolution of discrete, meristic and continuous characters is rather low in the Triassic compared to other time intervals (Clement et al., 2024, fig. 4). The study of Toriño et al. (2021), based on a time-scale parsimonious analysis, showed that the peak of cladogenetic events occurred in the Middle Triassic, concomitant with a drop in the average ghost range. This pattern is consistent with the presence of a true diversification event (Toriño et al., 2021; Cavin & Forey, 2007). Based on this information, we hypothesize that Early Triassic coelacanths were mainly representatives of ancient lineages that survived the end-Permian mass extinction and rapidly diversified and occupied new ecological niches (Dai et al., 2023), followed by further diversification in the Middle Triassic particularly, but not exclusively, within the Ticinepomiinae (Cavin et al., 2017; Ferrante & Cavin, 2023). Future studies focusing on a more detailed analysis of events within the Triassic, with a focus on the rate of character evolution, should allow us to better understand the succession of events that characterized the evolutionary history of coelacanths during this time interval.”

[…]

We note some divergence between the phylogeny of Clement et al. (2024) and ours. For example, *Foreyia* is considered a sister species of *Indocoelacanthus* outside the latimerioidei in their study, whereas we considered it a basal latimeriid. This divergence is likely due, at least in part, to the fact that Clement et al. [132] did not include *Rieppelia* in their analysis, as well as the new characters associated with this genus [4]. We encourage the production of a new character data matrix that will merge the data from both studies.”

Regarding the "Elvis taxon", we do not believe that coelacanths fit this concept: “Elvis taxon is a taxon that has been misidentified as having re-emerged in the fossil record after a period of presumed extinction, but is not actually a descendant of the original taxon, instead having developed a similar morphology by convergent evolution” Wikipedia. (See also « Lethiers, F., & Casier, J. G. (1999). Taxons “Lazares” et taxons “Elvis”: à utiliser avec circonspection. Geobios, 32(5), 727-731” for a more academic discussion about these terms).

Regarding the following reviewer comment “From the last paragraph, it seems like this paper was rapidly worked after the publication of that paper, which is understandable, but in a revision I would like to see some additional engagement with that paper”, we would like to explain our position. This paper is a chapter of the first author’s thesis, which was approved by a thesis committee. The paper was almost ready several months ago, and was not “rapidly worked after the publication of that paper”. It is true that we submitted the paper quickly after the publication of the Clement et al's paper, because a long delay would have made it necessary to discuss more in depth the phylogenetic analysis conducted by our colleagues. This is a fairly common situation when two papers including a phylogenetic analysis are published within a short time interval. In fact, the situation was similar for our colleagues: The Clement et al's paper was submitted to Nature Communications more than a month after our work about *Rieppelia* was available (December 5, 2023 and October 20, 2023, respectively) (it presents a similar set of characters to those used in this paper). Our paper was not cited and its data were not discussed. We understand our colleagues' decision because of the consequences it could have had on their own paper. We have absolutely no problem with this situation but consider it useful to highlight this point here. We hope that the more detailed discussion of the Clement et al. paper presented in this revised version meets the reviewers' expectations.

**Answers to some of your specific remarks in comment No. 2:**

1) “A monophyletic family/clade is oxymoronic”

Done

2) ‘the term “basalmost” doesn't really mean anything/communicate anything in phylogenetics’

We have modified the 3 occurrences containing this term as follows:

Lines 35-37 (abstract): New sentence reads: “All Mesozoic coelacanths, including extant Latimeria, are resolved as members of the order Coelacanthiformes, a clade that arose in the Permian, with Coelacanthus diverging first.”

Line 3686 (paragraph 6.3.2.): We identify this node as the Miguashaiidae, which is the first family of coelacanths to diverge.

Lines 3874 (paragraph 6.3.6.): Our phylogenetic analyses resolved *Coelacanthus* as the first species to diverge within the Coelacanthiformes, (…).

Line 3977 (paragraph 6.3.10.): In the first diverging genera of the Latimeriidae and Mawsoniidae, namely *Dobrogeria*

Line 4404 (paragraph 6.3.16.): However, this character is unknown in the first diverging Latimeriidae *Dobrogeria*.

3) ‘itself divided into ... themselves divided into’ creates a run-on sentence

We reformulate the sentence as follow:

Lines 17-44 (abstract): “…, each of which is divided into two subfamilies”.

4) ‘the understanding of coelacanth phylogeny’ can be edited to ‘coelacanth phylogeny’.

done

**Reviewer:**

3) Figure 72 is entirely illegible as submitted and needs to be remade at higher resolution. I also recommend changing the brightness values in several of the real color images of textures; as it is these are difficult to see.

# Answer to comment No°3

In the pdf there is a hyperlink that opens a high-resolution version of the table.

Nevertheless, we have modified the figure 72 showing the matrix because of the addition of the new taxon of Clement et al.

We have also added a large-sized version of figure 72 showing the matrix in the supporting information section (S2, section 2.3).

**Reviewer**

4) It is perhaps almost cliché to ask for this, but I would like the authors to expand the reportoire of phylogenetic analytical protocols employed in this manuscript to include Bayesian methods. Bayesian analyses are commonplace now in phylogenetics and are widely applied to morphological datasets. One can also jointly infer a timescale of evolution and relationships in some programs (e.g., BEAST2). The authors should at the very least run an uncalibrated Bayesian analysis (say, in MrBayes or RevBayes) to see whether the phylogeny that they present in this study is found using different methodologies.

Indeed, one of the pushes of the current study is that the stability of coelacanth phylogeny might be due to the repeated use of the same dataset. An analogous issue is the repeated use of one criterion (parsimony) as the method of inference. Clement et al. (2022) and Brownstein and Bissell (2022) both conducted Bayesian tip-dating analyses on the modified Forey dataset and resolved different trees than they did when they used parsimony. In order to be able to pronounce coelacanth phylogeny stable, the authors really should be exploring multiple methods of inference.

# Answer to comment No°4

Indeed, rather than extending this research using the Bayesian method, which would rather be a way of comparing the methods themselves than of looking further for phylogenetic relationships, we compare the results obtained by Clement et al. (2024) with ours, with the inclusion of *Ngamugawi*. Because the latter search is based at the species level, and especially because it does not include an important taxon (*Rieppelia*) and its associated set of characters, a Bayesian analysis of our data will not allow us to compare our results.

We added in the discussion the text mentioned above (lines 5053-5076)

**Additional modifications (not requested by the reviewer)**

- In the first version, the caption of one figure was incorrect: Fig 71 *Onychodus jandemarrai* was erroneously numbered as Fig 70. We corrected this issue.
- We have modified the diagnosis of the Diplocercidae to reflect the inclusion of *Ngamugawi* in this clade.
- In the first version, we mentioned that we score *Libys* including *L. polypterus* and L*. callolepis*. However, we forgot to mention which characters we added from *L. callolepis* in the scorings. We corrected this issue (see text and matrix).
- In the first version, we had indicated a reference to a "Table 1" which is, however, absent from our article. Instead of adding a new figure, we have added a new supporting information (S3) file that includes the calculations and the results that should have appeared in the missing “table 1”.
- We modified the title of the chapter 6 as “6. Results and discussion”.
- We modified the title of the chapter 6.2. as “6.2. Most parsimonious trees and Consensus tree” because of the addition of the new chapter “6.4 Time-scaled phylogeny”.
- We made some few corrections (layout, syntax) and reworked some very few sentences for better clarification.
- In the supporting information S2 and S4, we added the references for the cited literature and for the illustrations.
- We corrected the figures 29, 45 and 63 showing *Allenypterus,* *Gavinia* and *Serenichthys*, respectively.
- We have added a precision to state 0 of the definition of character 27 (because of a coding error that we have notified and corrected for *Diplocercides*).
- In the supporting information S4, we deleted some illustrations of osteichthyans skull that are not relevant (*Amia*, *Eusthenopteron*, *Glyptolepis*, *Strunius*). We also added some illustrations that were not visible (draw layers were not activated when the figures were generated) on the first version (*Yunnancoelacanthus*, the skull roof bones; *Propolepis*, the cheek bones; *Mimipiscis* and *Guiyu*, their lower jaw).

Thanks again for the detailed and constructive comments on this paper!

The authors
